# ClinPharmSeq: A targeted sequencing panel for clinical pharmacogenetics implementation

**DOI:** 10.1371/journal.pone.0272129

**Published:** 2022-07-28

**Authors:** Seung-been Lee, Jong-Yeon Shin, Nak-Jung Kwon, Changhoon Kim, Jeong-Sun Seo

**Affiliations:** 1 Macrogen Inc, Seoul, Republic of Korea; 2 Asian Genome Center, Seoul National University Bundang Hospital, Gyeonggi-do, Republic of Korea; International Medical University, MALAYSIA

## Abstract

The accurate identification of genetic variants contributing to therapeutic drug response or adverse effects is the first step in implementation of precision drug therapy. Targeted sequencing has recently become a common methodology for large-scale studies of genetic variation thanks to its favorable balance between low cost, high throughput, and deep coverage. Here, we present ClinPharmSeq, a targeted sequencing panel of 59 genes with associations to pharmacogenetic (PGx) phenotypes, as a platform to explore the relationship between drug response and genetic variation, both common and rare. For validation, we sequenced DNA from 64 ethnically diverse Coriell samples with ClinPharmSeq to call star alleles (haplotype patterns) in 27 genes using the bioinformatics tool PyPGx. These reference samples were extensively characterized by multiple laboratories using PGx testing assays and, more recently, whole genome sequencing. We found that ClinPharmSeq can consistently generate deep-coverage data (mean = 274x) with high uniformity (30x or above = 94.8%). Our genotype analysis identified a total of 185 unique star alleles from sequencing data, and showed that diplotype calls from ClinPharmSeq are highly concordant with that from previous publications (97.6%) and whole genome sequencing (97.9%). Notably, all 19 star alleles with complex structural variation including gene deletions, duplications, and hybrids were recalled with 100% accuracy. Altogether, these results demonstrate that the ClinPharmSeq platform offers a feasible path for broad implementation of PGx testing and optimization of individual drug treatments.

## Introduction

Genetic variation is a major factor influencing the wide interindividual variability in pharmacological responses, contributing significantly to differences in systemic drug exposure, safety, and efficacy [1]. Not accounting for this genetic variation can therefore lead to severe adverse reactions or a loss of efficacy, due to inappropriate drug choice and/or dosing. For instance, the enzymatic product of the *CYP2C9* gene is involved in metabolism of various therapeutic drugs including anticonvulsants (e.g. phenytoin), anticoagulants (e.g. warfarin), and antidiabetic agents (e.g. tolbutamide) [2, 3]. Multiple null alleles of *CYP2C9* have been identified that greatly diminish the metabolism rate of aforementioned drugs by disrupting enzyme synthesis or function (i.e. hydroxylation). Individuals carrying two null alleles are called *CYP2C9* poor metabolizers and are at risk of uncontrollable bleeding if prescribed the average dose of warfarin [4]. It has been estimated that more than 90% of the United States (US) population has at least one clinically actionable pharmacogenetic (PGx) variant that affects their response to medication [5].

These pharmacological responses may be explained or even predicted using PGx tests that identify variant alleles of genes known to affect drug absorption, distribution, metabolism, and excretion (ADME) or the target of drug action. As of October 21, 2021, there are 442 gene/drug pairs (e.g. *CYP2D6*/codeine) described by the Clinical Pharmacogenetics Implementation Consortium (CPIC) with accompanying levels of evidence for changing drug choice and dosing decisions [6]. The assigned levels (A, B, C, and D) are subject to change, and only level A and B gene/drug pairs have sufficient evidence for at least one prescribing action to be recommended, and only level A gene/drug pairs have the preponderance of evidence that is highly or moderately in favour of changing prescribing. The US Food and Drug Administration (FDA) provides additional guidance by requiring applicable PGx test information be included in the drug labeling.

Although PGx field is often considered a low-hanging fruit for precision medicine, broad implementation of PGx testing has faced several obstacles, and only a few PGx tests are currently routinely used in the clinic [7]. One major barrier has been the sheer complexity of many pharmacogenes, necessitating a large number of genetic variants to be tested in order to provide precise predictions of enzymatic activity [8]. For example, according to the *CYP2D6* gene page of the Pharmacogene Variation Consortium (PharmVar) (accessed on October 31, 2021) [9], there are currently a total of 149 haplotype patterns (star alleles) defined by single-nucleotide variants (SNVs), small insertion-deletions (indels), and/or large structural variants (SVs). These alleles encode CYP2D6 enzymes with normal, decreased, increased, or no function, which are informative for determining final PGx phenotypes ranging from ultrarapid to poor metabolizer [10]. The frequency of star alleles and phenotypes can vary considerably across different populations [11]. Importantly, many of the *CYP2D6* alleles include SVs such as gene deletions, duplications, and hybrids, which are notoriously difficult to detect due to the high sequence homology between *CYP2D6* and its pseudogene *CYP2D7* [12]. Thus, several orthogonal genotyping methods including TaqMan assays, long-range polymerase chain reaction (PCR), quantitative multiplex PCR, High Resolution Melt analysis, and Sanger sequencing are required to accurately call all SVs in *CYP2D6* [13].

As next-generation sequencing (NGS) costs continue to decline, and the routine identification of rare variants becomes an imperative, sequencing-based association analysis is developing as a widely applied tool in human genetic research through whole exome sequencing and whole genome sequencing (WGS) as well as the application of targeted sequencing panels. PGx research is not an exception to this towering trend, and an increasing number of studies have already demonstrated the powerful utility of NGS data coupled with relevant bioinformatics tools for accurate PGx genotyping [14–19]. In particular, the targeted sequencing approach has recently gained much attention from the PGx community for its scalability and cost effectiveness–for example, providing an opportunity to characterize individual genetic variation in *CYP2A6* and *CYP2B6* for an underrepresented population [20] and serving as a big data platform for artificial intelligence-based prediction of *CYP2D6* haplotype function [21]. However, most existing panels often only focus on the detection of SNVs and indels, highlighting the need for probe design that can support comprehensive SV testing as well.

The Genetic Testing Reference Materials Coordination Program (GeT-RM) has been established by the Centers for Disease Control and Prevention to systematically catalogue genomic DNA reference materials to help the genetic testing community acquire characterized reference materials. These include DNA from Coriell cell line samples which have been genotyped using several commercial and laboratory-developed PGx testing assays [22–24]. Additionally, GeT-RM has made WGS data for 70 of the reference samples publicly available; these samples are ideal for PGx testing because they represent a genetically diverse set of samples from 11 distinct populations (e.g. African ancestry, Yoruba, Han Chinese, Japanese, Utah/Mormon, Finnish) in which we would likely encounter a wide range of PGx variants, including complex SVs.

Here, we present an overview of the design, quality control, and testing of ClinPharmSeq, a new targeted sequencing panel of 59 genes with associations to clinical drug response phenotypes. We describe our main design strategies to balance between low cost, high throughput, and deep coverage and to overcome some of the challenges related to accurate detection of complex PGx polymorphism including SVs. For quality control, we report the results of depth of coverage analyses to ensure that the panel can reliably generate high and uniform sequence coverage across all samples. Finally, we have tested ClinPharmSeq by performing extensive comparison analyses with the WGS data from GeT-RM as well as previously published genotype data.

## Materials and methods

### ClinPharmSeq design

**Fig 1:**
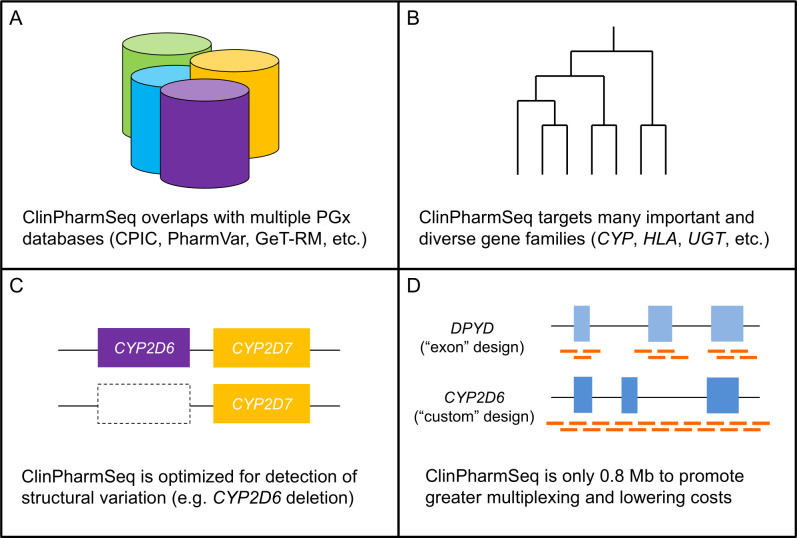
Major design strategies used to construct ClinPharmSeq.

The design strategies used to construct the first version of ClinPharmSeq (v1.0) are illustrated in Fig 1. We developed the panel using the SureSelect Custom DNA Target Enrichment Probes from Agilent Technologies, Inc. (Santa Clara, CA, USA) with its overall probe size being only 0.8 mega base pairs (Mb). One major consideration when building ClinPharmSeq was selection of PGx genes, as one of the design criteria was to create a panel that could be used for broad implementation of PGx testing while being cost competitive compared to other genotyping platforms such as SNP arrays. We searched candidate genes through various databases and resources including, but not limited to, CPIC, FDA, PharmVar, GeT-RM, the Pharmacogenomics Knowledge Base (PharmGKB) [25], the PGRNseq panel [26], the Stargazer program [17, 18], and the Database of Genomic Variants (DGV) [27].

As shown in Table 1, we selected a total of 59 PGx genes based on 1) their significant role in metabolism of, or response to, one or several drugs according to CPIC and FDA, 2) the availability of allelic variation catalogue with PharmVar, PharmGKB, and DGV, 3) the presence of genotyping reference materials via GeT-RM, and 4) their overlap with other existing tools such as PGRNseq and Stargazer. For example, as of October 21, 2021, the selected genes cover 60.0% (265/442) of all the CPIC gene-drug pairs, 88.6% (70/79) of the pairs with level A, and 100% (172/172) of the pairs with FDA label. Additionally, 36 of the 59 genes belong to one of the seven diverse enzyme families: Cytochrome B5 reductase (CYB5), Cytochrome P450 (CYP), Glutathione S-transferase (GST), Human leukocyte antigen (HLA), Arylamine N-acetyltransferase (NAT), solute carrier (SLC), and UDP Glucuronosyltransferase (UGT). These families represent various enzymatic functions including ADME and drug-induced disease.

**Table 1:** Summary of 59 pharmacogenes targeted by ClinPharmSeq and their mean sequencing coverage for Set 1 and Set 2.

No.	Gene	Chrom.	Function	Probe^a^	Set 1	Set 2	Design^b^	CPIC^c^	CPIC-A^d^	FDA^e^
1	*BCHE*	chr3	Other	7.7	345.4	311.7	Exon	2	0	1
2	*CACNA1S*	chr1	Target	16	177.3	151.6	Exon	7	7	5
3	*CFTR*	chr7	Target	16.7	352	314.8	Exon	2	1	1
4	*CYB5R1*	chr1	Metabolism	8.8	332.2	286.1	Exon	1	0	1
5	*CYB5R2*	chr11	Metabolism	11.1	272.9	232.7	Exon	1	0	1
6	*CYB5R3*	chr22	Metabolism	9.8	155.4	131.5	Exon	1	0	1
7	*CYB5R4*	chr6	Metabolism	13.1	315.8	283	Exon	1	0	1
8	*CYP1A2*	chr15	Metabolism	6.1	238.9	201.4	Exon	0	0	0
9	*CYP2A6*	chr19	Metabolism	49.3	352.5	301.2	Custom	0	0	0
10	*CYP2B6*	chr19	Metabolism	46.4	324	281.1	Custom	4	1	1
11	*CYP2C8*	chr10	Metabolism	7.9	389.3	344	Exon	3	0	0
12	*CYP2C9*	chr10	Metabolism	7.4	415	366.9	Exon	24	11	12
13	*CYP2C19*	chr10	Metabolism	9.1	403.3	355	Exon	21	8	16
14	*CYP2D6*	chr22	Metabolism	36.6	259.8	231.5	Custom	73	16	59
15	*CYP2E1*	chr10	Metabolism	29.7	318.2	266.5	Custom	0	0	0
16	*CYP2J2*	chr1	Metabolism	8	348.2	301.7	Exon	0	0	0
17	*CYP3A4*	chr7	Metabolism	8.6	355.1	313.9	Exon	1	0	0
18	*CYP3A5*	chr7	Metabolism	11	377.3	335.5	Exon	4	1	0
19	*CYP4F2*	chr19	Metabolism	6.8	266	229.2	Exon	3	1	0
20	*DPYD*	chr1	Excretion	14.1	328.1	293.2	Exon	3	2	2
21	*EGFR*	chr7	Target	19.4	326.5	285.3	Exon	0	0	0
22	*F5*	chr1	Other	16.5	383.6	336.8	Exon	2	0	1
23	*G6PD*	chrX	Drug-induced disease	8.4	116.5	111.1	Exon	34	3	23
24	*GBA*	chr1	Other	9	249	220.3	Exon	1	1	1
25	*GSTM1*	chr1	Metabolism	11	146.8	108.5	Custom	2	0	0
26	*GSTP1*	chr11	Metabolism	5.2	199.3	171.2	Exon	4	0	0
27	*GSTT1*	chr22	Metabolism	11.6	134.8	89.9	Custom	0	0	0
28	*HLA-A*	chr6	Toxicity	9.3	294.6	262	Custom	3	1	1
29	*HLA-B*	chr6	Toxicity	9.4	318.4	271.7	Custom	13	6	7
30	*HLA-C*	chr6	Toxicity	9.4	334.6	284.5	Custom	2	0	0
31	*HLA-DPB1*	chr6	Toxicity	16.4	332.2	286.3	Custom	1	0	0
32	*HLA-DQA1*	chr6	Toxicity	12	309.5	251	Custom	1	0	1
33	*HLA-DRB1*	chr6	Toxicity	19.1	333.6	293.6	Custom	2	0	1
34	*HPRT1*	chrX	Other	6.7	229.3	223.5	Exon	1	0	1
35	*IFNL3*	chr19	Other	5.1	240.1	208.6	Exon	2	2	1
36	*LDLR*	chr19	Target	11.9	235.8	200.4	Exon	1	0	1
37	*NAGS*	chr17	Other	6.4	164.5	140.8	Exon	2	0	1
38	*NAT1*	chr8	Metabolism/excretion	8	355.5	311	Exon	0	0	0
39	*NAT2*	chr8	Metabolism/excretion	5.5	390.4	347.8	Exon	7	1	5
40	*NUDT15*	chr13	Metabolism	6.2	362.3	320.4	Exon	3	3	3
41	*OTC*	chrX	Other	6.4	271.2	263.2	Exon	1	0	1
42	*POLG*	chr15	Other	11.9	242.4	206.4	Exon	2	2	2
43	*POR*	chr7	Drug-induced disease	12.4	157.5	136.7	Exon	0	0	0
44	*PROC*	chr2	Other	6.8	229.1	196.7	Exon	1	0	1
45	*PROS1*	chr3	Other	10.1	323.1	287.9	Exon	1	0	1
46	*RYR1*	chr19	Drug-induced disease	32.9	152.5	130.2	Exon	7	7	5
47	*SERPINC1*	chr1	Other	6.5	337.7	299.8	Exon	1	0	1
48	*SLC15A2*	chr3	Excretion	12.8	383	338.1	Exon	0	0	0
49	*SLC22A2*	chr6	Excretion	51.5	325.6	286.4	Custom	0	0	0
50	*SLCO1B1*	chr12	Absorption	8.8	328.4	300.4	Exon	5	1	3
51	*SLCO2B1*	chr11	Absorption	15	268.5	231.5	Exon	0	0	0
52	*TPMT*	chr6	Metabolism	7.8	268	235.3	Exon	3	3	3
53	*UGT1A1*	chr2	Excretion	12.8	371.8	323.3	Exon	7	2	6
54	*UGT1A4*	chr2	Excretion	7.7	350.5	302.6	Exon	1	0	0
55	*UGT2B7*	chr4	Excretion	6.8	327	297.7	Exon	0	0	0
56	*UGT2B15*	chr4	Excretion	30.6	331.7	299.3	Custom	1	0	0
57	*UGT2B17*	chr4	Excretion	30.3	177.2	161.5	Custom	0	0	0
58	*VDR*	chr12	Absorption	10.6	288	247	Exon	2	0	0
59	*VKORC1*	chr16	Target	4.9	246.8	208	Exon	1	1	1

Abbreviations: No., number; Chrom., chromosome; CPIC, Clinical Pharmacogenetics Implementation Consortium; FDA, Food and Drug Administration.

Samples were sequenced in two separate runs (N = 32 for Set 1 and N = 32 for Set 2).

^a^Total length of targeted regions in kilo base pairs.

^b^Panel design strategy used to probe each gene. The ‘exon’ design includes probes for targeting exons and upstream/downstream regions of a gene. The ‘custom’ design was used to capture genes with structural variation and/or complex polymorphism.

^c^Total number of CPIC gene-drug pairs, as of October 21, 2021.

^d^Total number of CPIC gene-drug pairs with level A, as of October 21, 2021.

^e^Total number of CPIC gene-drug pairs with FDA label, as of October 21, 2021.

Among the targeted genes, several are known to have complex patterns of nucleotide polymorphism (e.g. *HLA-A*) and/or SVs (e.g. *CYP2D6*). To comprehensively assess the wide variety of allelic variation in these genes, probes were arranged so that the entire gene locus is captured including the 3 kilo base pairs (kb) upstream/downstream regions. In addition, for genes that are known to have SVs with their pseudogene (e.g. *CYP2D6/CYP2D7* hybrids), probes were extended to cover the pseudogenes as well. This kind of ‘custom’ design was used for 15 out of the 59 genes (Table 1). For the rest, probes were placed mostly for the exons (i.e. the ‘exon’ design) plus the 50 bp padding sequence in intron/exon boundaries to identify splice variants and the 3 kb upstream/downstream regions to capture regulatory elements such as promoter. Of note, three of the selected genes (*EGFR*, *RYR1*, and *VDR*) are a good control locus for read depth normalization during copy number analysis, which can be useful for SV detection, because they are large in length (188.3, 153.9, and 63.5 kb, respectively) and reported to exhibit low rates of whole gene deletion and/or duplication [17].

The use of the ‘exon’ and ‘custom’ designs also helped reduce the panel’s overall size tremendously, which is essential for promoting greater multiplexing and lowering costs. For example, one of the genes targeted by ClinPharmSeq is *DPYD* and its length alone is comparable to the panel itself (0.8 Mb). However, with the ‘exon’ design the gene’s probe size is reduced to just 14.1 kb (~98% reduction), which is a logical choice given that all 458 alleles except one (HapB3) in PharmVar’s *DPYD* gene page (accessed on October 31, 2021) are defined with single coding variant–HapB3 is defined with one intronic variant and one synonymous variant. Another good example is *CYP2B6* where the ‘custom’ design shrinks the gene’s probe size from 107.1 to 46.4 kb while ensuring that SVs are still accurately detected (see below for the detection of CYP2B6*29 which is a *CYP2B7/CYP2B6* hybrid) (S1 Fig).

### ClinPharmSeq sequencing

To test the genotyping ability and investigate the potential limitations of ClinPharmSeq, we sequenced a total of 64 Coriell DNA samples of diverse ancestry (S1 Table). Libraries were prepared for sequencing using the SureSelect^XT^ Library Prep Kit from Agilent Technologies, Inc. per the manufacturer’s recommendations with minor modification. Sequencing was performed with the NextSeq 500 System from Illumina, Inc. (San Diego, CA, USA) using 150 base pairs (bp) paired-end reads. Raw sequencing data were produced in the Binary Base Call (BCL) file format, which were then converted to individual FASTQ files by demultiplexing with the bcl2fastq program (v2.19.0). We aligned sequence reads in the FASTQ files to the Human Genome version 19 (hg19) reference genome using the ‘ngs-fq2bam’ command from the fuc package (v0.26.0, https://github.com/sbslee/fuc). The command generates a pipeline for automatically converting FASTQ files to ‘analysis-ready’ Binary Alignment Map (BAM) files by combining various commands from the BWA [28], SAMtools [29], and Genome Analysis Toolkit (GATK) [30] programs. Finally, we used the ‘bedcov’ command from SAMtools (v1.13) to calculate average coverage across the target space.

### Variant call comparison

To measure the variant calling performance of ClinPharmSeq, we compared its calls with that of WGS data from GeT-RM. The WGS data were downloaded via the European Nucleotide Archive (study accession: PRJEB19931) in the BAM file format, which contained 150 bp paired-end reads aligned to hg19 with the ISAAC program [31]. We identified SNVs and indels from BAM files to create a multi-sample Variant Call Format (VCF) file using the ‘ngs-hc’ command from fuc; the command combines a series of GATK tools including HaplotypeCaller with predetermined parameters into an automated pipeline for convenience and reproducibility. A Browser Extensible Data (BED) file describing the probed sites of ClinPharmSeq was given to the command to only output variants in the regions of interest. We then used the Python API of the pyvcf submodule from fuc to 1) merge two VCF files from the ClinPharmSeq and WGS data with the ‘pyvcf.merge’ method, 2) remove any multiallelic sites from the merged VCF file with the ‘pyvcf.VcfFrame.filter_multialt’ method, and 3) perform the comparison with the ‘pyvcf.VcfFrame.calculate_concordance’ method.

### Star allele identification

We assessed star alleles in 27 PGx genes using NGS data with the PyPGx package (v0.7.0, https://github.com/sbslee/pypgx) which implements a modified version of the Stargazer genotyping pipeline [17, 18]. Briefly, the PyPGx pipeline works by first haplotype phasing SNVs and indels present in the target gene using the Beagle program [32] with the 1000 Genomes Project haplotype reference panel [33]. Phased variants are then matched to star alleles in the gene’s haplotype translation table. Next, per-base read depth in the target gene is converted to copy number by intra-sample normalization using a control gene. In the case of targeted sequencing data (e.g. ClinPharmSeq), the inter-sample normalization is additionally performed to consider the heterogeneity in total coverage across the samples. For this normalization step, users can choose to use summary statistics across all samples or known samples without SV in the target gene–the latter is recommended if the gene is known have an extremely high rate of gene deletion polymorphism, such as *GSTT1*. From copy number data, SVs are detected using a support vector machine-based multiclass classifier using the one-vs-rest strategy. SV results are incorporated to inform the final star allele assignment. Output data of PyPGx include individual diplotype calls and plots of copy number and allele fraction profile to visually inspect SV calls.

We ran the ‘run-ngs-pipeline’ command from PyPGx for each target gene with three input files: 1) a multi-sample VCF file, 2) a depth of coverage file, and 3) a control statistics file. The latter two were created from BAM files using the ‘prepare-depth-of-coverage’ and ‘compute-control-statistics’ commands from PyPGx, respectively. The analyses shown in the results section were all performed using the *VDR* gene as the control locus. For the ClinPharmSeq data we used known samples without SV for the genes *GSTM1*, *GSTT1*, and *UGT2B17* during the inter-sample normalization step of copy number analysis.

### Comparison of star allele diplotypes

We compared diplotype calls from PyPGx to previously published data. Of the 27 PGx genes assessed, *CYP2D6* was compared with diplotype data from [23] where samples were extensively characterized using multiple standard methods, including allele-specific PCR, long-range PCR, SNP arrays, TaqMan real-time PCR assays, droplet digital PCR, Sanger sequencing, and long-read sequencing. For the four genes *CYP2C8*, *NAT1*, *SLC15A2*, and *VKORC1* we obtained diplotype data from [22] in which samples were genotyped using several commercial and laboratory-developed PGx testing assays (e.g., allele-specific PCR, molecular inversion probes, hybridization-based arrays, and TaqMan assays). For the remaining 22 genes, we utilized diplotype data from [18] where the identical WGS data from GeT-RM were analyzed using Stargazer. Of note, [18] reported that one of the samples, NA18540, has trisomy for multiple chromosomes, including chromosomes 4 and 7, affecting genotype calling in five genes (*CYP3A4*, *CYP3A5*, *UGT2B7*, *UGT2B15*, and *UGT2B17*); therefore, for each gene the duplicated allele was ignored because it is a cell line artifact rather than a result of natural PGx variation (e.g. a UGT2B15*2/*2/*4 call was treated as *2/*4).

For the purpose of this comparison, several allelic inconsistencies were also ignored because they were specific to the genotype calling algorithm (S4 Table). For example, the three alleles CYP2C8 *15, *16, and *17 were observed only from the WGS and ClinPharmSeq data, and not from the previous diplotypes, but these were not counted as discrepancy because they were recently added to PharmVar and did not exist at the time of testing by [22].

### Phenotype prediction

We predicted PGx phenotypes for nine genes that have a genotype-phenotype table from CPIC: *CYP2B6*, *CYP2C19*, *CYP2C9*, *CYP2D6*, *CYP3A5*, *DPYD*, *SLCO1B1*, *TPMT*, and *UGT1A1*. Specifically, we used the ‘pypgx.predict_phenotype’ method from PyPGx to translate individual diplotype calls to predicted phenotypes. The *SLCO1B1* gene produced transporter function phenotype ranging from poor to increased function, while the rest produced metabolizer status phenotype ranging from poor to ultrarapid metabolizer. Additionally, the genes *CYP2C9*, *CYP2D6*, and *DPYD* utilized a standard unit of enzyme activity known as an activity score for final phenotype prediction [10]. For example, the fully functional reference CYP2D6*1 allele is assigned a value of 1, decreased-function alleles such as CYP2D6*9 and *17 receive a value of 0.5, and nonfunctional alleles including CYP2D6*4 and *5 have a value of 0. The sum of values assigned to both alleles constitutes the activity score of a diplotype. Consequently, subjects with CYP2D6*1/*1, *1/*4, and *4/*5 diplotypes have an activity score of 2 (normal metabolizer), 1 (intermediate metabolizer), and 0 (poor metabolizer), respectively. The other six genes used simple diplotype-to-phenotype mapping system (e.g. a CYP2B6*6/*8 diplotype is mapped to poor metabolizer).

## Results

### General platform performance

We sequenced 64 Coriell DNA samples of diverse ancestry with ClinPharmSeq to assess its utility as a PGx genotyping platform. The samples were evenly split into two sets, Set 1 and Set 2, to be sequenced with a 32-plex capture strategy, which led to average coverages of 292x and 255x across the target space, respectively (mean = 274x). ClinPharmSeq also consistently achieved high uniformity in depth of coverage with 95.2% and 94.4% (mean = 94.8%) of the covered bases showing a depth of 30x or above (S1 Fig), suggesting that it can reliably generate deep sequencing data while maintaining the high throughput required for studies of large sample sizes. At the single-gene level, ClinPharmSeq generated deep coverage data for every captured gene regardless of whether the ‘custom’ or ‘exon’ design was used (Table 1). Of note, the genes *GSTM1*, *G6PD*, and *GSTT1* produced the lowest average coverages of 128x, 114x, and 112x, respectively. However, this was expected because *G6PD* is located on the X chromosome and because both *GSTT1* and *GSTM1* are known to have an extremely high rate of gene deletion polymorphism in the population (see below for the detection of whole gene deletions in *GSTT1* and *GSTM1*).

### Variant calling concordance with WGS data

Although ClinPharmSeq generated high-coverage data, many of the targeted genes could still be prone to erroneous variant calls. This is because those genes could potentially have inefficient, or even inappropriate, capture performance and misalignment of sequence reads due to sequence homology with other gene family members and/or the presence of SVs. Therefore, we set out to test the accuracy of ClinPharmSeq on variant identification by comparing its genotype calls with that of WGS data from GeT-RM. For this comparison we only considered biallelic sites that are located within the regions targeted by ClinPharmSeq.

**Fig 2:**
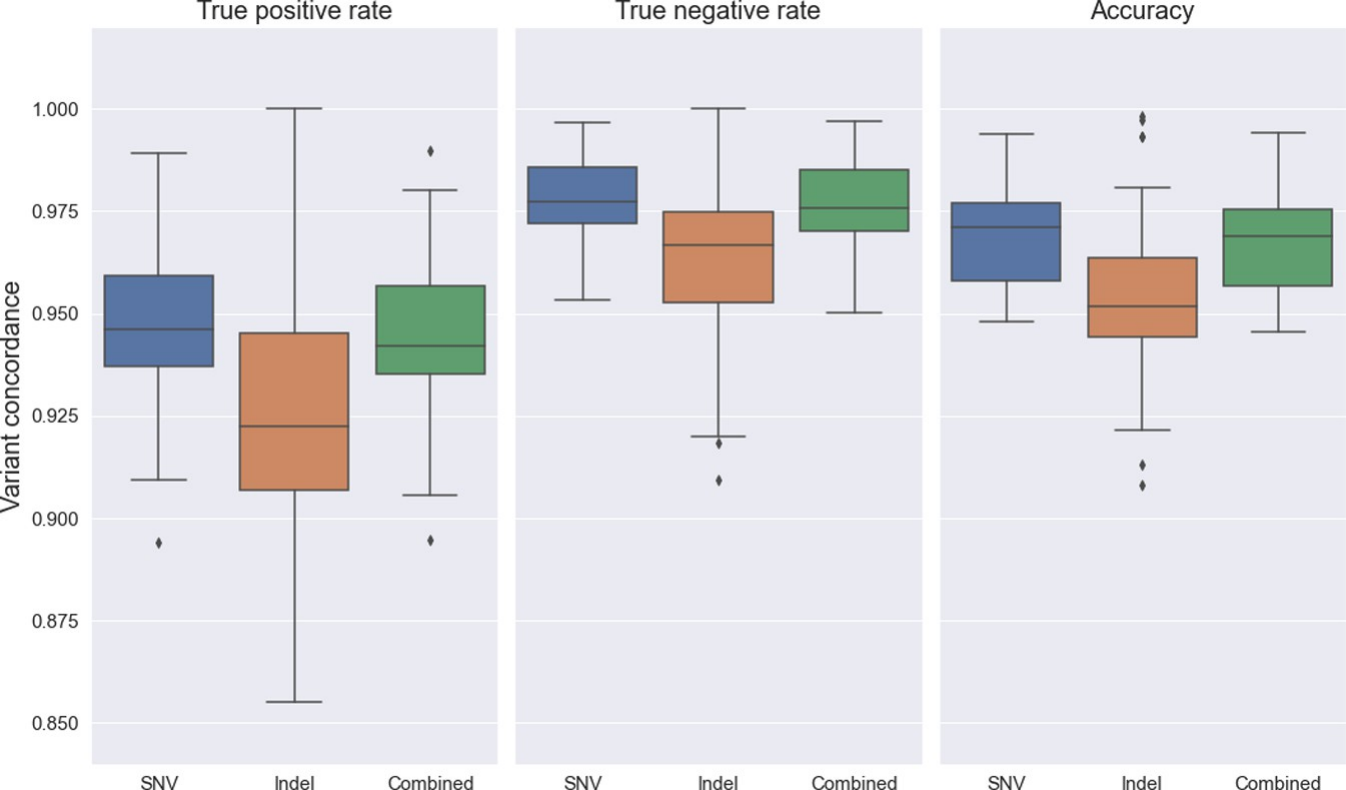
Variant call concordance between WGS and ClinPharmSeq.

A total of 12,415 variants were compared and the mean per-sample concordance was 96.0%. As shown in Fig 2, we obtained high concordance rates even when we divided the variants into SNVs and indels and when we considered false positives and false negatives separately, indicating that most ClinPharmSeq-derived genotypes are accurate. However, one of the samples, NA18973, showed a notably low accuracy of 76.7% and was later also found to have a completely different PGx variation pattern including SVs (see below). Further inspection revealed that, due to a clerical error, this sample was never sequenced with ClinPharmSeq–but instead NA18972 was which is not part of the 70 GeT-RM samples–and thus it was excluded from all comparative analyses. After exclusion of this sample the mean per-sample concordance was increased to 96.3%.

### Comparison of star allele calling with WGS and previous publications

To evaluate the performance of ClinPharmSeq on star allele calling, we used the PyPGx program to identify PGx diplotypes in 27 genes for the 63 ClinPharmSeq samples (N = 1,701 diplotypes) and the 70 WGS samples (N = 1,890 diplotypes) (S2 Table). From the two sets of diplotype calls we found a total of 185 unique star alleles (Table 2). The diplotype sets were compared to each other as well as to previous data from three prior publications (S2 Table). As mentioned above, ClinPharmSeq data from the sample NA18973 was excluded because it was an incorrect sample and showed a completely different variant landscape including SVs in *CYP2D6* (S4 Fig). Additionally, 14 alleles were excluded from the comparisons because they were not reported from the previous publications (Table 2).

**Table 2:** Star alleles assessed by PyPGx’s analysis of whole genome sequencing and ClinPharmSeq data.

No.	Gene	Reference	Alleles Reported in This Study	Alleles Previously Not Reported
1	*CYP1A2*	*1A	*1A, *1F, *1L	*None*
2	*CYP2A6*	*1	*1, *1x2 (dup), *2, *4 (del), *7, *9, *15, *17, *18, *20, *21, *22, *23, *24, *25	*None*
3	*CYP2B6*	*1	*1, *2, *4, *5, *6, *9, *15, *17, *18, *20, *22, *23, *29 (hyb)	*None*
4	*CYP2C8*	*1	*1, *2, *3, *4, *15, *16, *17	*15, *16, *17
5	*CYP2C9*	*1	*1, *2, *3, *5, *6, *8, *9, *11, *61	*61
6	*CYP2C19*	*1	*1, *2, *3, *4, *6, *8, *13, *15, *17, *35, *39	*39
7	*CYP2D6*	*1	*1, *2, *2x2 (dup), *4, *4x2 (dup), *5 (del), *6, *9, *10, *14, *15, *17, *21, *29, *35, *36+*10 (hyb), *36x2+*10 (hyb), *40, *41, *45, *46, *68+*4 (hyb), *71, *106	*106
8	*CYP2E1*	*1	*1, *5, *7, *7x2 (dup), *7x3 (dup), *S1 (dup)	*None*
9	*CYP3A4*	*1	*1, *2, *3, *15, *16, *22	*None*
10	*CYP3A5*	*1	*1, *3, *6, *7	*None*
11	*CYP4F2*	*1	*1, *2, *3	*None*
12	*DPYD*	*Reference*	*Reference*, *c*.85T>C (*9A), *c*.*496A>G*, *c*.*557A>G*, *c*.*1218G>A*, *c*.*1349C>T*, *c*.1601G>A (*4), *c*.1627A>G (*5), *c*.*1682G>T*, *c*.*1896T>C*, *c*.2194G>A (*6), *c*.*2846A>T*	*c*.*1349C>T*, *c*.*1682G>T*, *c*.*2846A>T*
13	*GSTM1*	*A	*A, *B, *Ax2 (dup), *0 (del)	*None*
14	*GSTP1*	*A	*A, *B, *C	*None*
15	*GSTT1*	*A	*A, *0 (del)	*None*
16	*NAT1*	*4	*4, *11, *14, *17	*None*
17	*NAT2*	*4	*4, *5, *6, *7, *12, *13, *14, *24	*24
18	*SLC15A2*	*1	*1, *2	*None*
19	*SLC22A2*	*1	*1, *2, *3, *4, *6, *7, *S1 (del), *S2 (del)	*None*
20	*SLCO1B1*	*1A	*1A, *1B, *5, *14, *15, *17, *20, *21, *24, *27, *30, *31, *32, *S1, *S2	*20, *32
21	*SLCO2B1*	*1	*1, *S1, *S464F	*None*
22	*TPMT*	*1	*1, *3C, *8, *16	*None*
23	*UGT1A1*	*1	*1, *6, *28, *36, *80, *80+*28, *80+*37	*80+*27, *80+*37
24	*UGT2B7*	*1	*1, *2, *3	*None*
25	*UGT2B15*	*1	*1, *2, *4, *5, *S1 (del)	*None*
26	*UGT2B17*	*1	*1, *2 (del)	*None*
27	*VKORC1*	*Reference*	*Reference*, *rs9923231*	*None*

Structural variant-defined alleles are indicated by ‘del’ (deletion), ‘dup’ (duplication), and ‘hyb’ (hybrid).

**Fig 3:**
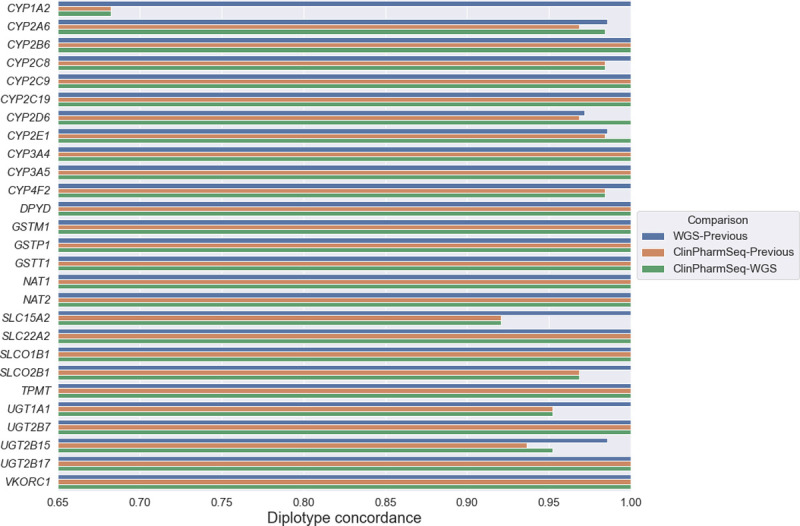
Concordance of diplotype calls for 27 pharmacogenes between WGS, ClinPharmSeq, and previous studies.

ClinPharmSeq showed diplotype concordances of 97.6% (1,660/1,701) and 97.9% (1,665/1,701) with the previous publications and WGS, respectively, while the latter two had a concordance rate of 99.7% (1,885/1,890) to each other (Fig 3). All the 36 inconsistencies between ClinPharmSeq and WGS were encapsulated in the 41 inconsistencies between ClinPharmSeq and the previous publications (S3 Table). Among those inconsistencies, almost half (N = 19) were caused by ClinPharmSeq entirely missing the CYP1A2*1L allele because one of its variants (15-75038220-G-A) is in a region that is not targeted by the current version of ClinPharmSeq. Additionally, a total of 12 inconsistencies were caused by incorrect haplotype phasing by PyPGx where, for example, the UGT2B15*4 variants (4-69536084-A-C and 4-69512847-T-G) were incorrectly phased in trans for four samples. Six of the inconsistencies were caused by failed variant calling in which, for instance, the UGT1A1*28 variant (2-234668879-C-CAT) was called as heterozygous instead of homozygous for three samples.

**Fig 4:**
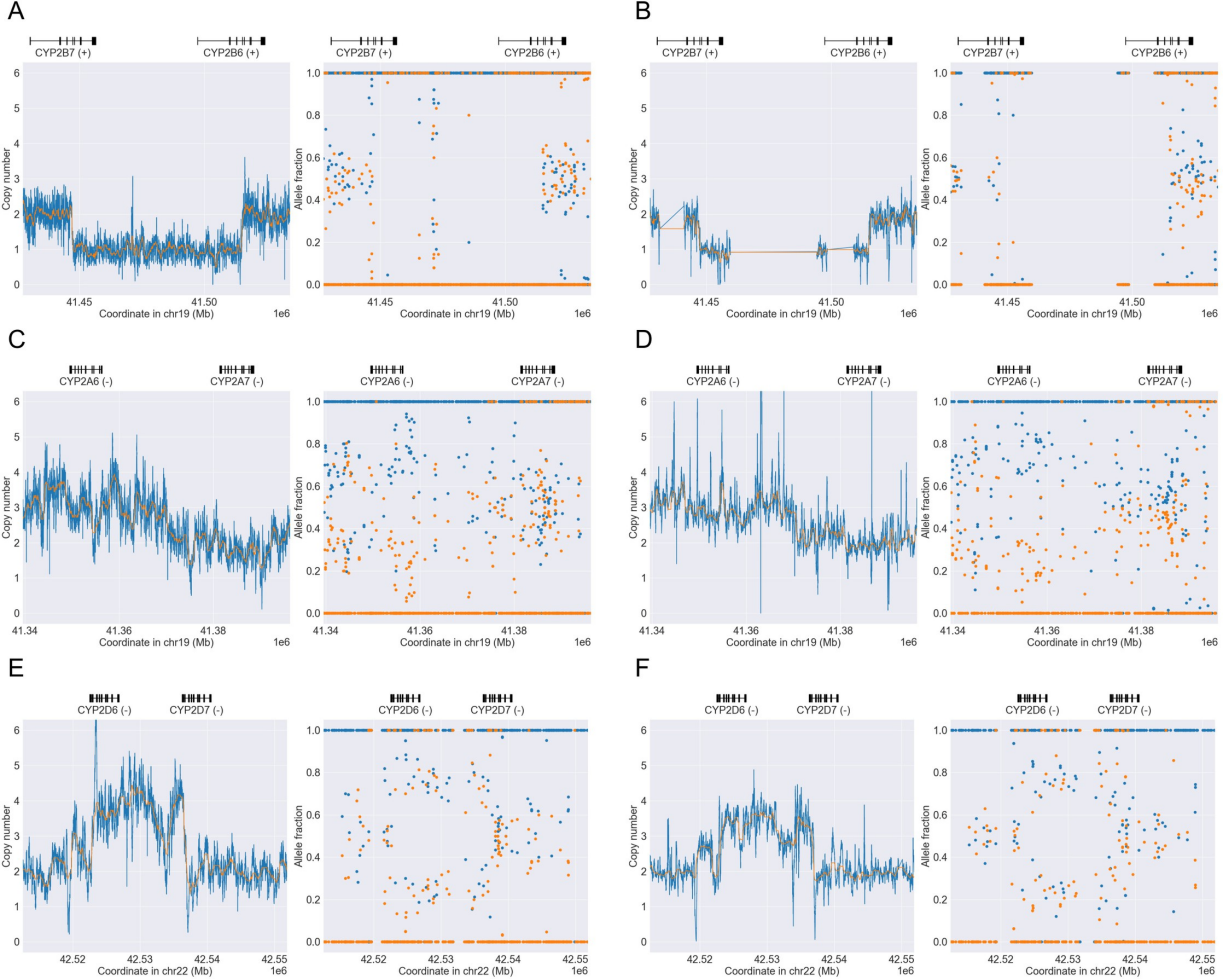
Examples of SVs detected with WGS and ClinPharmSeq. WGS data are shown in the left panels (A, C, and E) while ClinPharmSeq data are shown in the right panels (B, C, and D). Each panel contains a copy number profile and an allele fraction profile created by PyPGx. (A-B) *CYP2B7/CYP2B6* hybrid in African sample NA19178 with a CYP2B6*6/*29 diplotype. (C-D) Gene duplication in African sample NA18861 with a CYP2A6*1x2/*25 diplotype. (E-F) Complex *CYP2D6/CYP2D7* hybrid in East Asian sample NA18526 with a CYP2D6*1/*36x2+*10 diplotype.

From the ClinPharmSeq and WGS data we identified a total of 19 SV-carrying star alleles in nine targeted genes (Table 2). The alleles consisted of gene deletions (e.g. GSTT1*0), duplications (e.g. CYP2A6*1x2), multiplications (e.g. CYP2E1*7x3), and hybrids (e.g. CYP2D6*36+*10); these were collectively found in 10.4% (177/1,701) and 10.2% (192/1,890) of the diplotypes examined, respectively. As expected, a large proportion of the diplotypes from the genes *CYP2A6*, *GSTM1*, *GSTT1*, and *UGT2B17* had whole gene deletions, both heterozygous and homozygous (e.g. UGT2B17*1/*2 and *2/*2). For example, we found the gene deletion alleles GSTM1*0 and GSTT1*0 in 76.2 (48/63) and 77.8% (49/63) of the samples with ClinPharmSeq and 75.7 (53/70) and 77.1% (54/70) of the samples with WGS, respectively. Importantly, ClinPharmSeq and WGS showed a perfect concordance on all the SV alleles, indicating that ClinPharmSeq can reliably detect SVs despite it being a targeted capture panel (S2 Table). Three representative examples of SV detection (CYP2B6*29, CYP2A6*1x2, and CYP2D6*36x2+*10) by PyPGx using the ClinPharmSeq and WGS data are illustrated in Fig 4. Of note, ClinPharmSeq correctly identified the CYP2B6*29 allele, a *CYP2B7/CYP2B6* hybrid, even though the probes only covered less than half of the region.

**Fig 5:**
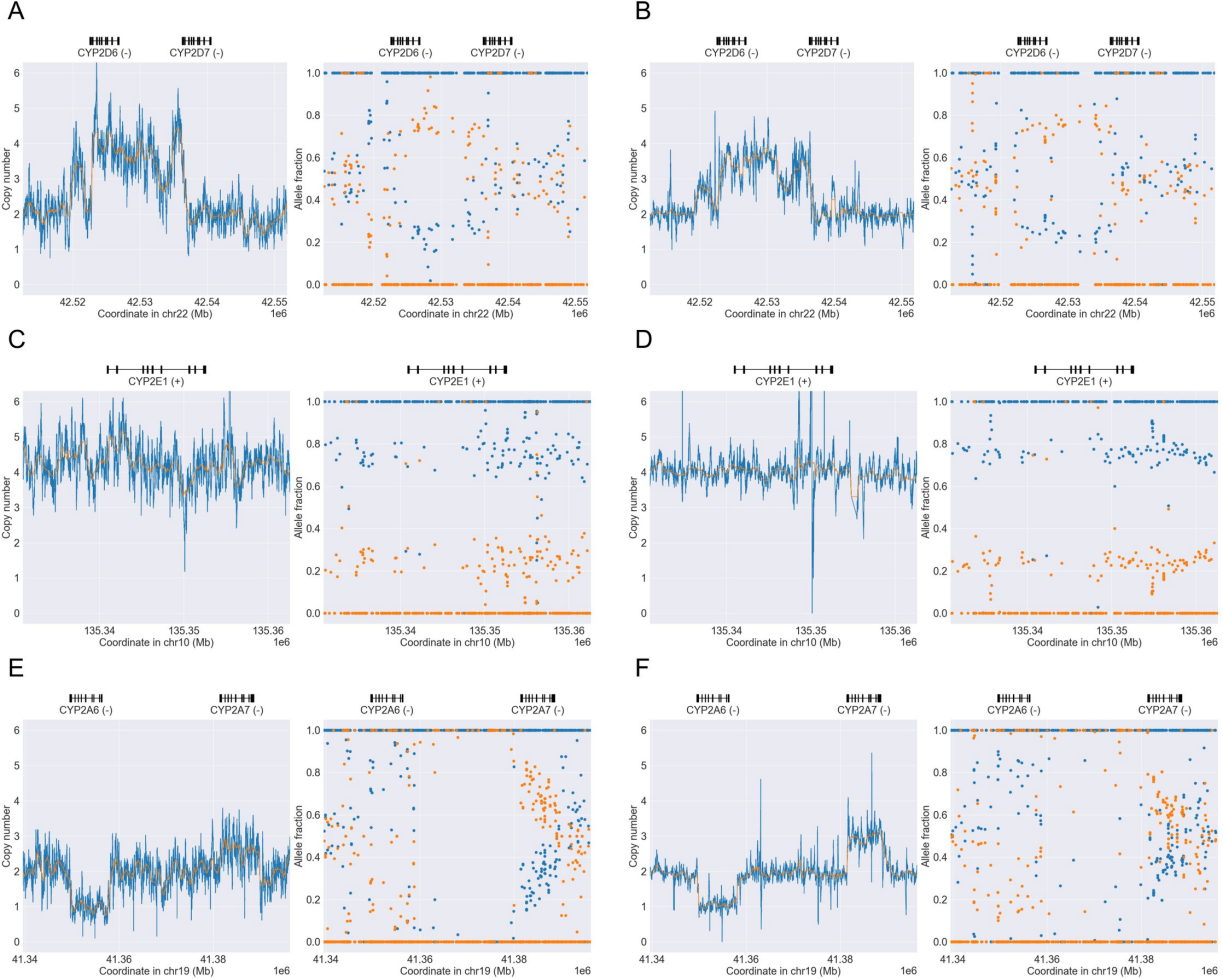
Examples of diplotype discrepancy caused by difference in SV interpretation between this study and previous publications. WGS data are shown in the left panels (A, C, and E) while ClinPharmSeq data are shown in the right panels (B, C, and D). Each panel contains a copy number profile and an allele fraction profile created by PyPGx. (A-B) East Asian sample NA18540 was previously identified to have three *CYP2D6* gene copies in total with a CYP2D6(*36+)10/*41 diplotype, but this study identified four gene copies with a CYP2D6*36x2+*10/*41 diplotype. (C-D) African sample NA19908 was previously suggested to have a CYP2E1*7x2/*7x2 diplotype (i.e. allele fraction ratio of 2:2), while this study found evidence of a CYP2E1*7/*7x3 diplotype (i.e. allele fraction ratio of 1:3). (E-F) East Asian sample HG00436 was previously genotyped to have a combination of one known SV (CYP2A6*4) and one novel SV (CYP2A6*1+*S6), while in this study PyPGx produced an ‘Indeterminate’ diplotype call because it could also be just one novel SV, which would be a more parsimonious explanation.

We discovered that four of the diplotypes from previous publications differed with the calls from both ClinPharmSeq and WGS because of SV detection and/or interpretation. In the first case, [23] genotyped the sample NA18540 for *CYP2D6* as (*36+)10/*41 with three gene copies in total, but NGS data suggested the presence of an extra gene copy with *36x2+*10/*41, indicated by the cluster of copy numbers at 4 and allelic depth ratio of 1:3 (Fig 5A and 5B). The second case also involved *CYP2D6* where the sample NA18565 was previously genotyped as *10/*36x2 while NGS data produced *10/*36+*10. The third inconsistency was the sample NA19908 with *CYP2E1* in which [18] presented *7x2/*7x2 but NGS data showed evidence of *7/*7x3 instead, indicated by allelic depth ratio of 1:3 instead of 2:2 (Fig 5C and 5D). The last difference relating to SV was the sample HG00436 with *CYP2A6* whereby [18] reported *4/*1+*S6 and NGS data produced an ‘Indeterminate’ call (Fig 5E and 5F). The former would mean the sample has a combination of one known SV (*4) and one novel SV (*1+*S6), but it could also be just one novel SV, which would be a more parsimonious explanation. These results suggest that ClinPharmSeq could be potentially used to validate previous calls and/or provide new insights for resolving complex SV events.

### PGx phenotype prediction comparison

**Fig 6:**
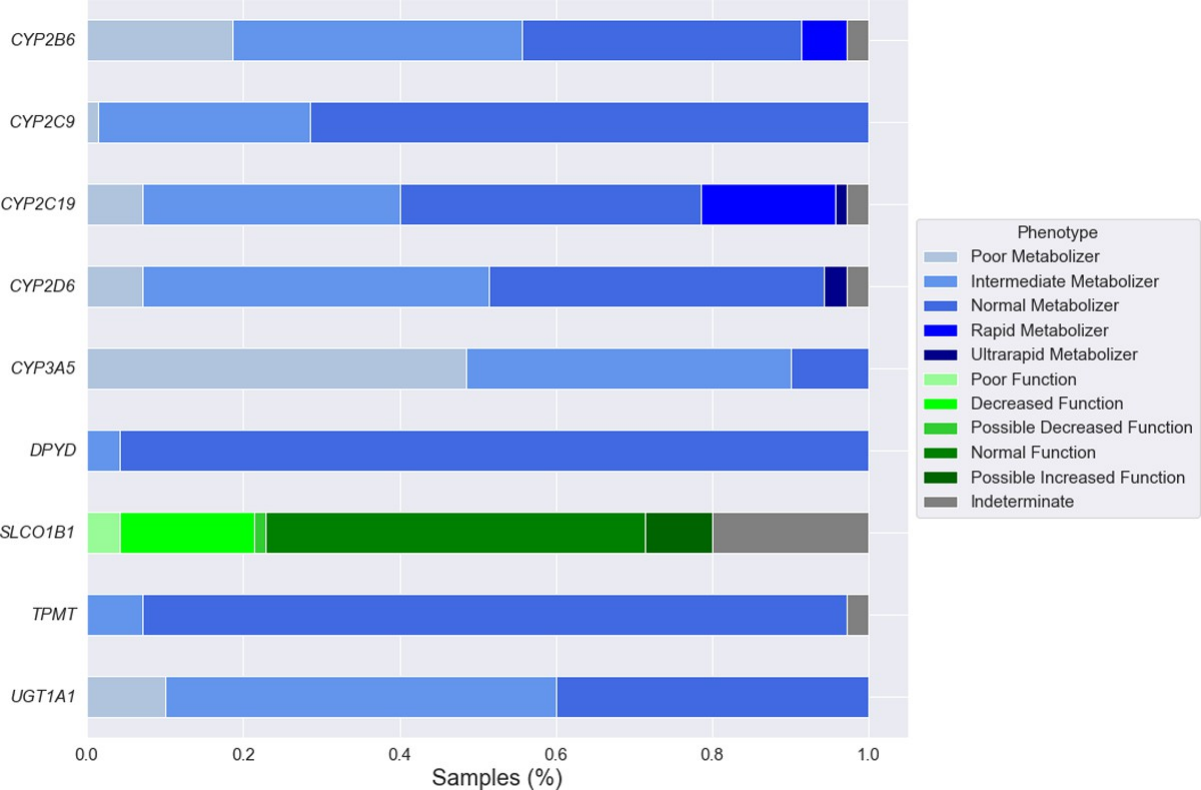
Distribution of predicted phenotypes for nine pharmacogenes with a CPIC genotype-phenotype table. WGS data (N = 70) is shown as a representative example.

In order to estimate the effect of diplotype inconsistency on final phenotype prediction, we assessed phenotype calls from PyPGx for nine targeted genes which have a CPIC genotype-phenotype table. The three pairwise comparisons yielded phenotype concordances of 98.1% (556/567) for ClinPharmSeq and the previous publications, 99.5% (564/567) for ClinPharmSeq and WGS, and 98.7% (622/630) for WGS and the previous publications (S3 Fig). The majority of inconsistencies (N = 8) stemmed from PyPGx newly calling a star allele with an unknown/uncertain function and changing the overall phenotype to ‘Indeterminate’ (S6 Table). For example, [23] genotyped the sample NA18519 as CYP2D6 *1/*29 which corresponds to a normal metabolizer, but PyPGx produced a *29/*106 diplotype which corresponds to an indeterminate phenotype because the enzymatic function of the *106 allele has not been determined yet. Overall, we saw a unique distribution of PGx phenotypes for each gene (Fig 6), with *CYP3A5* having the largest proportion of the poor metabolizer phenotype and *SLCO1B1* having the largest proportion of the indeterminate phenotype.

## Discussion

In this study, we developed ClinPharmSeq–a novel, SV-aware targeted sequencing panel of 59 clinically important pharmacogenes. To the best of our knowledge, this is one of the first SV-aware targeted panels to enable systematic identification of star alleles in multiple genes. To evaluate the performance of ClinPharmSeq, we utilized public WGS data from genotyping reference samples (N = 70) from GeT-RM as well as their previously published diplotype calls generated with multiple standard methods (e.g. allele-specific PCR, molecular inversion probes, hybridization-based arrays, and TaqMan assays). ClinPharmSeq consistently produced deep and uniform sequence coverage, and showed high accuracy in variant calling (i.e. SNVs and indels) compared to WGS. At the star allele level, for all 27 pharmacogenes tested, diplotype calls from ClinPharmSeq exhibited almost perfect concordance with that from WGS and the previous studies, which led to similar phenotype predictions as well. Altogether, these results demonstrate that ClinPharmSeq can accurately assess star alleles in the targeted genes and could be potentially used for clinical implementation of NGS-based PGx testing.

Our hybrid approach of the ‘exon’ and ‘custom’ designs greatly reduced the overall probe size of the panel (less than 1 Mb) while maintaining high sensitivity for detection of complex SVs. For example, from the ClinPharmSeq and WGS data we identified a total of 19 star alleles defined with SVs including gene deletions, duplications, and hybrids. When we visually inspected copy number and allele fraction plots from PyPGx, we noticed that the distribution of data points was much sharper with ClinPharmSeq than WGS, probably due to the deep coverage of ClinPharmSeq reducing the general variance of PyPGx estimates. Furthermore, we also found that our ClinPharmSeq data could be used to potentially change at least three previously published SV-carrying diplotype calls from already heavily characterized reference samples (e.g. CYP2E1*7x2/*7x2 to *7/*7x3).

In addition to SVs, the novel design of ClinPharmSeq also helped identify various types of small nucleotide variation than just biallelic coding variants. For example, we identified several PGx variants located in a regulatory element, such as CYP2A6*9 (19-41356379-A-C) which is a TATA box variant known to cause promoter defect and reduced gene expression [34]. ClinPharmSeq also proved to be useful for detecting complex multliallelic indels such as UGT1A1*28 (2-234668879-C-CAT), *36 (2-234668879-CAT-C), and *37 (2-234668879-C-CATAT), all of which are characterized by a varying number of TA repeats in the TATA sequence of the promoter [35]. Lastly, for the targeted genes with the ‘exon’ design addition of 50 bp padding sequence in intron/exon boundaries led to detection of splice variants, such as CYP3A5*6 (7-99262835-C-T) which is known to cause alternative splicing and protein truncation result [36].

For the 70 reference samples we found 14 new star alleles in eight genes (*CYP2C8*, *CYP2C9*, *CYP2C19*, *CYP2D6*, *DPYD*, *NAT2*, *SLCO1B1*, and *UGT1A1*) that were not reported by previous publications. These alleles were recently defined by PharmVar or CPIC and therefore did not exist at the time of testing by the respective studies, highlighting how rapidly the list of known haplotypes is growing for PGx genes and also how important the routine identification of rare variants is. Although enzymatic activity for many of these alleles remains to be functionally characterized, some do have annotated function such as CYP2C9*61 which is a decreased function allele.

During the development of ClinPharmSeq, a number of limitations have emerged that will be improved in the next version (v2.0). First, ClinPharmSeq has missed the CYP1A2*1L allele entirely because one of its variants (15-75038220-G-A) is in a region that is not targeted by the panel (i.e. located more than 3 kb upstream). Although the enzyme encoded by the star allele has unknown function according to the *CYP1A2* gene page of PharmVar (accessed on October 31, 2021), we are still planning to expand the probes to capture the variant because it has been associated with decreased gene inducibility [37] and because it is commonly found in the populations of Latino/Admixed American, East Asian, and African/African American with allele frequencies of 0.32, 0.28, and 0.27, respectively, according to gnomAD [38]. Second, it has come to our attention that CPIC has recently established new guidelines for nine additional gene-drug pairs with level A involving the *IFNL4* and *MT-RNR1* genes; therefore, we will include these genes in the future. The third limitation is our use of DNA from cell lines with a relatively small sample size (N = 64). Even though this limitation is ameliorated by the fact that our sample set is of vastly diverse ancestry (i.e. 11 populations), for the next version we are planning to test ClinPharmSeq against DNA from real patients with a much larger sample size.

Primarily aimed at broad implementation of PGx testing and studies of large sample sizes, ClinPharmSeq strikes an advantageous balance between sequencing expense, multiplexing capability, and depth of coverage. For instance, the small probe size of ClinPharmSeq puts the panel into the design group of Tier 2 with 0.5–2.999 Mb, which is the second most economic option among the SureSelect NGS target enrichment panels (Tiers 1–5 and Tier L). Therefore, compared to other known methods to survey the comparable PGx space such as WGS and whole exome sequencing, ClinPharmSeq can bring significant data-generation savings. Although the exact cost will depend on various factors including read length and sequencers used, we expect more than 50% reduction in cost compared to both WGS and whole exome sequencing at standard throughputs required for typical germline analysis. In addition to the data-generation savings, the data storage and analysis costs (e.g. computational memory required for analysis) are also much less and more efficient compared to whole genome or whole exome sequencing. At standard throughputs, WGS and whole exome sequencing usually require about 100 and 10 Gb of storage per sample, respectively, while ClinPharmSeq only requires about 1 Gb per sample. SNP arrays, although comparably priced, do not assay the entire gene space that the ClinPharmSeq probes do and are thus inferior to the sequence data produced by ClinPharmSeq. Additionally, there are currently no bioinformatics tools (e.g. PyPGx) that can reliably detect SVs in PGx genes from whole exome sequencing or SNP array data. Finally, the deep coverage inherent to targeted sequencing data enables the discovery of rare variation of potential clinical impact.

In summary, by leveraging NGS data we confirmed the genotype results reported by previous publications and also expanded the current PGx variation catalogs for the 70 important reference samples. Therefore, our ClinPharmSeq characterization can be added to this public reference resource for other PGx genotyping projects. As adverse drug reaction events are a significant cause of morbidity in the US, a platform that can accurately detect and genotype variants, both common and rare, that affect drug response has the potential to both deepen our understanding of these events as well as reduce their incidence. This study shows that targeted sequencing data coupled with a bioinformatics tool can provide not only accurate but also a comprehensive platform for PGx testing compared with multiple standard approaches. By allowing automated, accurate, and comprehensive genotyping of pharmacogenes, the combination between targeted sequencing and genotyping tools offers a feasible path for broad implementation of PGx testing and the optimization of individual drug treatment responses.

## Supporting information

S1 Fig Example of the ‘custom’ probe design used in ClinPharmSeq. Probes for the *CYP2B6* gene are shown as a representative example.
(TIF)

S2 Fig Assessment of uniformity in sequencing coverage for ClinPharmSeq. The left panel shows that, for the most part, ClinPharmSeq coverage is normally distributed, centered at around 270x. The right panel shows that more than 80% of targeted bases have coverage ≥100x. These results suggest that ClinPharmSeq can generate deep-coverage data with relatively high uniformity in a reproducible manner.
(TIF)

S3 Fig Concordance of predicted phenotypes for nine pharmacogenes between WGS, ClinPharmSeq, and previous studies. (TIF)

S4 Fig Incorrectly sequenced sample NA18973. WGS and ClinPharmSeq data are shown in the top and bottom panels, respectively. Each panel contains a copy number profile and an allele fraction profile created by PyPGx.
(TIF)

S1 Table Demographic and sequencing information for 70 reference samples. (DOCX)

S2 Table Diplotype calls for 70 reference samples and 27 pharmacogenes identified by PyPGx’s analysis of whole genome sequencing and ClinPharmSeq data. (DOCX)

S3 Table Discrepancies in diplotype calling between previous publications and PyPGx’s analysis of whole genome sequencing and ClinPharmSeq data. (DOCX)

S4 Table Star alleles excluded from comparison analysis with previously published diplotype data. (DOCX)

S5 Table Phenotype calls for 70 reference samples and nine pharmacogenes identified by PyPGx’s analysis of whole genome sequencing and ClinPharmSeq data. (DOCX)

S6 Table Discrepancies in phenotype calling between previous publications and PyPGx’s analysis of whole genome sequencing and ClinPharmSeq data. (DOCX)

## References

[1] Evans WE and Relling MV. Moving towards individualized medicine with pharmacogenomics. Nature. 2004 May 27;429(6990):464–8. doi: 10.1038/nature02626 15164072

[2] Sullivan-Klose TH, Ghanayem BI, Bell DA, Zhang ZY, Kaminsky LS, Shenfield GM, et al. The role of the CYP2C9-Leu359 allelic variant in the tolbutamide polymorphism. Pharmacogenetics. 1996 Aug;6(4):341–9. doi: 10.1097/00008571-199608000-00007 8873220

[3] Kidd RS, Curry TB, Gallagher S, Edeki T, Blaisdell J, Goldstein JA. Identification of a null allele of CYP2C9 in an African-American exhibiting toxicity to phenytoin. Pharmacogenetics. 2001 Dec;11(9):803–8. doi: 10.1097/00008571-200112000-00008 11740344

[4] Sanderson S, Emery J, Higgins J. CYP2C9 gene variants, drug dose, and bleeding risk in warfarin-treated patients: a HuGEnet systematic review and meta-analysis. Genet Med. 2005 Feb;7(2):97–104. doi: 10.1097/01.gim.0000153664.65759.cf 15714076

[5] Van Driest SL, Shi Y, Bowton EA, Schildcrout JS, Peterson JF, Pulley J, et al. Clinically actionable genotypes among 10,000 patients with preemptive pharmacogenomic testing. Clin Pharmacol Ther. 2014 Apr;95(4):423–31. doi: 10.1038/clpt.2013.229 Epub 2013 Nov 19. 24253661 PMC3961508

[6] Relling MV and Klein TE. CPIC: Clinical Pharmacogenetics Implementation Consortium of the Pharmacogenomics Research Network. Clin Pharmacol Ther. 2011 Mar;89(3):464–7. doi: 10.1038/clpt.2010.279 Epub 2011 Jan 26. 21270786 PMC3098762

[7] Daly AK and Cascorbi I. Opportunities and limitations: the value of pharmacogenetics in clinical practice. Br J Clin Pharmacol. 2014 Apr;77(4):583–6. doi: 10.1111/bcp.12354 24548248 PMC3971974

[8] Swen JJ, Nijenhuis M, Boer Ad, Grandia L, Maitland-van der Zee AH, Mulder H, et al. Pharmacogenetics: from bench to byte—an update of guidelines. Clin Pharmacol Ther. 2011 May;89(5):662–73. doi: 10.1038/clpt.2011.34 Epub 2011 Mar 16. 21412232

[9] Gaedigk A, Ingelman-Sundberg M, Miller NA, Leeder JS, Whirl-Carrillo M, Klein TE, et al. The Pharmacogene Variation (PharmVar) Consortium: Incorporation of the Human Cytochrome P450 (CYP) Allele Nomenclature Database. Clin Pharmacol Ther. 2018 Mar;103(3):399–401. doi: 10.1002/cpt.910 Epub 2017 Nov 14. 29134625 PMC5836850

[10] Gaedigk A, Simon SD, Pearce RE, Bradford LD, Kennedy MJ, Leeder JS. The CYP2D6 activity score: translating genotype information into a qualitative measure of phenotype. Multicenter Study Clin Pharmacol Ther. 2008 Feb;83(2):234–42. doi: 10.1038/sj.clpt.6100406 Epub 2007 Oct 31. 17971818

[11] Gaedigk A, Sangkuhl K, Whirl-Carrillo M, Klein T, Leeder JS. Prediction of CYP2D6 phenotype from genotype across world populations. Genet Med. 2017 Jan;19(1):69–76. doi: 10.1038/gim.2016.80 Epub 2016 Jul 7. 27388693 PMC5292679

[12] Gaedigk A. Complexities of CYP2D6 gene analysis and interpretation. Int Rev Psychiatry. 2013 Oct;25(5):534–53. doi: 10.3109/09540261.2013.825581 24151800

[13] Gaedigk A, Ndjountché L, Divakaran K, Bradford LD, Zineh I, Oberlander TF, et al. Cytochrome P4502D6 (CYP2D6) gene locus heterogeneity: characterization of gene duplication events. Clin Pharmacol Ther. 2007 Feb;81(2):242–51. doi: 10.1038/sj.clpt.6100033 17259947

[14] Twist GP, Gaedigk A, Miller NA, Farrow EG, Willig LK, Dinwiddie DL, et al. Constellation: a tool for rapid, automated phenotype assignment of a highly polymorphic pharmacogene, CYP2D6, from whole-genome sequences. NPJ Genom Med. 2016 Jan 13;1:15007. doi: 10.1038/npjgenmed.2015.7 eCollection 2016. 29263805 PMC5685293

[15] Klein TE and Ritchie MD. PharmCAT: A Pharmacogenomics Clinical Annotation Tool. Clin Pharmacol Ther. 2018 Jul;104(1):19–22. doi: 10.1002/cpt.928 Epub 2017 Dec 1. 29194583 PMC5984125

[16] Numanagić I, Malikić S, Ford M, Qin X, Toji L, Radovich M, et al. Allelic decomposition and exact genotyping of highly polymorphic and structurally variant genes. Nat Commun. 2018 Feb 26;9(1):828. doi: 10.1038/s41467-018-03273-1 29483503 PMC5826927

[17] Lee SB, Wheeler MM, Patterson K, McGee S, Dalton R, Woodahl EL, et al. Stargazer: a software tool for calling star alleles from next-generation sequencing data using CYP2D6 as a model. Genet Med. 2019 Feb;21(2):361–372. doi: 10.1038/s41436-018-0054-0 Epub 2018 Jun 6. 29875422 PMC6281872

[18] Lee SB, Wheeler MM, Thummel KE, Nickerson DA. Calling Star Alleles With Stargazer in 28 Pharmacogenes With Whole Genome Sequences. Clin Pharmacol Ther. 2019 Dec;106(6):1328–1337. doi: 10.1002/cpt.1552 Epub 2019 Jul 26. 31206625 PMC6896231

[19] Dalton R, Lee SB, Claw KG, Prasad B, Phillips BR, Shen DD, et al. Interrogation of CYP2D6 Structural Variant Alleles Improves the Correlation Between CYP2D6 Genotype and CYP2D6-Mediated Metabolic Activity. Clin Transl Sci. 2020 Jan;13(1):147–156. doi: 10.1111/cts.12695 Epub 2019 Oct 25. 31536170 PMC6951848

[20] Claw KG, Beans JA, Lee SB, Avey JP, Stapleton PA, Scherer SE, et al. Pharmacogenomics of Nicotine Metabolism: Novel CYP2A6 and CYP2B6 Genetic Variation Patterns in Alaska Native and American Indian Populations. Nicotine Tob Res. 2020 May 26;22(6):910–918. doi: 10.1093/ntr/ntz105 31241144 PMC7249913

[21] McInnes G, Dalton R, Sangkuhl K, Whirl-Carrillo M, Lee SB, Tsao PS, et al. Transfer learning enables prediction of CYP2D6 haplotype function. PLoS Comput Biol. 2020 Nov 2;16(11): e1008399. doi: 10.1371/journal.pcbi.1008399 eCollection 2020 Nov. 33137098 PMC7660895

[22] Pratt VM, Everts RE, Aggarwal P, Beyer BN, Broeckel U, Epstein-Baak R, et al. Characterization of 137 Genomic DNA Reference Materials for 28 Pharmacogenetic Genes: A GeT-RM Collaborative Project. J Mol Diagn. 2016 Jan;18(1):109–23. doi: 10.1016/j.jmoldx.2015.08.005 Epub 2015 Nov 24. 26621101 PMC4695224

[23] Gaedigk A, Turner A, Everts RE, Scott SA, Aggarwal P, Broeckel U, et al. Characterization of Reference Materials for Genetic Testing of CYP2D6 Alleles: A GeT-RM Collaborative Project. J Mol Diagn. 2019 Nov;21(6):1034–1052. doi: 10.1016/j.jmoldx.2019.06.007 Epub 2019 Aug 9. 31401124 PMC6854474

[24] Pratt VM, Turner A, Broeckel U, Dawson DB, Gaedigk A, Lynnes TC, et al. Characterization of Reference Materials with an Association for Molecular Pathology Pharmacogenetics Working Group Tier 2 Status: CYP2C9, CYP2C19, VKORC1, CYP2C Cluster Variant, and GGCX: A GeT-RM Collaborative Project. J Mol Diagn. 2021 Aug;23(8):952–958. doi: 10.1016/j.jmoldx.2021.04.012 Epub 2021 May 19. 34020041 PMC8491090

[25] Whirl-Carrillo M, Huddart R, Gong L, Sangkuhl K, Thorn CF, Whaley R, et al. An Evidence-Based Framework for Evaluating Pharmacogenomics Knowledge for Personalized Medicine. Clin Pharmacol Ther. 2021 Sep;110(3):563–572. doi: 10.1002/cpt.2350 Epub 2021 Jul 22. 34216021 PMC8457105

[26] Gordon AS, Fulton RS, Qin X, Mardis ER, Nickerson DA, Scherer S. PGRNseq: a targeted capture sequencing panel for pharmacogenetic research and implementation. Pharmacogenet Genomics. 2016 Apr;26(4):161–168. doi: 10.1097/FPC.0000000000000202 26736087 PMC4935646

[27] MacDonald JR, Ziman R, Yuen RKC, Feuk L, Scherer SW. The Database of Genomic Variants: a curated collection of structural variation in the human genome. Nucleic Acids Res. 2014 Jan;42(Database issue): D986–92. doi: 10.1093/nar/gkt958 Epub 2013 Oct 29. 24174537 PMC3965079

[28] Li H and Durbin R. Fast and accurate short read alignment with Burrows-Wheeler transform. Bioinformatics. 2009 Jul 15;25(14):1754–60. doi: 10.1093/bioinformatics/btp324 Epub 2009 May 18. 19451168 PMC2705234

[29] Li H, Handsaker B, Wysoker A, Fennell T, Ruan J, Homer N, et al. The Sequence Alignment/Map format and SAMtools. Bioinformatics. 2009 Aug 15;25(16):2078–9. doi: 10.1093/bioinformatics/btp352 Epub 2009 Jun 8. 19505943 PMC2723002

[30] McKenna A, Hanna M, Banks E, Sivachenko A, Cibulskis K, Kernytsky A, et al. The Genome Analysis Toolkit: a MapReduce framework for analyzing next-generation DNA sequencing data. Genome Res. 2010 Sep;20(9):1297–303. doi: 10.1101/gr.107524.110 Epub 2010 Jul 19. 20644199 PMC2928508

[31] Raczy C, Petrovski R, Saunders CT, Chorny I, Kruglyak S, Margulies EH, et al. Isaac: ultra-fast whole-genome secondary analysis on Illumina sequencing platforms. Bioinformatics. 2013 Aug 15;29(16):2041–3. doi: 10.1093/bioinformatics/btt314 Epub 2013 Jun 4. 23736529

[32] Browning SR and Browning BL. Rapid and accurate haplotype phasing and missing-data inference for whole-genome association studies by use of localized haplotype clustering. Am J Hum Genet. 2007 Nov;81(5):1084–97. doi: 10.1086/521987 Epub 2007 Sep 21. 17924348 PMC2265661

[33] 1000 Genomes Project Consortium. A global reference for human genetic variation. Nature. 2015 Oct 1;526(7571):68–74. doi: 10.1038/nature15393 26432245 PMC4750478

[34] Pitarque M, von Richter O, Oke B, Berkkan H, Oscarson M, Ingelman-Sundberg M. Identification of a single nucleotide polymorphism in the TATA box of the CYP2A6 gene: impairment of its promoter activity. Biochem Biophys Res Commun. 2001 Jun 8;284(2):455–60. doi: 10.1006/bbrc.2001.4990 11394901

[35] Iyer L, Das S, Janisch L, Wen M, Ramírez J, Karrison T, et al. UGT1A1*28 polymorphism as a determinant of irinotecan disposition and toxicity. Pharmacogenomics J. 2002;2(1):43–7. doi: 10.1038/sj.tpj.6500072 11990381

[36] Kuehl P, Zhang J, Lin Y, Lamba J, Assem M, Schuetz J, et al. Sequence diversity in CYP3A promoters and characterization of the genetic basis of polymorphic CYP3A5 expression. Nat Genet. 2001 Apr;27(4):383–91. doi: 10.1038/86882 11279519

[37] Nakajima M, Yokoi T, Mizutani M, Kinoshita M, Funayama M, Kamataki T. Genetic polymorphism in the 5’-flanking region of human CYP1A2 gene: effect on the CYP1A2 inducibility in humans. J Biochem. 1999 Apr;125(4):803–8. doi: 10.1093/oxfordjournals.jbchem.a022352 10101295

[38] Karczewski KJ, Francioli LC, Tiao G, Cummings BB, Alföldi J, Wang Q, et al. The mutational constraint spectrum quantified from variation in 141,456 humans. Nature. 2020 May;581(7809):434–443. doi: 10.1038/s41586-020-2308-7 Epub 2020 May 27. 32461654 PMC7334197

