# Supplementary figures and images for: ClinPharmSeq: A targeted sequencing panel for clinical pharmacogenetics implementation
Source: PLoS One. 2022 Jul 28;17(7):e0272129. doi: 10.1371/journal.pone.0272129 (PMC9333201; doi:10.1371/journal.pone.0272129)

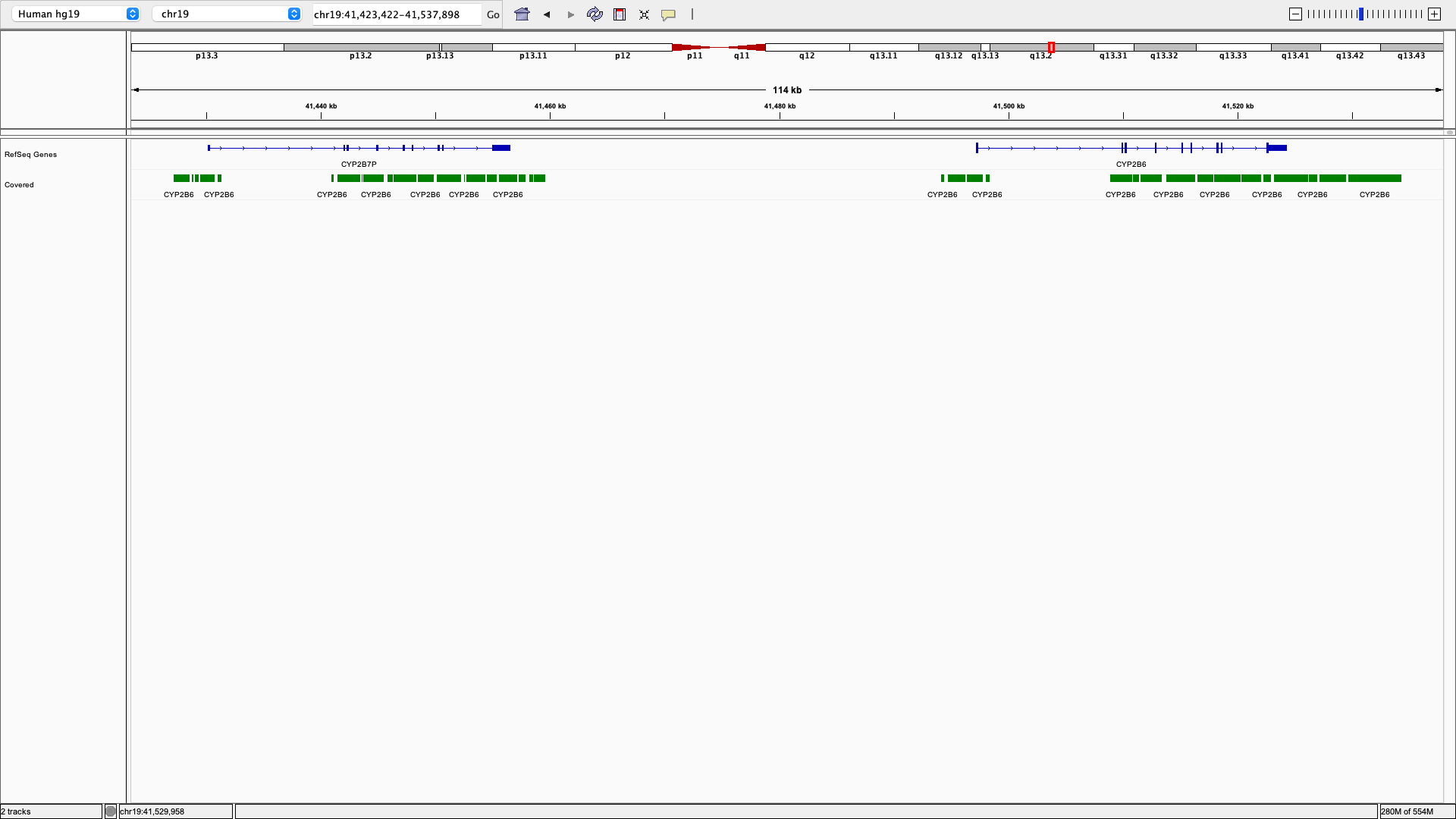

Supplement: S1 Fig — Probes for the CYP2B6 gene are shown as a representative example. (TIF) [file pone.0272129.s001.tif]

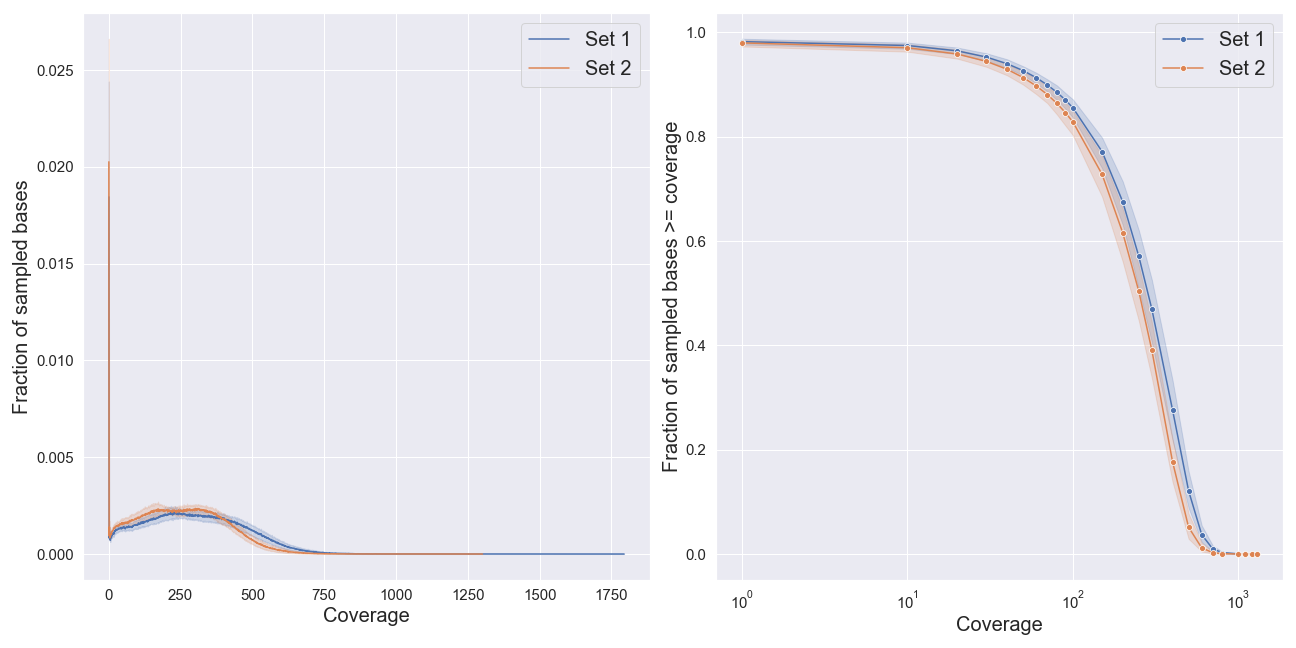

Supplement: S2 Fig — The left panel shows that, for the most part, ClinPharmSeq coverage is normally distributed, centered at around 270x. The right panel shows that more than 80% of targeted bases have coverage ≥100x. These results suggest that ClinPharmSeq can generate deep-coverage data with relatively high uniformity in a reproducible manner. (TIF) [file pone.0272129.s002.tif]

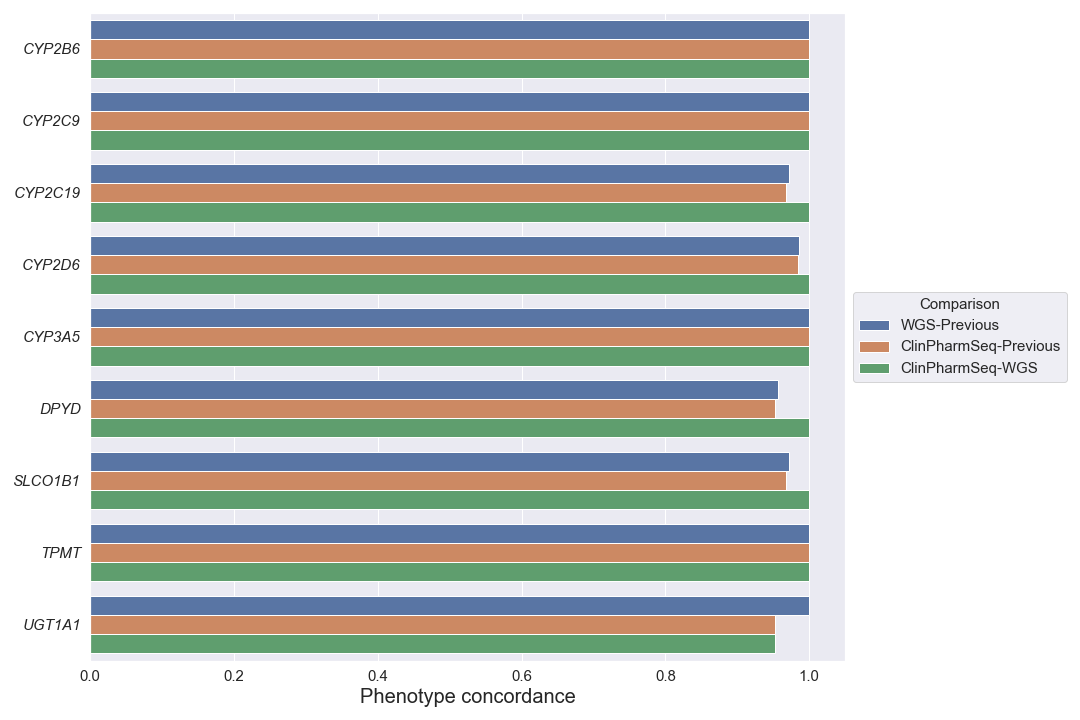

Supplement: S3 Fig — (TIF) [file pone.0272129.s003.tif]

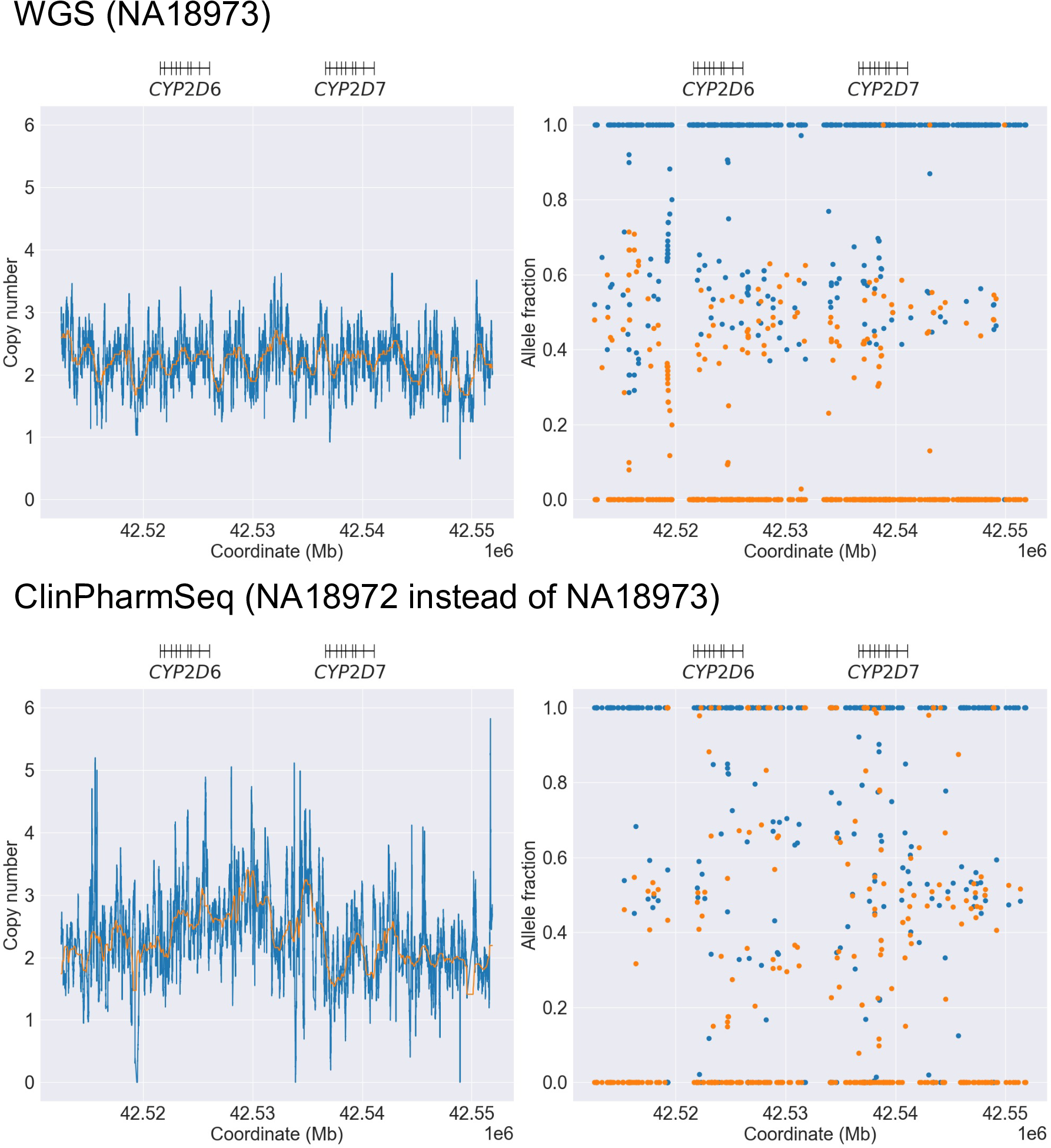

Supplement: S4 Fig — WGS and ClinPharmSeq data are shown in the top and bottom panels, respectively. Each panel contains a copy number profile and an allele fraction profile created by PyPGx. (TIF) [file pone.0272129.s004.tif]
