# Supplementary material for: ClinPharmSeq: A targeted sequencing panel for clinical pharmacogenetics implementation
Source: PLoS One. 2022 Jul 28;17(7):e0272129. doi: 10.1371/journal.pone.0272129 (PMC9333201; doi:10.1371/journal.pone.0272129)
Supplement: S1 Table — (DOCX) [file pone.0272129.s005.docx]

| No. | Coriell ID | BAM ID | GeT-RM | Sex | Superpopulation | Population | Test |
| --- | --- | --- | --- | --- | --- | --- | --- |
| 1 | NA10831 | 021ab129bb594be5804b02e08e14d93d | Tier 1 | Female | European | Utah/Mormon | Set 1 |
| 2 | NA18855 | 03bc76a2c27140bc8143c56767ca6877 | Tier 1 | Female | African | Yoruba | Set 1 |
| 3 | NA18617 | 0b7cc95044c54d86a81151d856d0c5b2 | Tier 2 | Female | East Asian | Han Chinese | Set 2 |
| 4 | NA19908 | 0d73bafef55a4a718489f3fdca91fd55 | Tier 1 | Male | African | African ancestry | Set 1 |
| 5 | NA18973 | 11a1c7b37a63449f9bc65799567b5710 | Tier 1 | Female | East Asian | Japanese | N/A |
| 6 | NA12003 | 141390df39414eaaaa9725a8349d9c45 | Tier 1 | Male | European | Utah/Mormon | Set 1 |
| 7 | NA18519 | 20aaa40fcdee4290bfada8dbba5e5232 | Tier 1 | Male | African | Yoruba | Set 1 |
| 8 | HG00276 | 20b87673c1224e9db8bdbbe82899309c | Tier 1 | Female | European | Finnish | Set 1 |
| 9 | NA11993 | 20be9cf6bed64502b85c999ec59c784b | Tier 1 | Female | European | Utah/Mormon | Set 1 |
| 10 | NA19917 | 2176d8cef950450bbb29052359a2d2d1 | Tier 2 | Female | African | African ancestry | Set 2 |
| 11 | NA19920 | 228fa4f74dd4431f8eb3526f37e355ed | Tier 1 | Male | African | African ancestry | Set 1 |
| 12 | NA11832 | 22d9ea3d16804243afbfea7e776c5237 | Tier 1 | Female | European | Utah/Mormon | Set 2 |
| 13 | NA07029 | 28997710cbae49f5996f6075fcbf74bd | Tier 1 | Male | European | Utah/Mormon | N/A |
| 14 | NA18868 | 2b0b4c79e8104ed98ef5cc82d9ca8bd2 | Tier 1 | Male | African | Yoruba | Set 1 |
| 15 | NA12813 | 2c9f234af49b4f6a970d8ddef07358e5 | Tier 1 | Female | European | Utah/Mormon | Set 2 |
| 16 | HG00589 | 316ab006177d41b484982d7fa4d851ad | Tier 1 | Male | East Asian | Southern Han Chinese | Set 1 |
| 17 | NA20296 | 34b739132d0e403d9aa323b1c3edf12a | Tier 1 | Female | African | African ancestry | Set 2 |
| 18 | NA12717 | 3959a73552a04f969d37a04dc869c7a9 | Tier 1 | Female | European | Utah/Mormon | Set 2 |
| 19 | NA07056 | 3a7ec2f78f3c40df98248d3ba1354a20 | Tier 1 | Female | European | Utah/Mormon | Set 2 |
| 20 | NA18484 | 3ddccd4bcda14140b3e89559d2cf3186 | Tier 1 | Female | African | Yoruba | Set 2 |
| 21 | NA19178 | 3fcca708192c4ffe8e57318c7d64e480 | Tier 1 | Male | African | Yoruba | Set 1 |
| 22 | NA18564 | 4323a15d7b5d4bf2b204e0c0088ba923 | Tier 1 | Female | East Asian | Han Chinese | N/A |
| 23 | NA12145 | 45e24be4dc7d4cb3a2742ce7c05730e8 | Tier 1 | Female | European | Utah/Mormon | Set 2 |
| 24 | NA18861 | 543558ae08cd44b3850fc7b835484037 | Tier 1 | Female | African | Yoruba | Set 1 |
| 25 | HG00436 | 54db734bc1ec46b29fc6c5c6df35ca65 | Tier 1 | Male | East Asian | Southern Han Chinese | Set 1 |
| 26 | NA18552 | 5bb329dcb3654e8890985547e82135f6 | Tier 1 | Female | East Asian | Han Chinese | Set 1 |
| 27 | NA07000 | 5d81e03c86324f209c69093ddf77bb62 | Tier 2 | Female | European | Utah/Mormon | Set 1 |
| 28 | NA12006 | 5f006ec8ba3c41a18d9ff92c0a62955a | Tier 1 | Female | European | Utah/Mormon | Set 1 |
| 29 | NA19007 | 6320750d96f747b695f78964df1fad17 | Tier 1 | Male | East Asian | Japanese | Set 1 |
| 30 | NA19239 | 69335e244b5f46e5b56e5360f8f826b7 | Tier 1 | Male | African | Yoruba | Set 1 |
| 31 | NA12156 | 69d7b11affce444694e9955b90848028 | Tier 1 | Female | European | Utah/Mormon | Set 1 |
| 32 | NA19147 | 6b9f68c07c3e4e3e81669fe390ba1027 | Tier 1 | Female | African | Yoruba | Set 2 |
| 33 | NA19095 | 75331ab394f24d56ac73cee5d41fa15b | Tier 1 | Female | African | Yoruba | Set 2 |
| 34 | NA10854 | 7866e1bc7fdc4304863db4a25d1a42e4 | Tier 1 | Female | European | Utah/Mormon | Set 1 |
| 35 | NA18980 | 7a29e8a53b6844ddbb42aa165b34fba3 | Tier 1 | Female | East Asian | Japanese | Set 1 |
| 36 | NA19207 | 7a94709935ea412e8b7349828beaf338 | Tier 1 | Male | African | Yoruba | Set 2 |
| 37 | NA18526 | 7d077b89f2514fb0b8e002f8d9a10189 | Tier 1 | Female | East Asian | Han Chinese | Set 2 |
| 38 | NA18959 | 7fdcb4bdabe743feb4abc155bc580b82 | Tier 1 | Male | East Asian | Japanese | N/A |
| 39 | NA06991 | 82b808e2886a42f986da3ba811dbeaa4 | Tier 1 | Female | European | Utah/Mormon | Set 2 |
| 40 | NA19109 | 8f90214c429f4afc9e0da555cb77f89a | Tier 1 | Female | African | Yoruba | Set 2 |
| 41 | NA18952 | 92ac6fc0f69345aabb9e7bd47452ed70 | Tier 1 | Male | East Asian | Japanese | N/A |
| 42 | NA19789 | 932a3f4c888844c39399f1cfdf2bc593 | Tier 1 | Male | American | Mexican ancestry | Set 2 |
| 43 | HG01190 | 9e01734a352a41f89266c2ae8c9c13de | Tier 1 | Male | American | Puerto Rican | Set 2 |
| 44 | NA19122 | 9e3dcdd083ad40dd82fab180a916fb77 | Tier 1 | Female | African | Yoruba | Set 2 |
| 45 | NA19819 | a04f109738f34a358850f5f69d7d8814 | Tier 1 | Female | African | African ancestry | Set 1 |
| 46 | NA19213 | a284eb8f85e54b0ba3c45e30e91a33a1 | Tier 1 | Male | African | Yoruba | Set 2 |
| 47 | NA07055 | a9963d642c584dfab81f5ae694208390 | Tier 1 | Female | European | Utah/Mormon | Set 1 |
| 48 | NA19174 | b462b7941e0642309d5d44e7aae6d42f | Tier 1 | Male | African | Yoruba | N/A |
| 49 | NA20509 | b51b8299a62f4a3eab95d0467e950b7e | Tier 1 | Male | European | Toscani (Tuscan) | Set 2 |
| 50 | NA18992 | b7988ee8179d4678921602d34400a63a | Tier 1 | Female | East Asian | Japanese | Set 1 |
| 51 | NA18565 | b859eb68840b42d9a376ce22cff1cd08 | Tier 1 | Female | East Asian | Han Chinese | Set 2 |
| 52 | NA18524 | ba64fb6dbb0a4b36a5cbe53bd8706ca7 | Tier 1 | Male | East Asian | Han Chinese | Set 1 |
| 53 | NA18942 | bda28ac619e64916bb0a28d1f2698e2e | Tier 1 | Female | East Asian | Japanese | Set 2 |
| 54 | NA11839 | c392e9300f6b490aa1c43f7ca7d7afd4 | Tier 1 | Male | European | Utah/Mormon | Set 2 |
| 55 | NA10851 | cb484635e004493fb395ac764578797b | Tier 1 | Male | European | Utah/Mormon | Set 2 |
| 56 | NA19176 | d7b5cf7015d44c23a949dc117c149c80 | Tier 1 | Female | African | Yoruba | Set 2 |
| 57 | NA18509 | d84c8e4613064299b2a16cfa39d819b5 | Tier 1 | Male | African | Yoruba | Set 2 |
| 58 | NA19226 | db923feb258241b8b0a3e3aaada9359b | Tier 1 | Male | African | Yoruba | Set 1 |
| 59 | NA07357 | dd64f80e456a46e49555c0c7c30372b0 | Tier 1 | Male | European | Utah/Mormon | Set 2 |
| 60 | NA07019 | dd864a425f814bca87a971eaf94cfa17 | Tier 1 | Female | European | Utah/Mormon | Set 1 |
| 61 | NA12873 | def4a73760dd42d38b17c33c3deb654b | Tier 1 | Female | European | Utah/Mormon | Set 2 |
| 62 | NA19143 | dfe4ba40717d4891a12ec2c856de671f | Tier 1 | Female | African | Yoruba | Set 1 |
| 63 | NA10847 | e030f757080d4e5e841d8e7feef7a665 | Tier 1 | Female | European | Utah/Mormon | N/A |
| 64 | NA18518 | e1ddd983797640bf81b66f9a77b37439 | Tier 1 | Female | African | Yoruba | Set 2 |
| 65 | NA21781 | e246a44270e34d2aa228844732995abe | Tier 2 | Male | Unknown | Unknown (Caucasian) | Set 2 |
| 66 | NA07348 | e92d97eb8a3f4c4cb7db5faf9882b167 | Tier 1 | Female | European | Utah/Mormon | Set 1 |
| 67 | NA19003 | e968235bb8e14f02af53e2f17cee324f | Tier 1 | Female | East Asian | Japanese | Set 1 |
| 68 | NA18544 | ecf7d003ffef4d429351037cb97bac09 | Tier 1 | Male | East Asian | Han Chinese | Set 1 |
| 69 | NA18540 | f00e1071f840476c9872de73f0ea8a02 | Tier 1 | Female | East Asian | Han Chinese | Set 2 |
| 70 | NA18966 | f52c133442ca4f93a47b689fc385b1f5 | Tier 1 | Male | East Asian | Japanese | Set 1 |
