# Supplementary material for: ClinPharmSeq: A targeted sequencing panel for clinical pharmacogenetics implementation
Source: PLoS One. 2022 Jul 28;17(7):e0272129. doi: 10.1371/journal.pone.0272129 (PMC9333201; doi:10.1371/journal.pone.0272129)
Supplement: S2 Table — (DOCX) [file pone.0272129.s006.docx]

|  | ***CYP1A2*** |  |  | ***CYP2A6*** |  |  | ***CYP2B6*** |  |  |
| --- | --- | --- | --- | --- | --- | --- | --- | --- | --- |
| **Coriell ID** | **Previous^1^** | **WGS** | **ClinPharmSeq** | **Previous^1^** | **WGS** | **ClinPharmSeq** | **Previous^1^** | **WGS** | **ClinPharmSeq** |
| NA10831 | **1A/*1F* | **1A/*1F* | **1A/*1A [X]* | **1/*2* | **1/*2* | **1/*2* | **1/*1* | **1/*1* | **1/*1* |
| NA18855 | **1A/*1L* | **1A/*1L* | **1A/*1F [N]* | **1/*1* | **1/*1* | **1/*1* | **6/*6* | **6/*6* | **6/*6* |
| NA18617 | **1A/*1A* | **1A/*1A* | **1A/*1A* | **1/*4* | **1/*4* | **1/*4* | **1/*4* | **1/*4* | **1/*4* |
| NA19908 | **1L/*1L* | **1L/*1L* | **1F/*1F [N]* | **1/*17* | **1/*17* | **1/*17* | **1/*6* | **1/*6* | **1/*6* |
| NA18973 | **1F/*1L* | **1F/*1L* | *N/A* | **4/*4* | **4/*4* | *N/A* | **1/*6* | **1/*6* | *N/A* |
| NA12003 | **1F/*1F* | **1F/*1F* | **1F/*1F* | **1/*1* | **1/*1* | **1/*1* | **6/*6* | **6/*6* | **6/*6* |
| NA18519 | **1A/*1L* | **1A/*1L* | **1A/*1F [N]* | **1/*1* | **1/*1* | **1/*1* | **6/*17* | **6/*17* | **6/*17* |
| HG00276 | **1A/*1F* | **1A/*1F* | **1A/*1F* | **1/*1* | **1/*1* | **1/*1* | **2/*4* | **2/*4* | **2/*4* |
| NA11993 | **1F/*1F* | **1F/*1F* | **1F/*1F* | **9/*17* | **9/*17* | **9/*17* | **1/*1* | **1/*1* | **1/*1* |
| NA19917 | **1A/*1F* | **1A/*1F* | **1A/*1F* | **1/*1* | **1/*1* | **1/*1* | **6/*6* | **6/*6* | **6/*6* |
| NA19920 | **1A/*1A* | **1A/*1A* | **1A/*1A* | **1/*35* | **1/*1* | **1/*1* | **6/*6* | **6/*6* | **6/*6* |
| NA11832 | **1F/*1F* | **1F/*1F* | **1F/*1F* | **1/*2* | **1/*2* | **1/*2* | **1/*1* | **1/*1* | **1/*1* |
| NA07029 | **1A/*1F* | **1A/*1F* | *N/A* | **1/*1* | **1/*1* | *N/A* | **1/*6* | **1/*6* | *N/A* |
| NA18868 | **1A/*1L* | **1A/*1L* | **1A/*1F [N]* | **1/*17* | **1/*17* | **1/*17* | **1/*6* | **1/*6* | **1/*6* |
| NA12813 | **1F/*1L* | **1F/*1L* | **1F/*1F [N]* | **1/*1* | **1/*1* | **1/*1* | **1/*2* | **1/*2* | **1/*2* |
| HG00589 | **1A/*1L* | **1A/*1L* | **1A/*1F [N]* | **1/*1* | **1/*1* | **1/*1* | **1/*1* | **1/*1* | **1/*1* |
| NA20296 | **1L/*1L* | **1L/*1L* | **1F/*1F [N]* | **1/*1* | **1/*1* | **1/*1* | **1/*2* | **1/*2* | **1/*2* |
| NA12717 | **1F/*1F* | **1F/*1F* | **1F/*1F* | **1/*1* | **1/*1* | **1/*1* | **5/*6* | **5/*6* | **5/*6* |
| NA07056 | **1A/*1A* | **1A/*1A* | **1A/*1A* | **1/*1* | **1/*1* | **1/*1* | **6/*22* | **6/*22* | **6/*22* |
| NA18484 | **1A/*1A* | **1A/*1A* | **1A/*1A* | **1/*9* | **1/*9* | **1/*9* | **1/*18* | **1/*18* | **1/*18* |
| NA19178 | **1L/*1L* | **1L/*1L* | **1F/*1F [N]* | **1/*20* | **1/*20* | **1/*20* | **6/*29* | **6/*29* | **6/*29* |
| NA18564 | **1A/*1A* | **1A/*1A* | *N/A* | **1/*1* | **1/*1* | *N/A* | **1/*1* | **1/*1* | *N/A* |
| NA12145 | **1A/*1A* | **1A/*1A* | **1A/*1A* | **1/*1* | **1/*1* | **1/*1* | **1/*1* | **1/*1* | **1/*1* |
| NA18861 | **1A/*1L* | **1A/*1L* | **1A/*1F [N]* | **1x2/*25* | **1x2/*25* | **1x2/*25* | **1/*18* | **1/*18* | **1/*18* |
| HG00436 | **1A/*1F* | **1A/*1F* | **1A/*1F* | **4/*1+*S6* | *Indeterminate [X]* | *Indeterminate [X]* | **1/*6* | **1/*6* | **1/*6* |
| NA18552 | **1A/*1A* | **1A/*1A* | **1A/*1A* | **9/*9* | **9/*9* | **9/*9* | **1/*6* | **1/*6* | **1/*6* |
| NA07000 | **1F/*1F* | **1F/*1F* | **1F/*1F* | **1/*1* | **1/*1* | **1/*1* | **1/*1* | **1/*1* | **1/*1* |
| NA12006 | **1F/*1F* | **1F/*1F* | **1F/*1F* | **1/*1* | **1/*1* | **1/*1* | **1/*5* | **1/*5* | **1/*5* |
| NA19007 | **1A/*1L* | **1A/*1L* | **1A/*1F [N]* | **1/*4* | **1/*4* | **1/*4* | **1/*23* | **1/*23* | **1/*23* |
| NA19239 | **1A/*1L* | **1A/*1L* | **1A/*1F [N]* | **1/*17* | **1/*17* | **1/*17* | **1/*6* | **1/*6* | **1/*6* |
| NA12156 | **1A/*1F* | **1A/*1F* | **1A/*1F* | **1/*1* | **1/*1* | **1/*1* | **1/*1* | **1/*1* | **1/*1* |
| NA19147 | **1A/*1A* | **1A/*1A* | **1A/*1A* | **9/*23* | **9/*23* | **9/*23* | **1/*18* | **1/*18* | **1/*18* |
| NA19095 | **1A/*1L* | **1A/*1L* | **1A/*1F [N]* | **9/*9* | **9/*9* | **9/*9* | **18/*18* | **18/*18* | **18/*18* |
| NA10854 | **1A/*1A* | **1A/*1A* | **1A/*1A* | **1/*1* | **1/*1* | **1/*1* | **1/*1* | **1/*1* | **1/*1* |
| NA18980 | **1A/*1L* | **1A/*1L* | **1A/*1F [N]* | **9/*9* | **9/*9* | **9/*9* | **6/*6* | **6/*6* | **6/*6* |
| NA19207 | **1A/*1F* | **1A/*1F* | **1A/*1F* | **1/*17* | **1/*17* | **1/*17* | **1/*6* | **1/*6* | **1/*6* |
| NA18526 | **1A/*1L* | **1A/*1L* | **1A/*1F [N]* | **7/*9* | **7/*9* | **7/*9* | **1/*1* | **1/*1* | **1/*1* |
| NA18959 | **1A/*1A* | **1A/*1A* | *N/A* | **1/*4* | **1/*4* | *N/A* | **1/*1* | **1/*1* | *N/A* |
| NA06991 | **1F/*1F* | **1F/*1F* | **1F/*1F* | **1/*1* | **1/*1* | **1/*1* | **1/*6* | **1/*6* | **1/*6* |
| NA19109 | **1A/*1F* | **1A/*1F* | **1A/*1F* | **17/*20* | **17/*20* | **17/*20* | **1/*6* | **1/*6* | **1/*6* |
| NA18952 | **1A/*1L* | **1A/*1L* | *N/A* | **4/*4* | **4/*4* | *N/A* | **1/*1* | **1/*1* | *N/A* |
| NA19789 | **1A/*1L* | **1A/*1L* | **1A/*1F [N]* | **1/*1* | **1/*1* | **1/*1* | **1/*1* | **1/*1* | **1/*1* |
| HG01190 | **1A/*1A* | **1A/*1A* | **1A/*1A* | **1/*1* | **1/*1* | **1/*1* | **1/*5* | **1/*5* | **1/*5* |
| NA19122 | **1A/*1L* | **1A/*1L* | **1A/*1F [N]* | **1/*35* | **1/*1* | **1/*1* | **6/*6* | **6/*6* | **6/*6* |
| NA19819 | **1F/*1L* | **1F/*1L* | **1F/*1F [N]* | **1/*1* | **1/*1* | **1/*1* | **1/*1* | **1/*1* | **1/*1* |
| NA19213 | **1A/*1A* | **1A/*1A* | **1A/*1A* | **17/*24* | **17/*24* | **17/*24* | **6/*6* | **6/*6* | **6/*6* |
| NA07055 | **1F/*1F* | **1F/*1F* | **1F/*1F* | **1/*22* | **1/*22* | **1/*1 [X]* | **1/*6* | **1/*6* | **1/*6* |
| NA19174 | **1A/*1L* | **1A/*1L* | *N/A* | **1/*9* | **9/*24* | *N/A* | **6/*18* | **6/*18* | *N/A* |
| NA20509 | **1A/*1F* | **1A/*1F* | **1A/*1F* | **1/*1* | **1/*1* | **1/*1* | **5/*6* | **5/*6* | **5/*9* |
| NA18992 | **1A/*1A* | **1A/*1A* | **1A/*1A* | **9/*19* | **7/*18* | **7/*18* | **1/*6* | **1/*6* | **1/*6* |
| NA18565 | **1F/*1F* | **1F/*1F* | **1F/*1F* | **4/*15* | **4/*15* | **4/*15* | **1/*1* | **1/*1* | **1/*1* |
| NA18524 | **1A/*1F* | **1A/*1F* | **1A/*1F* | **1/*1* | **1/*1* | **1/*1* | **1/*1* | **1/*1* | **1/*1* |
| NA18942 | **1A/*1F* | **1A/*1F* | **1A/*1F* | **4/*7* | **4/*7* | **4/*7* | **1/*2* | **1/*2* | **1/*2* |
| NA11839 | **1A/*1F* | **1A/*1F* | **1A/*1F* | **1/*9* | **1/*9* | **1/*9* | **1/*15* | **1/*15* | **1/*15* |
| NA10851 | **1F/*1F* | **1F/*1F* | **1F/*1F* | **1/*2* | **1/*2* | **1/*2* | **1/*1* | **1/*1* | **1/*1* |
| NA19176 | **1A/*1A* | **1A/*1A* | **1A/*1A* | **1/*1* | **1/*1* | **1/*1* | **6/*6* | **6/*6* | **6/*6* |
| NA18509 | **1A/*1F* | **1A/*1F* | **1A/*1F* | **1/*17* | **1/*17* | **1/*17* | **1/*6* | **1/*6* | **1/*6* |
| NA19226 | **1A/*1A* | **1A/*1A* | **1A/*1A* | **1/*17* | **1/*17* | **1/*17* | **18/*20* | **18/*20* | **18/*20* |
| NA07357 | **1F/*1F* | **1F/*1F* | **1F/*1F* | **1/*1* | **1/*1* | **1/*1* | **1/*1* | **1/*1* | **1/*1* |
| NA07019 | **1A/*1F* | **1A/*1F* | **1A/*1F* | **1/*1* | **1/*1* | **1/*1* | **5/*22* | **5/*22* | **5/*22* |
| NA12873 | **1A/*1F* | **1A/*1F* | **1A/*1F* | **1/*9* | **1/*9* | **1/*9* | **1/*6* | **1/*6* | **1/*6* |
| NA19143 | **1A/*1F* | **1A/*1F* | **1A/*1F* | **1/*35* | **1/*1* | **1/*1* | **6/*6* | **6/*6* | **6/*6* |
| NA10847 | **1F/*1F* | **1F/*1F* | *N/A* | **1/*1* | **1/*1* | *N/A* | **1/*6* | **1/*6* | *N/A* |
| NA18518 | **1A/*1A* | **1A/*1A* | **1A/*1A* | **1/*17* | **1/*17* | **1/*17* | **1/*6* | **1/*6* | **1/*6* |
| NA21781 | **1F/*1F* | **1F/*1F* | **1F/*1F* | **1/*21* | **1/*21* | **1/*21* | **1/*4* | **1/*4* | **1/*4* |
| NA07348 | **1F/*1F* | **1F/*1F* | **1F/*1F* | **1/*1* | **1/*1* | **1/*1* | **1/*1* | **1/*1* | **1/*1* |
| NA19003 | **1A/*1F* | **1A/*1F* | **1A/*1F* | **1/*1* | **1/*1* | **1/*1* | **1/*6* | **1/*6* | **1/*6* |
| NA18544 | **1F/*1L* | **1F/*1L* | **1F/*1F [N]* | **7/*18* | **7/*18* | **7/*18* | **1/*1* | **1/*1* | **1/*1* |
| NA18540 | **1L/*1L* | **1L/*1L* | **1F/*1F [N]* | **1/*1* | **1/*1* | **1/*1* | **1/*6* | **1/*6* | **1/*6* |
| NA18966 | **1A/*1F* | **1A/*1F* | **1A/*1F* | **1/*4* | **1/*4* | **1/*4* | **1/*6* | **1/*6* | **1/*6* |

|  | ***CYP2C8*** |  |  | ***CYP2C9*** |  |  | ***CYP2C19*** |  |  |
| --- | --- | --- | --- | --- | --- | --- | --- | --- | --- |
| **Coriell ID** | **Previous^2^** | **WGS** | **ClinPharmSeq** | **Previous^1^** | **WGS** | **ClinPharmSeq** | **Previous^1^** | **WGS** | **ClinPharmSeq** |
| NA10831 | **1/*1* | **1/*1* | **1/*1* | **1/*2* | **1/*2* | **1/*2* | **1/*17* | **1/*17* | **1/*17* |
| NA18855 | **1/*1* | **1/*1* | **1/*1* | **1/*9* | **1/*9* | **1/*9* | **2/*27* | **1/*2* | **1/*2* |
| NA18617 | **1/*1* | **1/*1* | **1/*1* | **1/*1* | **1/*1* | **1/*1* | **1/*2* | **1/*2* | **1/*2* |
| NA19908 | **1/*1* | **1/*1* | **1/*1* | **1/*5* | **1/*5* | **1/*5* | **1/*17* | **1/*17* | **1/*17* |
| NA18973 | **1/*1* | **1/*1* | *N/A* | **1/*1* | **1/*1* | *N/A* | **1/*1* | **1/*1* | *N/A* |
| NA12003 | **1/*3* | **1/*3* | **1/*1 [P]* | **1/*2* | **1/*2* | **1/*2* | **1/*1* | **1/*1* | **1/*1* |
| NA18519 | **1/*2* | **1/*2* | **1/*2* | **1/*5* | **1/*5* | **1/*5* | **1/*17* | **1/*17* | **1/*17* |
| HG00276 | **1/*3* | **1/*3* | **1/*3* | **1/*2* | **1/*2* | **1/*2* | **1/*1* | **1/*1* | **1/*1* |
| NA11993 | **1/*1* | **1/*1* | **1/*1* | **1/*1* | **1/*1* | **1/*1* | **1/*1* | **1/*1* | **1/*1* |
| NA19917 | **1/*1* | **1/*16* | **1/*16* | **1/*1* | **1/*1* | **1/*1* | **2/*15* | **2/*15* | **2/*15* |
| NA19920 | **1/*1* | **1/*1* | **1/*1* | **1/*1* | **1/*1* | **1/*1* | **1/*1* | **1/*1* | **1/*1* |
| NA11832 | **1/*1* | **1/*1* | **1/*1* | **1/*3* | **1/*3* | **1/*3* | **1/*2* | **1/*2* | **1/*2* |
| NA07029 | **1/*3* | **1/*3* | *N/A* | **1/*2* | **1/*2* | *N/A* | **8/*17* | **8/*17* | *N/A* |
| NA18868 | **1/*1* | **1/*1* | **1/*1* | **1/*1* | **1/*1* | **1/*1* | **1/*2* | **1/*2* | **1/*2* |
| NA12813 | **1/*1* | **1/*1* | **1/*1* | **1/*3* | **1/*3* | **1/*3* | **1/*17* | **1/*17* | **1/*17* |
| HG00589 | **1/*1* | **1/*1* | **1/*1* | **1/*1* | **1/*1* | **1/*1* | **1/*1* | **1/*1* | **1/*1* |
| NA20296 | **1/*1* | **1/*1* | **1/*1* | **1/*1* | **1/*1* | **1/*1* | **1/*1* | **1/*1* | **1/*1* |
| NA12717 | **1/*1* | **1/*1* | **1/*1* | **1/*1* | **1/*1* | **1/*1* | **2/*2* | **2/*2* | **2/*2* |
| NA07056 | **1/*1* | **1/*1* | **1/*1* | **1/*1* | **1/*1* | **1/*1* | **1/*1* | **1/*1* | **1/*1* |
| NA18484 | **1/*4* | **1/*4* | **1/*4* | **1/*9* | **1/*9* | **1/*9* | **2/*27* | **1/*2* | **1/*2* |
| NA19178 | **1/*1* | **1/*1* | **1/*1* | **5/*9* | **5/*9* | **5/*9* | **6/*27* | **1/*6* | **1/*6* |
| NA18564 | **1/*1* | **1/*1* | *N/A* | **1/*1* | **1/*1* | *N/A* | **2/*3* | **2/*3* | *N/A* |
| NA12145 | **1/*4* | **1/*4* | **1/*4* | **1/*1* | **1/*1* | **1/*1* | **2/*17* | **2/*17* | **2/*17* |
| NA18861 | **1/*1* | **1/*1* | **1/*1* | **1/*1* | **1/*1* | **1/*1* | **1/*1* | **1/*1* | **1/*1* |
| HG00436 | **1/*1* | **1/*1* | **1/*1* | **1/*1* | **1/*1* | **1/*1* | **1/*1* | **1/*1* | **1/*1* |
| NA18552 | **1/*1* | **1/*1* | **1/*1* | **1/*1* | **1/*1* | **1/*1* | **1/*4* | **1/*4* | **1/*4* |
| NA07000 | **1/*1* | **1/*1* | **1/*1* | **1/*1* | **1/*1* | **1/*1* | **1/*17* | **1/*17* | **1/*17* |
| NA12006 | **1/*1* | **1/*1* | **1/*1* | **1/*1* | **1/*1* | **1/*1* | **1/*1* | **1/*1* | **1/*1* |
| NA19007 | **1/*1* | **1/*1* | **1/*1* | **1/*1* | **1/*1* | **1/*1* | **1/*1* | **1/*1* | **1/*1* |
| NA19239 | **1/*2* | **1/*2* | **1/*2* | **1/*1* | **1/*1* | **1/*1* | **13/*17* | **13/*17* | **13/*17* |
| NA12156 | **1/*1* | **1/*15* | **1/*15* | **1/*2* | **1/*2* | **1/*2* | **1/*1* | **1/*1* | **1/*1* |
| NA19147 | **1/*1* | **1/*1* | **1/*1* | **1/*1* | **1/*1* | **1/*1* | **1/*17* | **1/*17* | **1/*17* |
| NA19095 | **1/*2* | **1/*2* | **1/*2* | **1/*1* | **1/*1* | **1/*1* | **1/*1* | **1/*1* | **1/*1* |
| NA10854 | **3/*3* | **3/*3* | **3/*3* | **2/*2* | **2/*2* | **2/*2* | **1/*1* | **1/*1* | **1/*1* |
| NA18980 | **1/*1* | **1/*1* | **1/*1* | **1/*1* | **1/*1* | **1/*1* | **1/*1* | **1/*1* | **1/*1* |
| NA19207 | **1/*2* | **1/*2* | **1/*2* | **1/*1* | **1/*1* | **1/*1* | **2/*17* | **2/*17* | **2/*17* |
| NA18526 | **1/*1* | **1/*1* | **1/*1* | **1/*1* | **1/*1* | **1/*1* | **1/*1* | **1/*1* | **1/*1* |
| NA18959 | **1/*1* | **1/*1* | *N/A* | **1/*3* | **1/*3* | *N/A* | **1/*1* | **1/*1* | *N/A* |
| NA06991 | **1/*1* | **1/*1* | **1/*1* | **1/*1* | **1/*1* | **1/*1* | **1/*1* | **1/*1* | **1/*1* |
| NA19109 | **2/*2* | **2/*2* | **2/*2* | **1/*1* | **1/*1* | **1/*1* | **17/*17* | **17/*17* | **17/*17* |
| NA18952 | **1/*1* | **1/*1* | *N/A* | **1/*1* | **1/*1* | *N/A* | **1/*1* | **1/*1* | *N/A* |
| NA19789 | **1/*3* | **1/*3* | **1/*3* | **1/*2* | **1/*2* | **1/*2* | **1/*1* | **1/*1* | **1/*1* |
| HG01190 | **1/*3* | **1/*3* | **1/*3* | **1/*2* | **1/*61* | **1/*61* | **1/*2* | **1/*2* | **1/*2* |
| NA19122 | **1/*1* | **1/*1* | **1/*1* | **1/*11* | **1/*11* | **1/*11* | **2/*35* | **2/*35* | **2/*35* |
| NA19819 | **1/*2* | **1/*2* | **1/*2* | **1/*1* | **1/*1* | **1/*1* | **1/*17* | **1/*17* | **1/*17* |
| NA19213 | **1/*1* | **1/*17* | **1/*17* | **1/*6* | **1/*6* | **1/*6* | **1/*15* | **1/*39* | **1/*39* |
| NA07055 | **1/*1* | **1/*1* | **1/*1* | **1/*1* | **1/*1* | **1/*1* | **1/*17* | **1/*17* | **1/*17* |
| NA19174 | **1/*1* | **1/*1* | *N/A* | **1/*1* | **1/*1* | *N/A* | **1/*2* | **1/*2* | *N/A* |
| NA20509 | **1/*4* | **1/*4* | **1/*4* | **1/*1* | **1/*1* | **1/*1* | **2/*2* | **2/*2* | **2/*2* |
| NA18992 | **1/*1* | **1/*1* | **1/*1* | **1/*1* | **1/*1* | **1/*1* | **1/*1* | **1/*1* | **1/*1* |
| NA18565 | **1/*1* | **1/*1* | **1/*1* | **1/*1* | **1/*1* | **1/*1* | **1/*1* | **1/*1* | **1/*1* |
| NA18524 | **1/*1* | **1/*1* | **1/*1* | **1/*3* | **1/*3* | **1/*3* | **1/*2* | **1/*2* | **1/*2* |
| NA18942 | **1/*1* | **1/*1* | **1/*1* | **1/*1* | **1/*1* | **1/*1* | **1/*1* | **1/*1* | **1/*1* |
| NA11839 | **1/*3* | **1/*3* | **1/*3* | **2/*3* | **2/*3* | **2/*3* | **1/*1* | **1/*1* | **1/*1* |
| NA10851 | **1/*1* | **1/*1* | **1/*1* | **1/*1* | **1/*1* | **1/*1* | **1/*17* | **1/*17* | **1/*17* |
| NA19176 | **2/*2* | **2/*2* | **2/*2* | **1/*1* | **1/*1* | **1/*1* | **2/*17* | **2/*17* | **2/*17* |
| NA18509 | **1/*1* | **1/*1* | **1/*1* | **1/*1* | **1/*1* | **1/*1* | **2/*2* | **2/*2* | **2/*2* |
| NA19226 | **1/*1* | **1/*1* | **1/*1* | **1/*8* | **1/*8* | **1/*8* | **1/*2* | **1/*2* | **1/*2* |
| NA07357 | **1/*4* | **1/*4* | **1/*4* | **1/*1* | **1/*1* | **1/*1* | **2/*17* | **2/*17* | **2/*17* |
| NA07019 | **1/*1* | **1/*1* | **1/*1* | **1/*1* | **1/*1* | **1/*1* | **1/*17* | **1/*17* | **1/*17* |
| NA12873 | **1/*1* | **1/*1* | **1/*1* | **1/*1* | **1/*1* | **1/*1* | **1/*17* | **1/*17* | **1/*17* |
| NA19143 | **1/*1* | **1/*17* | **1/*17* | **1/*6* | **1/*6* | **1/*6* | **1/*15* | **1/*39* | **1/*39* |
| NA10847 | **1/*1* | **1/*1* | *N/A* | **1/*1* | **1/*1* | *N/A* | **1/*1* | **1/*1* | *N/A* |
| NA18518 | **1/*2* | **1/*2* | **1/*2* | **1/*1* | **1/*1* | **1/*1* | **2/*17* | **2/*17* | **2/*17* |
| NA21781 | **1/*1* | **1/*1* | **1/*1* | **1/*1* | **1/*1* | **1/*1* | **1/*2* | **1/*2* | **1/*2* |
| NA07348 | **1/*4* | **1/*4* | **1/*4* | **1/*1* | **1/*1* | **1/*1* | **2/*17* | **2/*17* | **2/*17* |
| NA19003 | **1/*1* | **1/*1* | **1/*1* | **1/*1* | **1/*1* | **1/*1* | **1/*2* | **1/*2* | **1/*2* |
| NA18544 | **1/*1* | **1/*1* | **1/*1* | **1/*1* | **1/*1* | **1/*1* | **1/*2* | **1/*2* | **1/*2* |
| NA18540 | **1/*1* | **1/*1* | **1/*1* | **1/*1* | **1/*1* | **1/*1* | **1/*2* | **1/*2* | **1/*2* |
| NA18966 | **1/*1* | **1/*1* | **1/*1* | **1/*S1* | **1/*1* | **1/*1* | **1/*1* | **1/*1* | **1/*1* |

|  | ***CYP2D6*** |  |  | ***CYP2E1*** |  |  | ***CYP3A4*** |  |  |
| --- | --- | --- | --- | --- | --- | --- | --- | --- | --- |
| **Coriell ID** | **Previous^3^** | **WGS** | **ClinPharmSeq** | **Previous^1^** | **WGS** | **ClinPharmSeq** | **Previous^1^** | **WGS** | **ClinPharmSeq** |
| NA10831 | **4/*5* | **4/*5* | **4/*5* | **1/*7* | **1/*7* | **1/*7* | **1/*1* | **1/*1* | **1/*1* |
| NA18855 | **1/*5* | **1/*5* | **1/*5* | **7/*7* | **7/*7* | **7/*7* | **1/*1B* | **1/*1* | **1/*1* |
| NA18617 | **36+*10/*36+*10* | **36+*10/*36+*10* | **36+*10/*36+*10* | **7/*7* | **5/*5* | **5/*5* | **1/*1* | **1/*1* | **1/*1* |
| NA19908 | **1/*46* | **1/*46* | **1/*46* | **7x2/*7x2* | **7/*7x3 [X]* | **7/*7x3 [X]* | **1B/*15* | **1/*15* | **1/*15* |
| NA18973 | **1/*21* | **1/*21* | *N/A* | **1/*7* | **1/*7* | *N/A* | **1/*1* | **1/*1* | *N/A* |
| NA12003 | **4/*35* | **4/*35* | **4/*35* | **1/*1* | **1/*1* | **1/*1* | **1/*1B* | **1/*1* | **1/*1* |
| NA18519 | **1/*29* | **29/*106* | **29/*106* | **1/*1* | **1/*1* | **1/*1* | **1B/*1B* | **1/*1* | **1/*1* |
| HG00276 | **4/*5* | **4/*5* | **4/*5* | **1/*1* | **1/*1* | **1/*1* | **1/*2* | **1/*2* | **1/*2* |
| NA11993 | **1/*9* | **1/*9* | **1/*9* | **1/*1* | **1/*1* | **1/*1* | **1/*1* | **1/*1* | **1/*1* |
| NA19917 | **1/*40* | **1/*40* | **1/*40* | **1/*7* | **1/*7* | **1/*7* | **1/*1B* | **1/*1* | **1/*1* |
| NA19920 | **1/*4x2* | **1/*4x2* | **1/*4x2* | **1/*S1, *7, *4* | **1/*S1* | **1/*S1* | **1B/*1B* | **1/*1* | **1/*1* |
| NA11832 | **1/(*68)+*4* | **1/*68+*4* | **1/*68+*4* | **1/*1* | **1/*1* | **1/*1* | **1/*1* | **1/*1* | **1/*1* |
| NA07029 | **1/*35* | **1/*35* | *N/A* | **1/*1* | **1/*1* | *N/A* | **1/*1* | **1/*1* | *N/A* |
| NA18868 | **2/*5* | **2/*5* | **2/*5* | **7/*7* | **7/*7* | **7/*7* | **1B/*1B* | **1/*1* | **1/*1* |
| NA12813 | **2/*4* | **2/*4* | **2/*4* | **1/*1* | **1/*1* | **1/*1* | **1/*1* | **1/*1* | **1/*1* |
| HG00589 | **1/*21* | **1/*21* | **1/*21* | **1/*7, *5* | **1/*5* | **1/*5* | **1/*1* | **1/*1* | **1/*1* |
| NA20296 | **1/*2* | **1/*2* | **1/*2* | **1/*1* | **1/*1* | **1/*1* | **1B/*1B* | **1/*1* | **1/*1* |
| NA12717 | **1/*1* | **1/*1* | **1/*1* | **1/*1* | **1/*1* | **1/*1* | **1B/*22* | **1/*22* | **1/*22* |
| NA07056 | **2/*4* | **2/*4* | **2/*4* | **1/*1* | **1/*1* | **1/*1* | **1/*22* | **1/*22* | **1/*22* |
| NA18484 | **1/*17* | **1/*17* | **1/*17* | **1/*7* | **1/*7* | **1/*7* | **1B/*1B* | **1/*1* | **1/*1* |
| NA19178 | **1/*1* | **1/*1* | **1/*1* | **7/*7* | **7/*7* | **7/*7* | **1B/*1B* | **1/*1* | **1/*1* |
| NA18564 | **2/*36+*10* | **2/*36+*10* | *N/A* | **1/*7, *5* | **1/*5* | *N/A* | **1/*1* | **1/*1* | *N/A* |
| NA12145 | **1/*4* | **1/*4* | **1/*4* | **1/*1* | **1/*1* | **1/*1* | **1/*1* | **1/*1* | **1/*1* |
| NA18861 | **5/*29* | **5/*29* | **5/*29* | **7/*7* | **7/*7* | **7/*7* | **1B/*1B* | **1/*1* | **1/*1* |
| HG00436 | **2x2/*71* | **2x2/*71* | **2x2/*71* | **7/*7* | **5/*5* | **5/*5* | **1/*1* | **1/*1* | **1/*1* |
| NA18552 | **1/*14* | **1/*14* | **1/*14* | **5/*7* | **5/*7* | **5/*7* | **1/*1* | **1/*1* | **1/*1* |
| NA07000 | **2 (*35)/*9* | **9/*35* | **9/*35* | **1/*1* | **1/*1* | **1/*1* | **1/*1* | **1/*1* | **1/*1* |
| NA12006 | **4/*41* | **4/*41* | **4/*41* | **1/*1* | **1/*1* | **1/*1* | **1/*3* | **1/*3* | **1/*3* |
| NA19007 | **1/*1* | **1/*1* | **1/*1* | **1/*7, *5* | **1/*5* | **1/*5* | **1/*1* | **1/*1* | **1/*1* |
| NA19239 | **15/*17* | **15/*17* | **15/*17* | **7/*7* | **7/*7* | **7/*7* | **1/*1B* | **1/*1* | **1/*1* |
| NA12156 | **1/*4* | **1/*4* | **1/*4* | **1/*1* | **1/*1* | **1/*1* | **1/*1* | **1/*1* | **1/*1* |
| NA19147 | **17/*29* | **17/*29* | **17/*29* | **1/*S1, *7, *4* | **1/*S1* | **1/*S1* | **1/*1B* | **1/*1* | **1/*1* |
| NA19095 | **1/*29* | **1/*29* | **1/*29* | **1/*7x2* | **1/*7x2* | **1/*7x2* | **1/*1B* | **1/*1* | **1/*1* |
| NA10854 | **1/*4* | **1/*4* | **1/*4* | **1/*1* | **1/*1* | **1/*1* | **1/*1B* | **1/*1* | **1/*1* |
| NA18980 | **2/*36+*10* | **2/*36+*10* | **2/*36+*10* | **7/*7* | **5/*5* | **5/*5* | **1/*1* | **1/*1* | **1/*1* |
| NA19207 | **2x2/*10* | **2x2/*10* | **2x2/*10* | **7/*7* | **7/*7* | **7/*7* | **1B/*1B* | **1/*1* | **1/*1* |
| NA18526 | **1/*36x2+*10* | **1/*36x2+*10* | **1/*36x2+*10* | **1/*1* | **1/*1* | **1/*1* | **1/*1* | **1/*1* | **1/*1* |
| NA18959 | **2/*36+*10* | **2/*36+*10* | *N/A* | **1/*1* | **1/*1* | *N/A* | **1/*1* | **1/*1* | *N/A* |
| NA06991 | **1/*4* | **1/*4* | **1/*4* | **1/*1* | **1/*1* | **1/*1* | **1/*1* | **1/*1* | **1/*1* |
| NA19109 | **2x2/*29* | **2x2/*29* | **2x2/*29* | **1/*1* | **1/*1* | **1/*1* | **1B/*15* | **1/*15* | **1/*15* |
| NA18952 | **2/*2* | **2/*2* | *N/A* | **1/*1* | **1/*1* | *N/A* | **1/*1* | **1/*1* | *N/A* |
| NA19789 | **1/*1* | **1/*1* | **1/*1* | **1/*1* | **1/*1* | **1/*1* | **1/*1* | **1/*1* | **1/*1* |
| HG01190 | **68+*4/*5* | **5/*68+*4* | **5/*68+*4* | **1/*7* | **1/*7* | **1/*7* | **1/*1B* | **1/*1* | **1/*1* |
| NA19122 | **2/*17* | **2/*17* | **2/*17* | **7/*7* | **7/*7* | **7/*7* | **1B/*1B* | **1/*1* | **1/*1* |
| NA19819 | **2/*4x2* | **2/*4x2* | **2/*4x2* | **1/*7* | **1/*7* | **1/*7* | **1/*1B* | **1/*1* | **1/*1* |
| NA19213 | **1/*1* | **1/*1* | **1/*1* | **7/*7* | **7/*7* | **7/*7* | **1B/*1B* | **1/*1* | **1/*1* |
| NA07055 | **4/*4* | **4/*4* | **4/*4* | **1/*1* | **1/*1* | **1/*1* | **1/*1* | **1/*1* | **1/*1* |
| NA19174 | **4/*40* | **4/*40* | *N/A* | **7/*7* | **7/*7* | *N/A* | **1B/*1B* | **1/*1* | *N/A* |
| NA20509 | **4/*35* | **4/*35* | **4/*35* | **1/*1* | **1/*1* | **1/*1* | **1/*1* | **1/*1* | **1/*1* |
| NA18992 | **1/*5* | **1/*5* | **1/*5* | **7/*7* | **5/*7* | **5/*7* | **1/*1* | **1/*1* | **1/*1* |
| NA18565 | **10/*36x2* | **10/*36+*10 [X]* | **10/*36+*10 [X]* | **1/*1* | **1/*1* | **1/*1* | **1/*1* | **1/*1* | **1/*1* |
| NA18524 | **1/*36x2+*10* | **1/*36x2+*10* | **1/*36x2+*10* | **7/*7* | **5/*7* | **5/*7* | **1/*1* | **1/*1* | **1/*1* |
| NA18942 | **2/*2* | **2/*2* | **2/*2* | **1/*7, *5* | **1/*5* | **1/*5* | **1/*1* | **1/*1* | **1/*1* |
| NA11839 | **1/*2* | **1/*2* | **1/*2* | **1/*7, *5* | **1/*5* | **1/*5* | **1/*1B* | **1/*1* | **1/*1* |
| NA10851 | **1/*4* | **1/*4* | **1/*4* | **1/*1* | **1/*1* | **1/*1* | **1/*1* | **1/*1* | **1/*1* |
| NA19176 | **1/*2* | **1/*2* | **1/*2* | **7/*7* | **7/*7* | **7/*7* | **1B/*1B* | **1/*1* | **1/*1* |
| NA18509 | **2/*17* | **2/*17* | **2/*17* | **7/*S1* | **7/*S1* | **7/*S1* | **1/*1B* | **1/*1* | **1/*1* |
| NA19226 | **2/*2x2* | **2/*2x2* | **2/*2x2* | **1/*7x2* | **1/*7x2* | **1/*7x2* | **1B/*15* | **1/*15* | **1/*15* |
| NA07357 | **1/*6* | **1/*6* | **1/*6* | **7/*7* | **5/*7* | **5/*7* | **1/*1* | **1/*1* | **1/*1* |
| NA07019 | **1/*4* | **1/*4* | **1/*4* | **1/*1* | **1/*1* | **1/*1* | **1/*1* | **1/*1* | **1/*1* |
| NA12873 | **1/*5* | **1/*5* | **1/*5* | **1/*1* | **1/*1* | **1/*1* | **1/*1* | **1/*1* | **1/*1* |
| NA19143 | **2 (*45)/*10* | **10/*45* | **10/*45* | **7/*S1* | **7/*S1* | **7/*S1* | **1B/*1B* | **1/*1* | **1/*1* |
| NA10847 | **1/*41* | **1/*41* | *N/A* | **1/*1* | **1/*1* | *N/A* | **1/*1* | **1/*1* | *N/A* |
| NA18518 | **17/*29* | **17/*29* | **17/*29* | **1/*7, *4* | **1/*7* | **1/*7* | **1B/*1B* | **1/*1* | **1/*1* |
| NA21781 | **2x2/*68+*4* | **2x2/*68+*4* | **2x2/*68+*4* | **1/*1* | **1/*1* | **1/*1* | **1/*1* | **1/*1* | **1/*1* |
| NA07348 | **1/*6* | **1/*6* | **1/*6* | **1/*7* | **1/*7* | **1/*7* | **1/*1* | **1/*1* | **1/*1* |
| NA19003 | **1/*1* | **1/*1* | **1/*1* | **1/*7* | **1/*7* | **1/*7* | **1/*1* | **1/*1* | **1/*1* |
| NA18544 | **10/*41* | **10/*41* | **10/*41* | **7/*7* | **7/*7* | **7/*7* | **1/*1* | **1/*1* | **1/*1* |
| NA18540 | *(*36+)10/*41* | **36x2+*10/*41 [X]* | **36x2+*10/*41 [X]* | **1/*7, *5* | **1/*5* | **1/*5* | **1/*1/*1* | **1/*1* | **1/*1* |
| NA18966 | **1/*2* | **1/*2* | **1/*2* | **1/*1* | **1/*1* | **1/*1* | **1/*16* | **1/*16* | **1/*16* |

|  | ***CYP3A5*** |  |  | ***CYP4F2*** |  |  | ***DPYD*** |  |  |
| --- | --- | --- | --- | --- | --- | --- | --- | --- | --- |
| **Coriell ID** | **Previous^1^** | **WGS** | **ClinPharmSeq** | **Previous^1^** | **WGS** | **ClinPharmSeq** | **Previous^1^** | **WGS** | **ClinPharmSeq** |
| NA10831 | **3/*3* | **3/*3* | **3/*3* | **1/*1* | **1/*1* | **1/*1* | **5/*9* | *c.85T>C (*9A)/c.1627A>G (*5)* | *c.85T>C (*9A)/c.1627A>G (*5)* |
| NA18855 | **3/*6* | **3/*6* | **3/*6* | **1/*1* | **1/*1* | **1/*1* | **9/*9* | *c.1218G>A/c.85T>C (*9A)* | *c.1218G>A/c.85T>C (*9A)* |
| NA18617 | **3/*3* | **3/*3* | **3/*3* | **1/*1* | **1/*1* | **1/*1* | **1/*1* | *Reference/Reference* | *Reference/Reference* |
| NA19908 | **1/*3* | **1/*3* | **1/*3* | **2/*3* | **2/*3* | **2/*3* | **1/*9* | *Reference/c.1218G>A* | *Reference/c.1218G>A* |
| NA18973 | **1/*3* | **1/*3* | *N/A* | **1/*1* | **1/*1* | *N/A* | **5/*9* | *c.1627A>G (*5)/c.1627A>G (*5)* | *N/A* |
| NA12003 | **1/*3* | **1/*3* | **1/*3* | **1/*1* | **1/*1* | **1/*1* | **1/*1* | *Reference/Reference* | *Reference/Reference* |
| NA18519 | **1/*6* | **1/*6* | **1/*6* | **1/*3, *2* | **1/*2* | **1/*2* | **9/*9* | *c.85T>C (*9A)/c.85T>C (*9A)* | *c.85T>C (*9A)/c.85T>C (*9A)* |
| HG00276 | **3/*3* | **3/*3* | **3/*3* | **1/*1* | **1/*1* | **1/*1* | **1/*1* | *Reference/Reference* | *Reference/Reference* |
| NA11993 | **3/*3* | **3/*3* | **3/*3* | **1/*3* | **1/*3* | **1/*3* | **1/*5* | *c.1682G>T/c.1627A>G (*5)* | *Reference/c.1627A>G (*5)* |
| NA19917 | **1/*7* | **1/*7* | **1/*7* | **1/*2* | **1/*2* | **1/*2* | **9/*9* | *c.85T>C (*9A)/c.85T>C (*9A)* | *c.85T>C (*9A)/c.85T>C (*9A)* |
| NA19920 | **7/*7* | **7/*7* | **7/*7* | **1/*1* | **1/*1* | **1/*1* | **9/*9* | *c.85T>C (*9A)/c.85T>C (*9A)* | *c.85T>C (*9A)/c.85T>C (*9A)* |
| NA11832 | **3/*3* | **3/*3* | **3/*3* | **1/*1* | **1/*1* | **1/*1* | **1/*5* | *c.496A>G/c.1627A>G (*5)* | *c.496A>G/c.1627A>G (*5)* |
| NA07029 | **1/*3* | **1/*3* | *N/A* | **3/*3* | **2/*3* | *N/A* | **1/*1* | *Reference/Reference* | *N/A* |
| NA18868 | **1/*3* | **1/*3* | **1/*3* | **1/*1* | **1/*1* | **1/*1* | **1/*9* | *Reference/c.85T>C (*9A)* | *Reference/c.85T>C (*9A)* |
| NA12813 | **3/*3* | **3/*3* | **3/*3* | **1/*3* | **1/*3* | **1/*3* | **1/*4* | *Reference/c.1601G>A (*4)* | *Reference/c.1601G>A (*4)* |
| HG00589 | **3/*3* | **3/*3* | **3/*3* | **1/*1* | **1/*1* | **1/*1* | **1/*5* | *Reference/c.1627A>G (*5)* | *Reference/c.1627A>G (*5)* |
| NA20296 | **1/*6* | **1/*6* | **1/*6* | **1/*1* | **1/*1* | **1/*1* | **1/*9* | *Reference/c.85T>C (*9A)* | *Reference/c.85T>C (*9A)* |
| NA12717 | **1/*3* | **1/*3* | **1/*3* | **1/*3* | **1/*3* | **1/*3* | **1/*1* | *Reference/Reference* | *Reference/Reference* |
| NA07056 | **3/*3* | **3/*3* | **3/*3* | **3/*3* | **2/*3* | **2/*3* | **1/*1* | *Reference/Reference* | *Reference/Reference* |
| NA18484 | **1/*7* | **1/*7* | **1/*7* | **1/*2* | **1/*2* | **1/*2* | **1/*9* | *Reference/c.85T>C (*9A)* | *Reference/c.85T>C (*9A)* |
| NA19178 | **1/*1* | **1/*1* | **1/*1* | **1/*1* | **1/*1* | **1/*1* | **1/*9* | *Reference/c.1218G>A* | *Reference/c.1218G>A* |
| NA18564 | **1/*1* | **1/*1* | *N/A* | **3/*3* | **2/*3* | *N/A* | **1/*9* | *Reference/c.1627A>G (*5)* | *N/A* |
| NA12145 | **3/*3* | **3/*3* | **3/*3* | **1/*1* | **1/*1* | **1/*1* | **1/*9* | *Reference/c.85T>C (*9A)* | *Reference/c.85T>C (*9A)* |
| NA18861 | **1/*1* | **1/*1* | **1/*1* | **1/*1* | **1/*1* | **1/*1* | **1/*9* | *Reference/c.85T>C (*9A)* | *Reference/c.85T>C (*9A)* |
| HG00436 | **3/*3* | **3/*3* | **3/*3* | **1/*3, *2* | **1/*2* | **1/*2* | **1/*1* | *Reference/Reference* | *Reference/Reference* |
| NA18552 | **3/*3* | **3/*3* | **3/*3* | **1/*3* | **1/*3* | **1/*3* | **1/*5* | *Reference/c.1627A>G (*5)* | *Reference/c.1627A>G (*5)* |
| NA07000 | **1/*3* | **1/*3* | **1/*3* | **3/*3* | **2/*3* | **2/*3* | **1/*1* | *Reference/Reference* | *Reference/Reference* |
| NA12006 | **3/*3* | **3/*3* | **3/*3* | **1/*3* | **1/*3* | **1/*3* | **1/*1* | *Reference/Reference* | *Reference/Reference* |
| NA19007 | **3/*3* | **3/*3* | **3/*3* | **1/*3* | **1/*3* | **1/*3* | **1/*1* | *Reference/Reference* | *Reference/Reference* |
| NA19239 | **1/*1* | **1/*1* | **1/*1* | **1/*1* | **1/*1* | **1/*1* | **1/*9* | *c.1349C>T/c.85T>C (*9A)* | *Reference/c.1349C>T* |
| NA12156 | **3/*3* | **3/*3* | **3/*3* | **1/*3, *2* | **1/*2* | **1/*2* | **1/*5* | *Reference/c.1627A>G (*5)* | *Reference/c.1627A>G (*5)* |
| NA19147 | **1/*3* | **1/*3* | **1/*3* | **1/*1* | **1/*1* | **1/*1* | **1/*9* | *Reference/c.1349C>T* | *Reference/c.1349C>T* |
| NA19095 | **1/*3* | **1/*3* | **1/*3* | **1/*3* | **1/*3* | **1/*3* | **9/*9* | *c.1218G>A/c.85T>C (*9A)* | *c.1218G>A/c.85T>C (*9A)* |
| NA10854 | **1/*3* | **1/*3* | **1/*3* | **1/*3* | **1/*3* | **1/*3* | **1/*1* | *Reference/Reference* | *Reference/Reference* |
| NA18980 | **1/*3* | **1/*3* | **1/*3* | **1/*1* | **1/*1* | **1/*1* | **1/*5* | *Reference/c.1627A>G (*5)* | *Reference/c.1627A>G (*5)* |
| NA19207 | **3/*7* | **3/*7* | **3/*7* | **1/*1* | **1/*1* | **1/*1* | **1/*9* | *c.557A>G/Reference* | *c.557A>G/Reference* |
| NA18526 | **1/*1* | **1/*1* | **1/*1* | **1/*3* | **1/*3* | **1/*3* | **1/*1* | *Reference/Reference* | *Reference/Reference* |
| NA18959 | **1/*3* | **1/*3* | *N/A* | **1/*1* | **1/*1* | *N/A* | **1/*1* | *Reference/Reference* | *N/A* |
| NA06991 | **3/*3* | **3/*3* | **3/*3* | **1/*1* | **1/*1* | **1/*1* | **1/*4* | *c.2846A>T/c.1601G>A (*4)* | *Reference/c.2846A>T* |
| NA19109 | **1/*3* | **1/*3* | **1/*3* | **1/*1* | **1/*1* | **1/*1* | **1/*9* | *Reference/c.1218G>A* | *Reference/c.1218G>A* |
| NA18952 | **3/*3* | **3/*3* | *N/A* | **1/*1* | **1/*1* | *N/A* | **5/*5* | *c.1627A>G (*5)/c.1627A>G (*5)* | *N/A* |
| NA19789 | **3/*3* | **3/*3* | **3/*3* | **1/*3, *2* | **1/*2* | **1/*2* | **1/*5* | *Reference/c.1627A>G (*5)* | *Reference/c.1627A>G (*5)* |
| HG01190 | **1/*1* | **1/*1* | **1/*1* | **1/*3* | **1/*3* | **1/*3* | **1/*9* | *Reference/c.85T>C (*9A)* | *Reference/c.85T>C (*9A)* |
| NA19122 | **1/*1* | **1/*1* | **1/*1* | **1/*2* | **1/*2* | **1/*2* | **1/*1* | *Reference/Reference* | *Reference/Reference* |
| NA19819 | **3/*6* | **3/*6* | **3/*6* | **1/*3, *2* | **1/*2* | **1/*2* | **1/*9* | *Reference/c.1627A>G (*5)* | *Reference/c.1627A>G (*5)* |
| NA19213 | **1/*6* | **1/*6* | **1/*6* | **1/*1* | **1/*1* | **1/*1* | **1/*1* | *Reference/Reference* | *Reference/Reference* |
| NA07055 | **3/*3* | **3/*3* | **3/*3* | **1/*1* | **1/*1* | **1/*1* | **1/*5* | *c.2846A>T/c.1627A>G (*5)* | *Reference/c.2846A>T* |
| NA19174 | **1/*6* | **1/*6* | *N/A* | **1/*1* | **1/*1* | *N/A* | **1/*1* | *Reference/Reference* | *N/A* |
| NA20509 | **3/*3* | **3/*3* | **3/*3* | **1/*1* | **1/*1* | **1/*1* | **1/*9* | *Reference/c.85T>C (*9A)* | *Reference/c.85T>C (*9A)* |
| NA18992 | **3/*3* | **3/*3* | **3/*3* | **1/*1* | **1/*1* | **1/*1* | **1/*1* | *Reference/Reference* | *Reference/Reference* |
| NA18565 | **1/*3* | **1/*3* | **1/*3* | **1/*1* | **1/*1* | **1/*1* | **1/*1* | *Reference/Reference* | *Reference/Reference* |
| NA18524 | **1/*3* | **1/*3* | **1/*3* | **1/*3* | **1/*3* | **1/*3* | **1/*5* | *Reference/c.1627A>G (*5)* | *Reference/c.1627A>G (*5)* |
| NA18942 | **3/*3* | **3/*3* | **3/*3* | **1/*1* | **1/*1* | **1/*1* | **1/*1* | *Reference/Reference* | *Reference/Reference* |
| NA11839 | **1/*3* | **1/*3* | **1/*3* | **1/*3* | **1/*3* | **1/*3* | **1/*5* | *Reference/c.1627A>G (*5)* | *Reference/c.1627A>G (*5)* |
| NA10851 | **3/*3* | **3/*3* | **3/*3* | **1/*1* | **1/*1* | **1/*1* | **1/*9* | *c.496A>G/Reference* | *c.496A>G/Reference* |
| NA19176 | **1/*3* | **1/*3* | **1/*3* | **1/*1* | **1/*1* | **1/*1* | **1/*1* | *Reference/Reference* | *Reference/Reference* |
| NA18509 | **1/*7* | **1/*7* | **1/*7* | **1/*3, *2* | **1/*2* | **1/*3 [X]* | **9/*9* | *c.85T>C (*9A)/c.1627A>G (*5)* | *c.85T>C (*9A)/c.1627A>G (*5)* |
| NA19226 | **1/*6* | **1/*6* | **1/*6* | **1/*2* | **1/*2* | **1/*2* | **1/*9* | *Reference/c.1218G>A* | *Reference/c.1218G>A* |
| NA07357 | **3/*3* | **3/*3* | **3/*3* | **1/*1* | **1/*1* | **1/*1* | **5/*6* | *c.1627A>G (*5)/c.2194G>A (*6)* | *c.1627A>G (*5)/c.2194G>A (*6)* |
| NA07019 | **3/*3* | **3/*3* | **3/*3* | **1/*3* | **1/*3* | **1/*3* | **1/*1* | *Reference/Reference* | *Reference/Reference* |
| NA12873 | **3/*3* | **3/*3* | **3/*3* | **1/*1* | **1/*1* | **1/*1* | **1/*1* | *Reference/Reference* | *Reference/Reference* |
| NA19143 | **6/*7* | **6/*7* | **6/*7* | **1/*1* | **1/*1* | **1/*1* | **1/*1* | *Reference/Reference* | *Reference/Reference* |
| NA10847 | **3/*3* | **3/*3* | *N/A* | **1/*1* | **1/*1* | *N/A* | **1/*9* | *Reference/c.1627A>G (*5)* | *N/A* |
| NA18518 | **1/*6* | **1/*6* | **1/*6* | **1/*2* | **1/*2* | **1/*2* | **5/*9* | *c.85T>C (*9A)/c.1627A>G (*5)* | *c.85T>C (*9A)/c.1627A>G (*5)* |
| NA21781 | **3/*3* | **3/*3* | **3/*3* | **1/*1* | **1/*1* | **1/*1* | **1/*5* | *Reference/c.1627A>G (*5)* | *Reference/c.1627A>G (*5)* |
| NA07348 | **3/*3* | **3/*3* | **3/*3* | **1/*1* | **1/*1* | **1/*1* | **1/*6* | *c.85T>C (*9A)/c.2194G>A (*6)* | *c.1896T>C/c.2194G>A (*6)* |
| NA19003 | **3/*3* | **3/*3* | **3/*3* | **1/*1* | **1/*1* | **1/*1* | **1/*1* | *Reference/Reference* | *Reference/Reference* |
| NA18544 | **1/*3* | **1/*3* | **1/*3* | **1/*1* | **1/*1* | **1/*1* | **1/*5* | *Reference/c.1627A>G (*5)* | *Reference/c.1627A>G (*5)* |
| NA18540 | **1/*1/*3* | **1/*3* | **1/*3* | **1/*3* | **1/*3* | **1/*3* | **1/*9* | *c.496A>G/Reference* | *c.496A>G/Reference* |
| NA18966 | **1/*3* | **1/*3* | **1/*3* | **1/*3* | **1/*3* | **1/*3* | **1/*1* | *Reference/Reference* | *Reference/Reference* |

|  | ***GSTM1*** |  |  | ***GSTP1*** |  |  | ***GSTT1*** |  |  |
| --- | --- | --- | --- | --- | --- | --- | --- | --- | --- |
| **Coriell ID** | **Previous^1^** | **WGS** | **ClinPharmSeq** | **Previous^1^** | **WGS** | **ClinPharmSeq** | **Previous^1^** | **WGS** | **ClinPharmSeq** |
| NA10831 | **0/*0* | **0/*0* | **0/*0* | **A/*A* | **A/*A* | **A/*A* | **A/*A* | **A/*A* | **A/*A* |
| NA18855 | **A/*0* | **0/*A* | **0/*A* | **A/*B* | **A/*B* | **A/*B* | **A/*A* | **A/*A* | **A/*A* |
| NA18617 | **0/*0* | **0/*0* | **0/*0* | **A/*A* | **A/*A* | **A/*A* | **A/*0* | **0/*A* | **0/*A* |
| NA19908 | **A/*Ax2* | **A/*Ax2* | **A/*Ax2* | **B/*C* | **B/*C* | **B/*C* | **A/*0* | **0/*A* | **0/*A* |
| NA18973 | **A/*B* | **A/*B* | *N/A* | **A/*A* | **A/*A* | *N/A* | **A/*0* | **0/*A* | *N/A* |
| NA12003 | **0/*0* | **0/*0* | **0/*0* | **A/*A* | **A/*A* | **A/*A* | **A/*0* | **0/*A* | **0/*A* |
| NA18519 | **A/*A* | **A/*A* | **A/*A* | **A/*A* | **A/*A* | **A/*A* | **A/*0* | **0/*A* | **0/*A* |
| HG00276 | **0/*0* | **0/*0* | **0/*0* | **A/*B* | **A/*B* | **A/*B* | **A/*0* | **0/*A* | **0/*A* |
| NA11993 | **0/*0* | **0/*0* | **0/*0* | **A/*B* | **A/*B* | **A/*B* | **A/*0* | **0/*A* | **0/*A* |
| NA19917 | **A/*A* | **A/*A* | **A/*A* | **A/*A* | **A/*A* | **A/*A* | **A/*0* | **0/*A* | **0/*A* |
| NA19920 | **A/*0* | **0/*A* | **0/*A* | **A/*B* | **A/*B* | **A/*B* | **A/*0* | **0/*A* | **0/*A* |
| NA11832 | **0/*0* | **0/*0* | **0/*0* | **A/*B* | **A/*B* | **A/*B* | **0/*0* | **0/*0* | **0/*0* |
| NA07029 | **A/*0* | **0/*A* | *N/A* | **A/*A* | **A/*A* | *N/A* | **A/*0* | **0/*A* | *N/A* |
| NA18868 | **A/*A* | **A/*A* | **A/*A* | **A/*A* | **A/*A* | **A/*A* | **A/*0* | **0/*A* | **0/*A* |
| NA12813 | **0/*0* | **0/*0* | **0/*0* | **A/*A* | **A/*A* | **A/*A* | **A/*0* | **0/*A* | **0/*A* |
| HG00589 | **0/*0* | **0/*0* | **0/*0* | **A/*A* | **A/*A* | **A/*A* | **0/*0* | **0/*0* | **0/*0* |
| NA20296 | **A/*0* | **0/*A* | **0/*A* | **B/*B* | **B/*B* | **B/*B* | **A/*A* | **A/*A* | **A/*A* |
| NA12717 | **A/*0* | **0/*A* | **0/*A* | **A/*B* | **A/*B* | **A/*B* | **A/*A* | **A/*A* | **A/*A* |
| NA07056 | **0/*0* | **0/*0* | **0/*0* | **A/*B* | **A/*B* | **A/*B* | **0/*0* | **0/*0* | **0/*0* |
| NA18484 | **A/*0* | **0/*A* | **0/*A* | **B/*B* | **B/*B* | **B/*B* | **A/*0* | **0/*A* | **0/*A* |
| NA19178 | **A/*A* | **A/*A* | **A/*A* | **A/*B* | **A/*B* | **A/*B* | **0/*0* | **0/*0* | **0/*0* |
| NA18564 | **0/*0* | **0/*0* | *N/A* | **A/*A* | **A/*A* | *N/A* | **A/*0* | **0/*A* | *N/A* |
| NA12145 | **0/*0* | **0/*0* | **0/*0* | **A/*C* | **A/*C* | **A/*C* | **A/*0* | **0/*A* | **0/*A* |
| NA18861 | **A/*B* | **A/*B* | **A/*B* | **A/*B* | **A/*B* | **A/*B* | **A/*0* | **0/*A* | **0/*A* |
| HG00436 | **0/*0* | **0/*0* | **0/*0* | **A/*B* | **A/*B* | **A/*B* | **0/*0* | **0/*0* | **0/*0* |
| NA18552 | **B/*0* | **0/*B* | **0/*B* | **A/*A* | **A/*A* | **A/*A* | **A/*0* | **0/*A* | **0/*A* |
| NA07000 | **A/*A* | **A/*A* | **A/*A* | **A/*A* | **A/*A* | **A/*A* | **A/*0* | **0/*A* | **0/*A* |
| NA12006 | **A/*0* | **0/*A* | **0/*A* | **A/*A* | **A/*A* | **A/*A* | **A/*A* | **A/*A* | **A/*A* |
| NA19007 | **0/*0* | **0/*0* | **0/*0* | **A/*A* | **A/*A* | **A/*A* | **0/*0* | **0/*0* | **0/*0* |
| NA19239 | **A/*A* | **A/*A* | **A/*A* | **A/*A* | **A/*A* | **A/*A* | **0/*0* | **0/*0* | **0/*0* |
| NA12156 | **0/*0* | **0/*0* | **0/*0* | **A/*B* | **A/*B* | **A/*B* | **A/*A* | **A/*A* | **A/*A* |
| NA19147 | **A/*B* | **A/*B* | **A/*B* | **A/*B* | **A/*B* | **A/*B* | **A/*0* | **0/*A* | **0/*A* |
| NA19095 | **A/*A* | **A/*A* | **A/*A* | **A/*B* | **A/*B* | **A/*B* | **A/*0* | **0/*A* | **0/*A* |
| NA10854 | **0/*0* | **0/*0* | **0/*0* | **A/*C* | **A/*C* | **A/*C* | **A/*A* | **A/*A* | **A/*A* |
| NA18980 | **A/*0* | **0/*A* | **0/*A* | **A/*A* | **A/*A* | **A/*A* | **A/*0* | **0/*A* | **0/*A* |
| NA19207 | **0/*0* | **0/*0* | **0/*0* | **A/*B* | **A/*B* | **A/*B* | **0/*0* | **0/*0* | **0/*0* |
| NA18526 | **0/*0* | **0/*0* | **0/*0* | **A/*B* | **A/*B* | **A/*B* | **0/*0* | **0/*0* | **0/*0* |
| NA18959 | **B/*B* | **B/*B* | *N/A* | **A/*A* | **A/*A* | *N/A* | **A/*0* | **0/*A* | *N/A* |
| NA06991 | **A/*B* | **A/*B* | **A/*B* | **B/*B* | **B/*B* | **B/*B* | **A/*0* | **0/*A* | **0/*A* |
| NA19109 | **A/*A* | **A/*A* | **A/*A* | **A/*B* | **A/*B* | **A/*B* | **A/*A* | **A/*A* | **A/*A* |
| NA18952 | **0/*0* | **0/*0* | *N/A* | **A/*A* | **A/*A* | *N/A* | **A/*A* | **A/*A* | *N/A* |
| NA19789 | **B/*0* | **0/*B* | **0/*B* | **A/*A* | **A/*A* | **A/*A* | **A/*0* | **0/*A* | **0/*A* |
| HG01190 | **0/*0* | **0/*0* | **0/*0* | **A/*B* | **A/*B* | **A/*B* | **0/*0* | **0/*0* | **0/*0* |
| NA19122 | **A/*0* | **0/*A* | **0/*A* | **A/*B* | **A/*B* | **A/*B* | **A/*0* | **0/*A* | **0/*A* |
| NA19819 | **A/*B* | **A/*B* | **A/*B* | **A/*A* | **A/*A* | **A/*A* | **A/*A* | **A/*A* | **A/*A* |
| NA19213 | **A/*0* | **0/*A* | **0/*A* | **A/*B* | **A/*B* | **A/*B* | **A/*A* | **A/*A* | **A/*A* |
| NA07055 | **A/*0* | **0/*A* | **0/*A* | **A/*A* | **A/*A* | **A/*A* | **A/*A* | **A/*A* | **A/*A* |
| NA19174 | **0/*0* | **0/*0* | *N/A* | **A/*B* | **A/*B* | *N/A* | **A/*A* | **A/*A* | *N/A* |
| NA20509 | **0/*0* | **0/*0* | **0/*0* | **A/*A* | **A/*A* | **A/*A* | **A/*A* | **A/*A* | **A/*A* |
| NA18992 | **B/*0* | **0/*B* | **0/*B* | **A/*A* | **A/*A* | **A/*A* | **A/*0* | **0/*A* | **0/*A* |
| NA18565 | **0/*0* | **0/*0* | **0/*0* | **A/*B* | **A/*B* | **A/*B* | **A/*0* | **0/*A* | **0/*A* |
| NA18524 | **0/*0* | **0/*0* | **0/*0* | **A/*A* | **A/*A* | **A/*A* | **A/*A* | **A/*A* | **A/*A* |
| NA18942 | **B/*0* | **0/*B* | **0/*B* | **A/*A* | **A/*A* | **A/*A* | **0/*0* | **0/*0* | **0/*0* |
| NA11839 | **0/*0* | **0/*0* | **0/*0* | **A/*B* | **A/*B* | **A/*B* | **A/*0* | **0/*A* | **0/*A* |
| NA10851 | **0/*0* | **0/*0* | **0/*0* | **A/*C* | **A/*C* | **A/*C* | **0/*0* | **0/*0* | **0/*0* |
| NA19176 | **A/*0* | **0/*A* | **0/*A* | **A/*B* | **A/*B* | **A/*B* | **0/*0* | **0/*0* | **0/*0* |
| NA18509 | **0/*0* | **0/*0* | **0/*0* | **B/*B* | **B/*B* | **B/*B* | **A/*0* | **0/*A* | **0/*A* |
| NA19226 | **A/*A* | **A/*A* | **A/*A* | **A/*A* | **A/*A* | **A/*A* | **0/*0* | **0/*0* | **0/*0* |
| NA07357 | **0/*0* | **0/*0* | **0/*0* | **A/*A* | **A/*A* | **A/*A* | **0/*0* | **0/*0* | **0/*0* |
| NA07019 | **0/*0* | **0/*0* | **0/*0* | **B/*B* | **B/*B* | **B/*B* | **A/*0* | **0/*A* | **0/*A* |
| NA12873 | **0/*0* | **0/*0* | **0/*0* | **A/*C* | **A/*C* | **A/*C* | **A/*0* | **0/*A* | **0/*A* |
| NA19143 | **A/*0* | **0/*A* | **0/*A* | **A/*B* | **A/*B* | **A/*B* | **0/*0* | **0/*0* | **0/*0* |
| NA10847 | **A/*0* | **0/*A* | *N/A* | **A/*B* | **A/*B* | *N/A* | **B/*0* | **0/*A* | *N/A* |
| NA18518 | **A/*0* | **0/*A* | **0/*A* | **A/*A* | **A/*A* | **A/*A* | **A/*0* | **0/*A* | **0/*A* |
| NA21781 | **0/*0* | **0/*0* | **0/*0* | **A/*A* | **A/*A* | **A/*A* | **0/*0* | **0/*0* | **0/*0* |
| NA07348 | **A/*0* | **0/*A* | **0/*A* | **A/*A* | **A/*A* | **A/*A* | **A/*0* | **0/*A* | **0/*A* |
| NA19003 | **0/*0* | **0/*0* | **0/*0* | **A/*A* | **A/*A* | **A/*A* | **A/*0* | **0/*A* | **0/*A* |
| NA18544 | **B/*0* | **0/*B* | **0/*B* | **B/*B* | **B/*B* | **B/*B* | **A/*0* | **0/*A* | **0/*A* |
| NA18540 | **B/*B* | **B/*B* | **B/*B* | **A/*A* | **A/*A* | **A/*A* | **0/*0* | **0/*0* | **0/*0* |
| NA18966 | **0/*0* | **0/*0* | **0/*0* | **A/*A* | **A/*A* | **A/*A* | **A/*A* | **A/*A* | **A/*A* |

|  | ***NAT1*** |  |  | ***NAT2*** |  |  | ***SLC15A2*** |  |  |
| --- | --- | --- | --- | --- | --- | --- | --- | --- | --- |
| **Coriell ID** | **Previous^2^** | **WGS** | **ClinPharmSeq** | **Previous^1^** | **WGS** | **ClinPharmSeq** | **Previous^2^** | **WGS** | **ClinPharmSeq** |
| NA10831 | **4/*4* | **4/*4* | **4/*4* | **4/*5* | **4/*5* | **4/*5* | **1/*2* | **1/*2* | **1/*2* |
| NA18855 | **4/*11* | **4/*11* | **4/*11* | **6/*13* | **6/*13* | **6/*13* | **1/*1* | **1/*1* | **1/*1* |
| NA18617 | **4/*4* | **4/*4* | **4/*4* | **4/*6* | **4/*6* | **4/*6* | **2/*2* | **2/*2* | **2/*2* |
| NA19908 | **4/*4* | **4/*4* | **4/*4* | **5/*5* | **5/*5* | **5/*5* | **1/*2* | **1/*2* | **1/*2* |
| NA18973 | **4/*4* | **4/*4* | *N/A* | **4/*6* | **4/*6* | *N/A* | **1/*2* | **1/*2* | *N/A* |
| NA12003 | **4/*4* | **4/*4* | **4/*4* | **5/*5* | **5/*5* | **5/*5* | **1/*1* | **1/*1* | **1/*1* |
| NA18519 | **4/*4* | **4/*4* | **4/*4* | **4/*13* | **4/*13* | **4/*13* | **1/*2* | **1/*2* | **1/*2* |
| HG00276 | **4/*4* | **4/*4* | **4/*4* | **5/*6* | **5/*6* | **5/*6* | **1/*1* | **1/*1* | **1/*1* |
| NA11993 | **4/*4* | **4/*4* | **4/*4* | **5/*5* | **5/*5* | **5/*5* | **2/*2* | **2/*2* | **2/*2* |
| NA19917 | **4/*4* | **4/*4* | **4/*4* | **4/*6* | **4/*6* | **4/*6* | **2/*2* | **2/*2* | **2/*2* |
| NA19920 | **4/*4* | **4/*4* | **4/*4* | **6/*6* | **6/*6* | **6/*6* | **2/*2* | **2/*2* | **2/*2* |
| NA11832 | **4/*4* | **4/*4* | **4/*4* | **5/*12* | **5/*12* | **5/*12* | **1/*2* | **1/*2* | **1/*2* |
| NA07029 | **4/*4* | **4/*4* | **4/*4* | **5/*6* | **5/*6* | *N/A* | **1/*2* | **1/*2* | *N/A* |
| NA18868 | **4/*4* | **4/*4* | **4/*4* | **5, *12/*13* | **5/*13* | **5/*13* | **1/*2* | **1/*2* | **1/*2* |
| NA12813 | **4/*4* | **4/*4* | **4/*4* | **5/*6* | **5/*6* | **5/*6* | **1/*2* | **1/*2* | **1/*2* |
| HG00589 | **4/*4* | **4/*4* | **4/*4* | **4/*13* | **4/*13* | **4/*13* | **2/*2* | **2/*2* | **2/*2* |
| NA20296 | **4/*4* | **4/*4* | **4/*4* | **6/*6* | **6/*6* | **6/*6* | **2/*2* | **2/*2* | **2/*2* |
| NA12717 | **4/*4* | **4/*4* | **4/*4* | **4/*5* | **4/*5* | **4/*5* | **2/*2* | **2/*2* | **2/*2* |
| NA07056 | **4/*4* | **4/*4* | **4/*4* | **6/*6* | **6/*6* | **6/*6* | **1/*2* | **1/*2* | **1/*2* |
| NA18484 | **4/*4* | **4/*4* | **4/*4* | **4/*14* | **4/*14* | **4/*14* | **1/*2* | **1/*2* | **1/*2* |
| NA19178 | **4/*4* | **4/*4* | **4/*4* | **5/*6* | **5/*6* | **5/*6* | **2/*2* | **2/*2* | **2/*2* |
| NA18564 | **4/*4* | **4/*4* | **4/*4* | **4/*7* | **4/*7* | *N/A* | **2/*2* | **2/*2* | *N/A* |
| NA12145 | **4/*14* | **4/*14* | **4/*14* | **4/*5* | **4/*5* | **4/*5* | **1/*2* | **1/*2* | **1/*2* |
| NA18861 | **4/*4* | **4/*4* | **4/*4* | **5/*5* | **5/*5* | **5/*5* | **1/*1* | **1/*1* | **1/*1* |
| HG00436 | **4/*4* | **4/*4* | **4/*4* | **7/*7* | **7/*7* | **7/*7* | **2/*2* | **2/*2* | **2/*2* |
| NA18552 | **4/*4* | **4/*4* | **4/*4* | **4/*4* | **4/*4* | **4/*4* | **2/*2* | **2/*2* | **2/*2* |
| NA07000 | **4/*11* | **4/*11* | **4/*11* | **5/*5* | **5/*5* | **5/*5* | **2/*2* | **2/*2* | **2/*2* |
| NA12006 | **4/*11* | **4/*11* | **4/*11* | **6/*6* | **6/*6* | **6/*6* | **1/*2* | **1/*2* | **1/*2* |
| NA19007 | **4/*4* | **4/*4* | **4/*4* | **4/*7* | **4/*7* | **4/*7* | **1/*2* | **1/*2* | **1/*2* |
| NA19239 | **4/*4* | **4/*4* | **4/*4* | **12/*14* | **12/*14* | **12/*14* | **1/*2* | **1/*2* | **1/*2* |
| NA12156 | **4/*4* | **4/*4* | **4/*4* | **5/*6* | **5/*6* | **5/*6* | **2/*2* | **2/*2* | **2/*2* |
| NA19147 | **4/*4* | **4/*4* | **4/*4* | **5/*13* | **5/*13* | **5/*13* | **1/*2* | **1/*2* | **1/*1 [P]* |
| NA19095 | **4/*4* | **4/*4* | **4/*4* | **6/*6* | **6/*6* | **6/*6* | **2/*2* | **2/*2* | **2/*2* |
| NA10854 | **4/*17* | **4/*17* | **4/*17* | **4/*6* | **4/*6* | **4/*6* | **2/*2* | **2/*2* | **2/*2* |
| NA18980 | **4/*4* | **4/*4* | **4/*4* | **4/*7* | **4/*7* | **4/*7* | **2/*2* | **2/*2* | **2/*2* |
| NA19207 | **4/*4* | **4/*4* | **4/*4* | **12/*14* | **12/*14* | **12/*14* | **1/*1* | **1/*1* | **1/*1* |
| NA18526 | **4/*4* | **4/*4* | **4/*4* | **4/*4* | **4/*4* | **4/*4* | **1/*2* | **1/*2* | **1/*2* |
| NA18959 | **4/*4* | **4/*4* | **4/*4* | **6/*7* | **6/*7* | *N/A* | **1/*2* | **1/*2* | *N/A* |
| NA06991 | **4/*11* | **4/*11* | **4/*11* | **5/*6* | **5/*6* | **5/*6* | **1/*1* | **1/*1* | **1/*1* |
| NA19109 | **4/*4* | **4/*4* | **4/*4* | **4/*6* | **4/*6* | **4/*6* | **1/*2* | **1/*2* | **1/*2* |
| NA18952 | **4/*4* | **4/*4* | **4/*4* | **4/*4* | **4/*4* | *N/A* | **2/*2* | **2/*2* | *N/A* |
| NA19789 | **4/*4* | **4/*4* | **4/*4* | **4/*4* | **4/*4* | **4/*4* | **1/*1* | **1/*1* | **1/*1* |
| HG01190 | **4/*4* | **4/*4* | **4/*4* | **4/*4* | **4/*4* | **4/*4* | **1/*1* | **1/*1* | **1/*1* |
| NA19122 | **4/*4* | **4/*4* | **4/*4* | **12/*12* | **12/*24* | **12/*24* | **1/*2* | **1/*2* | **1/*2* |
| NA19819 | **4/*4* | **4/*4* | **4/*4* | **5/*6* | **5/*6* | **5/*6* | **1/*2* | **1/*2* | **1/*2* |
| NA19213 | **4/*4* | **4/*4* | **4/*4* | **12/*14* | **12/*14* | **12/*14* | **1/*2* | **1/*2* | **1/*2* |
| NA07055 | **4/*17* | **4/*17* | **4/*17* | **5/*5* | **5/*5* | **5/*5* | **1/*2* | **1/*2* | **1/*2* |
| NA19174 | **4/*4* | **4/*4* | **4/*4* | **5/*6* | **5/*6* | *N/A* | **1/*1* | **1/*1* | *N/A* |
| NA20509 | **4/*4* | **4/*4* | **4/*4* | **5/*6* | **5/*6* | **5/*6* | **1/*2* | **1/*2* | **1/*2* |
| NA18992 | **4/*4* | **4/*4* | **4/*4* | **4/*4* | **4/*4* | **4/*4* | **1/*2* | **1/*2* | **1/*1 [P]* |
| NA18565 | **4/*4* | **4/*4* | **4/*4* | **4/*7* | **4/*7* | **4/*7* | **1/*2* | **1/*2* | **1/*2* |
| NA18524 | **4/*4* | **4/*4* | **4/*4* | **4/*4* | **4/*4* | **4/*4* | **2/*2* | **2/*2* | **2/*2* |
| NA18942 | **4/*4* | **4/*4* | **4/*4* | **4/*6* | **4/*6* | **4/*6* | **2/*2* | **2/*2* | **2/*2* |
| NA11839 | **4/*4* | **4/*4* | **4/*4* | **5/*6* | **5/*6* | **5/*6* | **1/*2* | **1/*2* | **1/*2* |
| NA10851 | **4/*4* | **4/*4* | **4/*4* | **4/*5* | **4/*5* | **4/*5* | **1/*2* | **1/*2* | **1/*2* |
| NA19176 | **4/*4* | **4/*4* | **4/*4* | **5/*6* | **5/*6* | **5/*6* | **1/*2* | **1/*2* | **1/*2* |
| NA18509 | **4/*4* | **4/*4* | **4/*4* | **12/*13* | **13/*13* | **13/*13* | **1/*2* | **1/*2* | **1/*2* |
| NA19226 | **4/*4* | **4/*4* | **4/*4* | **4/*5* | **4/*5* | **4/*5* | **1/*2* | **1/*2* | **1/*2* |
| NA07357 | **4/*4* | **4/*4* | **4/*4* | **5/*6* | **5/*6* | **5/*6* | **1/*1* | **1/*1* | **1/*1* |
| NA07019 | **4/*4* | **4/*4* | **4/*4* | **6/*6* | **6/*6* | **6/*6* | **1/*1* | **1/*1* | **1/*1* |
| NA12873 | **4/*4* | **4/*4* | **4/*4* | **5/*5* | **5/*5* | **5/*5* | **1/*2* | **1/*2* | **1/*1 [P]* |
| NA19143 | **4/*4* | **4/*4* | **4/*4* | **13/*14* | **13/*14* | **13/*14* | **1/*2* | **1/*2* | **1/*2* |
| NA10847 | **4/*4* | **4/*4* | **4/*4* | **5/*5* | **5/*5* | *N/A* | **1/*2* | **1/*2* | *N/A* |
| NA18518 | **4/*4* | **4/*4* | **4/*4* | **4/*14* | **4/*14* | **4/*14* | **1/*2* | **1/*2* | **1/*2* |
| NA21781 | **4/*4* | **4/*4* | **4/*4* | **5/*6* | **5/*6* | **5/*6* | **1/*2* | **1/*2* | **1/*2* |
| NA07348 | **4/*4* | **4/*4* | **4/*4* | **5/*5* | **5/*5* | **5/*5* | **1/*2* | **1/*2* | **1/*2* |
| NA19003 | **4/*4* | **4/*4* | **4/*4* | **4/*4* | **4/*4* | **4/*4* | **2/*2* | **2/*2* | **2/*2* |
| NA18544 | **4/*4* | **4/*4* | **4/*4* | **4/*6* | **4/*6* | **4/*6* | **2/*2* | **2/*2* | **2/*2* |
| NA18540 | **4/*4* | **4/*4* | **4/*4* | **5/*7* | **5/*7* | **5/*7* | **2/*2* | **2/*2* | **2/*2* |
| NA18966 | **4/*4* | **4/*4* | **4/*4* | **4/*4* | **4/*4* | **4/*4* | **2/*2* | **2/*2* | **2/*2* |

|  | ***SLC22A2*** |  |  | ***SLCO1B1*** |  |  | ***SLCO2B1*** |  |  |
| --- | --- | --- | --- | --- | --- | --- | --- | --- | --- |
| **Coriell ID** | **Previous^1^** | **WGS** | **ClinPharmSeq** | **Previous^1^** | **WGS** | **ClinPharmSeq** | **Previous^1^** | **WGS** | **ClinPharmSeq** |
| NA10831 | **2/*2* | **2/*2* | **2/*2* | **1A/*14* | **1A/*14* | **1A/*14* | **1/*1* | **1/*1* | **1/*1* |
| NA18855 | **2/*S1* | **2/*S1* | **2/*S1* | **1B/*1B* | **1B/*1B* | **1B/*1B* | **1/*1* | **1/*1* | **1/*1* |
| NA18617 | **1/*4* | **1/*4* | **1/*4* | **1B/*1B* | **1B/*1B* | **1B/*1B* | **1/*S464F* | **1/*S464F* | **1/*S464F* |
| NA19908 | **3/*6* | **3/*6* | **3/*6* | **1B/*1B* | **1B/*1B* | **1B/*1B* | **S464F/*S464F* | **S464F/*S464F* | **S464F/*S464F* |
| NA18973 | **1/*1* | **1/*1* | *N/A* | **1A/*1B* | **1A/*1B* | *N/A* | **1/*S464F* | **1/*S464F* | *N/A* |
| NA12003 | **1/*2* | **1/*2* | **1/*2* | **1A/*15* | **1A/*15* | **1A/*15* | **1/*1* | **1/*1* | **1/*1* |
| NA18519 | **1/*3* | **1/*2* | **2/*3 [P]* | **1B/*1B* | **1B/*1B* | **1B/*1B* | **1/*S464F* | **1/*S464F* | **1/*S464F* |
| HG00276 | **2/*3* | **2/*3* | **2/*3* | **1A/*15* | **1A/*15* | **1A/*15* | **1/*1* | **1/*1* | **1/*1* |
| NA11993 | **1/*2* | **1/*2* | **1/*2* | **1A/*17* | **1A/*17* | **1A/*17* | **1/*1* | **1/*1* | **1/*1* |
| NA19917 | **1/*3* | **1/*3* | **1/*3* | **1A/*1B* | **1A/*1B* | **1A/*1B* | **1/*1* | **1/*1* | **1/*1* |
| NA19920 | **2/*3* | **2/*3* | **2/*3* | **1A/*1B* | **1A/*1B* | **1A/*1B* | **1/*S464F* | **1/*S464F* | **1/*S464F* |
| NA11832 | **3/*3* | **3/*3* | **3/*3* | **14/*35* | **14/*20* | **14/*20* | **1/*1* | **1/*1* | **1/*1* |
| NA07029 | **1/*2* | **1/*2* | *N/A* | **1A/*1A* | **1A/*1A* | *N/A* | **1/*1* | **1/*1* | *N/A* |
| NA18868 | **1/*7* | **1/*7* | **1/*7* | **1B/*24* | **1B/*24* | **1B/*24* | **1/*S464F* | **1/*S464F* | **1/*S464F* |
| NA12813 | **2/*2* | **2/*2* | **2/*2* | **1A/*21* | **1A/*20* | **1A/*20* | **S1/*S464F* | **S1/*S464F* | **S1/*S464F* |
| HG00589 | **1/*1* | **1/*1* | **1/*1* | **1B/*S1* | **1B/*S1* | **1B/*S1* | **1/*S1* | **1/*S1* | **1/*S1* |
| NA20296 | **3/*3* | **3/*3* | **3/*3* | **1B/*27* | **1B/*27* | **1B/*27* | **1/*1* | **1/*1* | **1/*1* |
| NA12717 | **2/*3* | **2/*3* | **2/*3* | **1A/*1A* | **1A/*1A* | **1A/*1A* | **1/*S464F* | **1/*S464F* | **1/*S464F* |
| NA07056 | **2/*2* | **2/*2* | **2/*2* | **1A/*14* | **1A/*14* | **1A/*14* | **1/*1* | **1/*1* | **1/*1* |
| NA18484 | **2/*3* | **2/*3* | **2/*3* | **1B/*S2* | **1B/*S2* | **1B/*S2* | **1/*1* | **1/*1* | **1/*1* |
| NA19178 | **1/*2* | **1/*2* | **1/*2* | **1A/*1B* | **1A/*1B* | **1A/*1B* | **1/*1* | **1/*1* | **1/*1* |
| NA18564 | **2/*3* | **2/*3* | *N/A* | **1A/*1B* | **1A/*1B* | *N/A* | **1/*1* | **1/*1* | *N/A* |
| NA12145 | **2/*3* | **2/*3* | **1/*2 [P]* | **1A/*1A* | **1A/*1A* | **1A/*1A* | **1/*1* | **1/*1* | **1/*1* |
| NA18861 | **2/*3* | **2/*2* | **2/*2* | **1A/*14* | **1A/*32* | **1A/*32* | **1/*1* | **1/*1* | **1/*1* |
| HG00436 | **1/*1* | **1/*1* | **1/*1* | **1B/*1B* | **1B/*1B* | **1B/*1B* | **1/*S464F* | **1/*S464F* | **1/*S464F* |
| NA18552 | **2/*3* | **2/*3* | **2/*6* | **1B/*15* | **1B/*15* | **1B/*15* | **1/*1* | **1/*1* | **1/*1* |
| NA07000 | **1/*2* | **1/*2* | **1/*2* | **1A/*15* | **1A/*15* | **1A/*15* | **1/*1* | **1/*1* | **1/*1* |
| NA12006 | **2/*2* | **2/*2* | **2/*2* | **1B/*21* | **1B/*21* | **1B/*21* | **1/*1* | **1/*1* | **1/*1* |
| NA19007 | **1/*2* | **1/*2* | **1/*2* | **1B/*1B* | **1B/*1B* | **1B/*1B* | **1/*S464F* | **1/*S464F* | **1/*S464F* |
| NA19239 | **1/*2* | **1/*2* | **1/*2* | **1B/*1B* | **1B/*1B* | **1B/*1B* | **1/*S464F* | **1/*S464F* | **1/*S464F* |
| NA12156 | **2/*3* | **2/*3* | **2/*3* | **1A/*21* | **1A/*21* | **1A/*21* | **1/*1* | **1/*1* | **1/*1* |
| NA19147 | **1/*3* | **1/*2* | **1/*2* | **30/*35* | **20/*30* | **20/*30* | **1/*S464F* | **1/*S464F* | **1/*S464F* |
| NA19095 | **2/*3* | **2/*3* | **2/*3* | **1B/*35* | **1B/*20* | **1B/*20* | **1/*1* | **1/*1* | **1/*1* |
| NA10854 | **1/*2* | **1/*2* | **1/*2* | **1A/*1B* | **1A/*1B* | **1A/*1B* | **1/*1* | **1/*1* | **1/*1* |
| NA18980 | **1/*2* | **1/*2* | **1/*2* | **1A/*1B* | **1A/*1B* | **1A/*1B* | **S1/*S464F* | **S1/*S464F* | **1/*S464F [P]* |
| NA19207 | **2/*2* | **2/*2* | **2/*2* | **1B/*S2* | **1B/*S2* | **1B/*S2* | **1/*1* | **1/*1* | **1/*1* |
| NA18526 | **1/*4* | **1/*4* | **1/*4* | **1A/*15* | **1A/*15* | **1A/*15* | **1/*1* | **1/*1* | **1/*1* |
| NA18959 | **1/*4* | **1/*4* | *N/A* | **1B/*1B* | **1B/*1B* | *N/A* | **1/*S464F* | **1/*S464F* | *N/A* |
| NA06991 | **2/*3* | **2/*3* | **2/*3* | **15/*15* | **15/*15* | **15/*15* | **1/*1* | **1/*1* | **1/*1* |
| NA19109 | **2/*3* | **2/*3* | **2/*3* | **1B/*15* | **1B/*15* | **1B/*15* | **1/*S464F* | **1/*S464F* | **1/*S464F* |
| NA18952 | **1/*1* | **1/*1* | *N/A* | **1A/*1A* | **1A/*1A* | *N/A* | **1/*S1* | **1/*S1* | *N/A* |
| NA19789 | **1/*3* | **1/*3* | **1/*3* | **1A/*1B* | **1A/*1B* | **1A/*1B* | **1/*1* | **1/*1* | **1/*1* |
| HG01190 | **1/*3* | **1/*3* | **1/*3* | **1A/*1A* | **1A/*1A* | **1A/*1A* | **1/*1* | **1/*1* | **1/*1* |
| NA19122 | **3/*3* | **3/*3* | **3/*3* | **1B/*1B* | **1B/*1B* | **1B/*1B* | **1/*S464F* | **1/*S464F* | **1/*S464F* |
| NA19819 | **3/*S2* | **1/*S2* | **1/*S2* | **1B/*1B* | **1B/*1B* | **1B/*1B* | **1/*1* | **1/*1* | **1/*1* |
| NA19213 | **1/*3* | **1/*3* | **1/*3* | **1B/*14* | **1B/*14* | **1B/*14* | **1/*S464F* | **1/*S464F* | **1/*S464F* |
| NA07055 | **3/*3* | **3/*3* | **3/*3* | **1A/*14* | **1A/*14* | **1A/*14* | **1/*1* | **1/*1* | **1/*1* |
| NA19174 | **2/*2* | **2/*2* | *N/A* | **1A/*27* | **1A/*27* | *N/A* | **1/*S464F* | **1/*S464F* | *N/A* |
| NA20509 | **3/*3* | **3/*3* | **3/*3* | **1A/*15* | **1A/*15* | **1A/*15* | **1/*1* | **1/*1* | **1/*1* |
| NA18992 | **3/*3* | **3/*3* | **3/*3* | **1A/*17* | **1A/*17* | **1A/*17* | **1/*1* | **1/*1* | **1/*1* |
| NA18565 | **3/*3* | **3/*3* | **3/*3* | **1A/*1A* | **1A/*1A* | **1A/*1A* | **1/*S464F* | **1/*S464F* | **1/*S464F* |
| NA18524 | **1/*3* | **1/*3* | **1/*3* | **1B/*21* | **1B/*21* | **1B/*21* | **1/*S1* | **1/*S1* | **1/*S1* |
| NA18942 | **1/*1* | **1/*1* | **1/*1* | **1B/*1B* | **1B/*1B* | **1B/*1B* | **S464F/*S464F* | **S464F/*S464F* | **S464F/*S464F* |
| NA11839 | **2/*2* | **2/*2* | **2/*2* | **1A/*1B* | **1A/*1B* | **1A/*1B* | **1/*1* | **1/*1* | **1/*1* |
| NA10851 | **1/*2* | **1/*2* | **1/*2* | **1A/*14* | **1A/*14* | **1A/*14* | **1/*1* | **1/*1* | **1/*1* |
| NA19176 | **1/*S1* | **1/*S1* | **1/*S1* | **1A/*1B* | **1A/*1B* | **1A/*1B* | **1/*1* | **1/*1* | **1/*1* |
| NA18509 | **7/*S1* | **7/*S1* | **7/*S1* | **1A/*1B* | **1A/*1B* | **1A/*1B* | **1/*S464F* | **1/*S464F* | **1/*S464F* |
| NA19226 | **3/*S2* | **3/*S2* | **3/*S2* | **1B/*31* | **1B/*31* | **1B/*31* | **1/*1* | **1/*1* | **1/*1* |
| NA07357 | **1/*2* | **1/*2* | **1/*2* | **1A/*15* | **1A/*15* | **1A/*15* | **1/*1* | **1/*1* | **1/*1* |
| NA07019 | **2/*3* | **2/*3* | **2/*3* | **1A/*14* | **1A/*14* | **1A/*14* | **1/*1* | **1/*1* | **1/*1* |
| NA12873 | **1/*1* | **1/*1* | **1/*1* | **1A/*1A* | **1A/*1A* | **1A/*1A* | **1/*1* | **1/*1* | **1/*1* |
| NA19143 | **3/*3* | **3/*3* | **3/*3* | **1B/*1B* | **1B/*1B* | **1B/*1B* | **1/*S464F* | **1/*S464F* | **1/*S464F* |
| NA10847 | **1/*2* | **1/*2* | *N/A* | **5/*15* | **5/*15* | *N/A* | **1/*1* | **1/*1* | *N/A* |
| NA18518 | **1/*7* | **1/*7* | **1/*7* | **1A/*1B* | **1A/*1B* | **1A/*1B* | **1/*S464F* | **1/*S464F* | **1/*S464F* |
| NA21781 | **1/*2* | **1/*2* | **1/*2* | **5/*15* | **5/*15* | **5/*15* | **1/*1* | **1/*1* | **1/*1* |
| NA07348 | **2/*3* | **2/*3* | **2/*3* | **1A/*1A* | **1A/*1A* | **1A/*1A* | **1/*1* | **1/*1* | **1/*1* |
| NA19003 | **1/*2* | **1/*2* | **1/*2* | **1B/*1B* | **1B/*1B* | **1B/*1B* | **1/*1* | **1/*1* | **1/*1* |
| NA18544 | **1/*1* | **1/*1* | **1/*1* | **1B/*17* | **1B/*17* | **1B/*17* | **1/*1* | **1/*1* | **1/*1* |
| NA18540 | **1/*3* | **1/*3* | **1/*3* | **1B/*17* | **1B/*17* | **1B/*17* | **S464F/*S464F* | **S464F/*S464F* | **1/*S464F [X]* |
| NA18966 | **1/*2* | **1/*2* | **1/*2* | **1A/*1A* | **1A/*1A* | **1A/*1A* | **S464F/*S464F* | **S464F/*S464F* | **S464F/*S464F* |

|  | ***TPMT*** |  |  | ***UGT1A1*** |  |  | ***UGT2B7*** |  |  |
| --- | --- | --- | --- | --- | --- | --- | --- | --- | --- |
| **Coriell ID** | **Previous^1^** | **WGS** | **ClinPharmSeq** | **Previous^1^** | **WGS** | **ClinPharmSeq** | **Previous^1^** | **WGS** | **ClinPharmSeq** |
| NA10831 | **1/*1* | **1/*1* | **1/*1* | **28, *60/*28, *60* | **80+*28/*80+*28* | **80+*28/*80+*28* | **1/*2* | **1/*2* | **1/*2* |
| NA18855 | **1/*3C* | **1/*3C* | **1/*3C* | **28, *60/*28, *60* | **80+*28/*80+*28* | **80/*80+*28 [X]* | **1/*2* | **1/*2* | **1/*2* |
| NA18617 | **1/*1* | **1/*1* | **1/*1* | **1/*6* | **1/*6* | **1/*6* | **1/*2* | **1/*2* | **1/*2* |
| NA19908 | **1/*1* | **1/*1* | **1/*1* | **28, *60/*60* | **1/*80+*28* | **1/*80+*28* | **1/*1* | **1/*1* | **1/*1* |
| NA18973 | **1/*1* | **1/*1* | *N/A* | **1/*6* | **1/*6* | *N/A* | **1/*3* | **1/*3* | *N/A* |
| NA12003 | **1/*1* | **1/*1* | **1/*1* | **1/*28, *60* | **1/*80+*28* | **1/*80+*28* | **1/*2* | **1/*2* | **1/*2* |
| NA18519 | **1/*1* | **1/*1* | **1/*1* | **28, *60/*60* | **1/*80+*28* | **1/*80+*28* | **1/*2* | **1/*2* | **1/*2* |
| HG00276 | **1/*16* | **1/*16* | **1/*16* | **1/*28, *60* | **1/*80+*28* | **1/*80+*28* | **1/*1* | **1/*1* | **1/*1* |
| NA11993 | **1/*1* | **1/*1* | **1/*1* | **28, *60/*60* | **1/*80+*28* | **1/*80+*28* | **1/*2* | **1/*2* | **1/*2* |
| NA19917 | **1/*1* | **1/*1* | **1/*1* | **1/*28, *60* | **1/*80+*28* | **1/*80+*28* | **1/*1* | **1/*1* | **1/*1* |
| NA19920 | **1/*3C* | **1/*3C* | **1/*3C* | **1/*37, *60* | **1/*80+*37* | **1/*80+*37* | **1/*2* | **1/*2* | **1/*2* |
| NA11832 | **1/*1* | **1/*1* | **1/*1* | **28, *60/*60* | **1/*80+*28* | **1/*80+*28* | **1/*2* | **1/*2* | **1/*2* |
| NA07029 | **1/*1* | **1/*1* | *N/A* | **1/*1* | **1/*1* | *N/A* | **2/*2* | **2/*2* | *N/A* |
| NA18868 | **1/*1* | **1/*1* | **1/*1* | **28, *60/*28, *60* | **80+*28/*80+*28* | **80/*80+*28 [X]* | **1/*2* | **1/*2* | **1/*2* |
| NA12813 | **1/*1* | **1/*1* | **1/*1* | **28, *60/*28, *60* | **80+*28/*80+*28* | **80+*28/*80+*28* | **1/*2* | **1/*2* | **1/*2* |
| HG00589 | **1/*3C* | **1/*3C* | **1/*3C* | **1/*7* | **1/*1* | **1/*1* | **1/*2* | **1/*2* | **1/*2* |
| NA20296 | **1/*3C* | **1/*3C* | **1/*3C* | **1/*60* | **1/*1* | **1/*1* | **1/*1* | **1/*1* | **1/*1* |
| NA12717 | **1/*1* | **1/*1* | **1/*1* | **1/*28, *60* | **1/*80+*28* | **1/*80+*28* | **1/*2* | **1/*2* | **1/*2* |
| NA07056 | **1/*1* | **1/*1* | **1/*1* | **1/*28, *60* | **1/*80+*28* | **1/*80+*28* | **2/*2* | **2/*2* | **2/*2* |
| NA18484 | **1/*1* | **1/*1* | **1/*1* | **28, *60/*60* | **1/*80+*28* | **1/*80+*28* | **1/*1* | **1/*1* | **1/*1* |
| NA19178 | **1/*1* | **1/*1* | **1/*1* | **28, *60/*36, *60* | **36/*80+*28* | **36/*80+*28* | **1/*1* | **1/*1* | **1/*1* |
| NA18564 | **1/*1* | **1/*1* | *N/A* | **1/*60* | **1/*1* | *N/A* | **1/*2* | **1/*2* | *N/A* |
| NA12145 | **1/*1* | **1/*1* | **1/*1* | **1/*1* | **1/*1* | **1/*1* | **1/*2* | **1/*2* | **1/*2* |
| NA18861 | **1/*1* | **1/*1* | **1/*1* | **60/*60* | **1/*1* | **1/*1* | **1/*2* | **1/*2* | **1/*2* |
| HG00436 | **1/*1* | **1/*1* | **1/*1* | **1/*27, *28, *60* | **1/*80+*28* | **1/*80+*28* | **1/*2* | **1/*2* | **1/*2* |
| NA18552 | **1/*1* | **1/*1* | **1/*1* | **1/*60* | **1/*1* | **1/*1* | **1/*1* | **1/*1* | **1/*1* |
| NA07000 | **1/*1* | **1/*1* | **1/*1* | **1/*1* | **1/*1* | **1/*1* | **1/*2* | **1/*2* | **1/*2* |
| NA12006 | **1/*1* | **1/*1* | **1/*1* | **1/*28, *60* | **1/*80+*28* | **1/*80+*28* | **1/*1* | **1/*1* | **1/*1* |
| NA19007 | **1/*1* | **1/*1* | **1/*1* | **1/*6* | **1/*6* | **1/*6* | **1/*3* | **1/*3* | **1/*3* |
| NA19239 | **1/*1* | **1/*1* | **1/*1* | **28, *60/*37, *60* | **80+*28/*80+*37* | **80+*28/*80+*37* | **1/*1* | **1/*1* | **1/*1* |
| NA12156 | **1/*1* | **1/*1* | **1/*1* | **1/*28, *60* | **1/*80+*28* | **1/*80+*28* | **1/*2* | **1/*2* | **1/*2* |
| NA19147 | **1/*1* | **1/*1* | **1/*1* | **28, *60/*28, *60* | **80+*28/*80+*28* | **80/*80+*28 [X]* | **1/*1* | **1/*1* | **1/*1* |
| NA19095 | **1/*1* | **1/*1* | **1/*1* | **28, *60/*36, *60* | **36/*80+*28* | **36/*80+*28* | **1/*2* | **1/*2* | **1/*2* |
| NA10854 | **1/*1* | **1/*1* | **1/*1* | **1/*1* | **1/*1* | **1/*1* | **1/*1* | **1/*1* | **1/*1* |
| NA18980 | **1/*1* | **1/*1* | **1/*1* | **6/*6* | **6/*6* | **6/*6* | **2/*3* | **2/*3* | **2/*3* |
| NA19207 | **1/*1* | **1/*1* | **1/*1* | **28, *60/*36, *60* | **36/*80+*28* | **36/*80+*28* | **1/*2* | **1/*2* | **1/*2* |
| NA18526 | **1/*1* | **1/*1* | **1/*1* | **1/*60* | **1/*1* | **1/*1* | **1/*2* | **1/*2* | **1/*2* |
| NA18959 | **1/*1* | **1/*1* | *N/A* | **1/*60* | **1/*1* | *N/A* | **1/*2* | **1/*2* | *N/A* |
| NA06991 | **1/*1* | **1/*1* | **1/*1* | **60/*60* | **1/*1* | **1/*1* | **1/*2* | **1/*2* | **1/*2* |
| NA19109 | **1/*1* | **1/*1* | **1/*1* | **1/*60* | **1/*1* | **1/*1* | **1/*1* | **1/*1* | **1/*1* |
| NA18952 | **1/*1* | **1/*1* | *N/A* | **1/*60* | **1/*1* | *N/A* | **1/*3* | **1/*3* | *N/A* |
| NA19789 | **1/*1* | **1/*1* | **1/*1* | **60/*60* | **1/*1* | **1/*1* | **1/*1* | **1/*1* | **1/*1* |
| HG01190 | **1/*1* | **1/*1* | **1/*1* | **37/*60* | **1/*80+*37* | **1/*80+*37* | **1/*2* | **1/*2* | **1/*2* |
| NA19122 | **1/*1* | **1/*1* | **1/*1* | **1/*28, *60* | **1/*80+*28* | **1/*80+*28* | **1/*1* | **1/*1* | **1/*1* |
| NA19819 | **1/*1* | **1/*1* | **1/*1* | **1/*28, *60* | **1/*80+*28* | **1/*80+*28* | **1/*1* | **1/*1* | **1/*1* |
| NA19213 | **1/*1* | **1/*1* | **1/*1* | **36, *60/*60* | **1/*36* | **1/*36* | **1/*2* | **1/*2* | **1/*2* |
| NA07055 | **1/*1* | **1/*1* | **1/*1* | **1/*1* | **1/*1* | **1/*1* | **1/*1* | **1/*1* | **1/*1* |
| NA19174 | **1/*1* | **1/*1* | *N/A* | **28, *60/*60* | **1/*80+*28* | *N/A* | **1/*1* | **1/*1* | *N/A* |
| NA20509 | **1/*1* | **1/*1* | **1/*1* | **1/*28, *60* | **1/*80+*28* | **1/*80+*28* | **1/*2* | **1/*2* | **1/*2* |
| NA18992 | **1/*1* | **1/*1* | **1/*1* | **1/*1* | **1/*1* | **1/*1* | **2/*3* | **2/*3* | **2/*3* |
| NA18565 | **1/*1* | **1/*1* | **1/*1* | **1/*60* | **1/*1* | **1/*1* | **2/*2* | **2/*2* | **2/*2* |
| NA18524 | **1/*1* | **1/*1* | **1/*1* | **1/*1* | **1/*1* | **1/*1* | **2/*2* | **2/*2* | **2/*2* |
| NA18942 | **1/*1* | **1/*1* | **1/*1* | **1/*28, *60* | **1/*80+*28* | **1/*80+*28* | **1/*1* | **1/*1* | **1/*1* |
| NA11839 | **1/*1* | **1/*1* | **1/*1* | **1/*1* | **1/*1* | **1/*1* | **1/*2* | **1/*2* | **1/*2* |
| NA10851 | **1/*1* | **1/*1* | **1/*1* | **1/*60* | **1/*1* | **1/*1* | **2/*2* | **2/*2* | **2/*2* |
| NA19176 | **1/*8* | **1/*8* | **1/*8* | **28, *60/*60* | **1/*80+*28* | **1/*80+*28* | **1/*1* | **1/*1* | **1/*1* |
| NA18509 | **1/*1* | **1/*1* | **1/*1* | **1/*28, *60* | **1/*80+*28* | **1/*80+*28* | **1/*2* | **1/*2* | **1/*2* |
| NA19226 | **1/*1* | **1/*1* | **1/*1* | **1/*60* | **1/*1* | **1/*1* | **1/*2* | **1/*2* | **1/*2* |
| NA07357 | **1/*1* | **1/*1* | **1/*1* | **28, *60/*60* | **1/*80+*28* | **1/*80+*28* | **1/*2* | **1/*2* | **1/*2* |
| NA07019 | **1/*1* | **1/*1* | **1/*1* | **1/*60* | **1/*1* | **1/*1* | **2/*2* | **2/*2* | **2/*2* |
| NA12873 | **1/*1* | **1/*1* | **1/*1* | **1/*1* | **1/*1* | **1/*1* | **1/*2* | **1/*2* | **1/*2* |
| NA19143 | **1/*1* | **1/*1* | **1/*1* | **28, *60/*60* | **1/*80+*28* | **1/*80+*28* | **1/*2* | **1/*2* | **1/*2* |
| NA10847 | **1/*1* | **1/*1* | *N/A* | **1/*28, *60* | **1/*80+*28* | *N/A* | **1/*2* | **1/*2* | *N/A* |
| NA18518 | **1/*1* | **1/*1* | **1/*1* | **60/*60* | **1/*1* | **1/*1* | **1/*2* | **1/*2* | **1/*2* |
| NA21781 | **1/*1* | **1/*1* | **1/*1* | **1/*28, *60* | **1/*80+*28* | **1/*80+*28* | **1/*2* | **1/*2* | **1/*2* |
| NA07348 | **1/*1* | **1/*1* | **1/*1* | **60/*60* | **1/*1* | **1/*1* | **1/*2* | **1/*2* | **1/*2* |
| NA19003 | **1/*1* | **1/*1* | **1/*1* | **1/*1* | **1/*1* | **1/*1* | **1/*2* | **1/*2* | **1/*2* |
| NA18544 | **1/*1* | **1/*1* | **1/*1* | **1/*28, *60* | **1/*80+*28* | **1/*80+*28* | **1/*1* | **1/*1* | **1/*1* |
| NA18540 | **1/*1* | **1/*1* | **1/*1* | **1/*28* | **1/*28* | **1/*28* | **1/*1/*2* | **1/*2* | **1/*2* |
| NA18966 | **1/*3C* | **1/*3C* | **1/*3C* | **1/*6* | **1/*6* | **1/*6* | **1/*2* | **1/*2* | **1/*2* |

|  | ***UGT2B15*** |  |  | ***UGT2B17*** |  |  | ***VKORC1*** |  |  |
| --- | --- | --- | --- | --- | --- | --- | --- | --- | --- |
| **Coriell ID** | **Previous^1^** | **WGS** | **ClinPharmSeq** | **Previous^1^** | **WGS** | **ClinPharmSeq** | **Previous^2^** | **WGS** | **ClinPharmSeq** |
| NA10831 | **5/*5* | **5/*5* | **5/*5* | **2/*2* | **2/*2* | **2/*2* | *AA* | *rs9923231/rs9923231* | *rs9923231/rs9923231* |
| NA18855 | **1/*2* | **1/*2* | **1/*2* | **1/*2* | **1/*2* | **1/*2* | *GG* | *Reference/Reference* | *Reference/Reference* |
| NA18617 | **1/*1* | **1/*1* | **1/*1* | **2/*2* | **2/*2* | **2/*2* | *AA* | *rs9923231/rs9923231* | *rs9923231/rs9923231* |
| NA19908 | **1/*4* | **1/*4* | **1/*4* | **2/*2* | **2/*2* | **2/*2* | *GG* | *Reference/Reference* | *Reference/Reference* |
| NA18973 | **1/*2* | **1/*2* | *N/A* | **2/*2* | **2/*2* | *N/A* | *AA* | *rs9923231/rs9923231* | *N/A* |
| NA12003 | **2/*5* | **2/*5* | **2/*5* | **1/*1* | **1/*1* | **1/*1* | *AA* | *rs9923231/rs9923231* | *rs9923231/rs9923231* |
| NA18519 | **1/*1* | **1/*1* | **1/*1* | **1/*1* | **1/*1* | **1/*1* | *GG* | *Reference/Reference* | *Reference/Reference* |
| HG00276 | **1/*5* | **1/*5* | **1/*5* | **2/*2* | **2/*2* | **2/*2* | *GA* | *Reference/rs9923231* | *Reference/rs9923231* |
| NA11993 | **4/*S1* | **4/*S1* | **4/*S1* | **1/*1* | **1/*1* | **1/*1* | *GA* | *Reference/rs9923231* | *Reference/rs9923231* |
| NA19917 | **1/*1* | **1/*1* | **1/*1* | **1/*2* | **1/*2* | **1/*2* | *GG* | *Reference/Reference* | *Reference/Reference* |
| NA19920 | **1/*2* | **1/*2* | **1/*2* | **1/*1* | **1/*1* | **1/*1* | *GG* | *Reference/Reference* | *Reference/Reference* |
| NA11832 | **2/*4* | **2/*4* | **1/*5 [P]* | **1/*2* | **1/*2* | **1/*2* | *GG* | *Reference/Reference* | *Reference/Reference* |
| NA07029 | **2/*4* | **2/*4* | *N/A* | **1/*2* | **1/*2* | *N/A* | *GA* | *Reference/rs9923231* | *N/A* |
| NA18868 | **1/*1* | **1/*1* | **1/*1* | **1/*2* | **1/*2* | **1/*2* | *GG* | *Reference/Reference* | *Reference/Reference* |
| NA12813 | **2/*5* | **2/*5* | **2/*5* | **2/*2* | **2/*2* | **2/*2* | *GG* | *Reference/Reference* | *Reference/Reference* |
| HG00589 | **1/*2* | **1/*2* | **1/*2* | **2/*2* | **2/*2* | **2/*2* | *AA* | *rs9923231/rs9923231* | *rs9923231/rs9923231* |
| NA20296 | **2/*4* | **2/*4* | **2/*4* | **2/*2* | **2/*2* | **2/*2* | *GA* | *Reference/rs9923231* | *Reference/rs9923231* |
| NA12717 | **4/*4* | **4/*4* | **4/*4* | **1/*2* | **1/*2* | **1/*2* | *GG* | *Reference/Reference* | *Reference/Reference* |
| NA07056 | **4/*5* | **4/*5* | **4/*5* | **1/*2* | **1/*2* | **1/*2* | *GA* | *Reference/rs9923231* | *Reference/rs9923231* |
| NA18484 | **1/*2* | **1/*2* | **1/*2* | **1/*1* | **1/*1* | **1/*1* | *GG* | *Reference/Reference* | *Reference/Reference* |
| NA19178 | **2/*2* | **2/*2* | **2/*2* | **1/*1* | **1/*1* | **1/*1* | *GG* | *Reference/Reference* | *Reference/Reference* |
| NA18564 | **2/*2* | **2/*2* | *N/A* | **1/*2* | **1/*2* | *N/A* | *AA* | *rs9923231/rs9923231* | *N/A* |
| NA12145 | **1/*4* | **1/*4* | **1/*4* | **1/*2* | **1/*2* | **1/*2* | *GA* | *Reference/rs9923231* | *Reference/rs9923231* |
| NA18861 | **1/*2* | **1/*2* | **1/*2* | **1/*1* | **1/*1* | **1/*1* | *GG* | *Reference/Reference* | *Reference/Reference* |
| HG00436 | **2/*5* | **2/*5* | **2/*5* | **1/*2* | **1/*2* | **1/*2* | *GA* | *Reference/rs9923231* | *Reference/rs9923231* |
| NA18552 | **1/*2* | **1/*2* | **1/*2* | **2/*2* | **2/*2* | **2/*2* | *AA* | *rs9923231/rs9923231* | *rs9923231/rs9923231* |
| NA07000 | **2/*4* | **2/*4* | **2/*4* | **1/*1* | **1/*1* | **1/*1* | *GA* | *Reference/rs9923231* | *Reference/rs9923231* |
| NA12006 | **5/*5* | **5/*5* | **5/*5* | **1/*1* | **1/*1* | **1/*1* | *GA* | *Reference/rs9923231* | *Reference/rs9923231* |
| NA19007 | **1/*2* | **1/*2* | **1/*2* | **2/*2* | **2/*2* | **2/*2* | *AA* | *rs9923231/rs9923231* | *rs9923231/rs9923231* |
| NA19239 | **1/*1* | **1/*1* | **1/*1* | **1/*2* | **1/*2* | **1/*2* | *GG* | *Reference/Reference* | *Reference/Reference* |
| NA12156 | **5/*5* | **5/*5* | **5/*5* | **1/*2* | **1/*2* | **1/*2* | *GA* | *Reference/rs9923231* | *Reference/rs9923231* |
| NA19147 | **2/*2* | **2/*2* | **2/*2* | **1/*2* | **1/*2* | **1/*2* | *GA* | *Reference/rs9923231* | *Reference/rs9923231* |
| NA19095 | **1/*1* | **1/*1* | **1/*1* | **1/*2* | **1/*2* | **1/*2* | *GG* | *Reference/Reference* | *Reference/Reference* |
| NA10854 | **2/*5* | **2/*5* | **2/*5* | **1/*1* | **1/*1* | **1/*1* | *GA* | *Reference/rs9923231* | *Reference/rs9923231* |
| NA18980 | **1/*2* | **1/*2* | **1/*2* | **2/*2* | **2/*2* | **2/*2* | *GA* | *Reference/rs9923231* | *Reference/rs9923231* |
| NA19207 | **1/*2* | **1/*2* | **1/*2* | **1/*1* | **1/*1* | **1/*1* | *GG* | *Reference/Reference* | *Reference/Reference* |
| NA18526 | **1/*1* | **1/*1* | **1/*1* | **2/*2* | **2/*2* | **2/*2* | *AA* | *rs9923231/rs9923231* | *rs9923231/rs9923231* |
| NA18959 | **1/*2* | **1/*2* | *N/A* | **2/*2* | **2/*2* | *N/A* | *AA* | *rs9923231/rs9923231* | *N/A* |
| NA06991 | **4/*5* | **4/*5* | **4/*5* | **1/*1* | **1/*1* | **1/*1* | *AA* | *rs9923231/rs9923231* | *rs9923231/rs9923231* |
| NA19109 | **1/*2* | **1/*2* | **1/*2* | **1/*2* | **1/*2* | **1/*2* | *GG* | *Reference/Reference* | *Reference/Reference* |
| NA18952 | **1/*5* | **1/*5* | *N/A* | **2/*2* | **2/*2* | *N/A* | *GA* | *Reference/rs9923231* | *N/A* |
| NA19789 | **1/*1* | **1/*1* | **1/*1* | **1/*1* | **1/*1* | **1/*1* | *GG* | *Reference/Reference* | *Reference/Reference* |
| HG01190 | **4/*5* | **4/*5* | **4/*5* | **1/*1* | **1/*1* | **1/*1* | *GG* | *Reference/Reference* | *Reference/Reference* |
| NA19122 | **2/*2* | **2/*2* | **2/*2* | **1/*1* | **1/*1* | **1/*1* | *GG* | *Reference/Reference* | *Reference/Reference* |
| NA19819 | **1/*4* | **1/*4* | **1/*4* | **1/*1* | **1/*1* | **1/*1* | *GA* | *Reference/rs9923231* | *Reference/rs9923231* |
| NA19213 | **1/*2* | **1/*2* | **1/*2* | **1/*2* | **1/*2* | **1/*2* | *GG* | *Reference/Reference* | *Reference/Reference* |
| NA07055 | **1/*2* | **1/*2* | **1/*2* | **1/*2* | **1/*2* | **1/*2* | *AA* | *rs9923231/rs9923231* | *rs9923231/rs9923231* |
| NA19174 | **1/*2* | **1/*2* | *N/A* | **1/*1* | **1/*1* | *N/A* | *GG* | *Reference/Reference* | *N/A* |
| NA20509 | **2/*5* | **2/*5* | **2/*5* | **1/*2* | **1/*2* | **1/*2* | *GA* | *Reference/rs9923231* | *Reference/rs9923231* |
| NA18992 | **2/*2* | **2/*2* | **2/*2* | **2/*2* | **2/*2* | **2/*2* | *AA* | *rs9923231/rs9923231* | *rs9923231/rs9923231* |
| NA18565 | **2/*2* | **2/*2* | **2/*2* | **2/*2* | **2/*2* | **2/*2* | *AA* | *rs9923231/rs9923231* | *rs9923231/rs9923231* |
| NA18524 | **2/*4* | **2/*4* | **1/*5 [P]* | **1/*2* | **1/*2* | **1/*2* | *AA* | *rs9923231/rs9923231* | *rs9923231/rs9923231* |
| NA18942 | **1/*4* | **1/*4* | **1/*4* | **2/*2* | **2/*2* | **2/*2* | *AA* | *rs9923231/rs9923231* | *rs9923231/rs9923231* |
| NA11839 | **2/*4* | **2/*4* | **1/*5 [P]* | **1/*2* | **1/*2* | **1/*2* | *GA* | *Reference/rs9923231* | *Reference/rs9923231* |
| NA10851 | **5/*5* | **5/*5* | **5/*5* | **2/*2* | **2/*2* | **2/*2* | *GA* | *Reference/rs9923231* | *Reference/rs9923231* |
| NA19176 | **2/*4* | **2/*4* | **2/*4* | **1/*1* | **1/*1* | **1/*1* | *GG* | *Reference/Reference* | *Reference/Reference* |
| NA18509 | **1/*2* | **1/*2* | **1/*2* | **1/*1* | **1/*1* | **1/*1* | *GG* | *Reference/Reference* | *Reference/Reference* |
| NA19226 | **1/*1* | **1/*1* | **1/*1* | **1/*1* | **1/*1* | **1/*1* | *GG* | *Reference/Reference* | *Reference/Reference* |
| NA07357 | **2/*4* | **2/*4* | **2/*4* | **1/*1* | **1/*1* | **1/*1* | *GG* | *Reference/Reference* | *Reference/Reference* |
| NA07019 | **4/*5* | **4/*5* | **4/*5* | **1/*2* | **1/*2* | **1/*2* | *GG* | *Reference/Reference* | *Reference/Reference* |
| NA12873 | **2/*4* | **1/*5 [P]* | **1/*5 [P]* | **1/*2* | **1/*2* | **1/*2* | *GG* | *Reference/Reference* | *Reference/Reference* |
| NA19143 | **1/*1* | **1/*1* | **1/*1* | **1/*2* | **1/*2* | **1/*2* | *GG* | *Reference/Reference* | *Reference/Reference* |
| NA10847 | **2/*2* | **2/*2* | *N/A* | **1/*1* | **1/*1* | *N/A* | *GG* | *Reference/Reference* | *N/A* |
| NA18518 | **1/*2* | **1/*2* | **1/*2* | **1/*1* | **1/*1* | **1/*1* | *GG* | *Reference/Reference* | *Reference/Reference* |
| NA21781 | **1/*4* | **1/*4* | **1/*4* | **1/*1* | **1/*1* | **1/*1* | *GA* | *Reference/rs9923231* | *Reference/rs9923231* |
| NA07348 | **2/*5* | **2/*5* | **2/*5* | **1/*2* | **1/*2* | **1/*2* | *GG* | *Reference/Reference* | *Reference/Reference* |
| NA19003 | **2/*2* | **2/*2* | **2/*2* | **1/*2* | **1/*2* | **1/*2* | *AA* | *rs9923231/rs9923231* | *rs9923231/rs9923231* |
| NA18544 | **1/*2* | **1/*2* | **1/*2* | **2/*2* | **2/*2* | **2/*2* | *AA* | *rs9923231/rs9923231* | *rs9923231/rs9923231* |
| NA18540 | **2/*2/*4* | **2/*4* | **2/*4* | **2/*2/*2* | **2/*2* | **2/*2* | *AA* | *rs9923231/rs9923231* | *rs9923231/rs9923231* |
| NA18966 | **1/*2* | **1/*2* | **1/*2* | **2/*2* | **2/*2* | **2/*2* | *AA* | *rs9923231/rs9923231* | *rs9923231/rs9923231* |

^1^Diplotype calls from Lee et al., 2019.

^2^Diplotype calls from Pratt et al., 2016.

^3^Diplotype calls from Gaedigk et al., 2019.

[N] indicates diplotype discrepancies that are caused because one of the *CYP1A2*1L* variants (15-75038220-G-A) is not targeted by the current version of ClinPharmSeq. [P] indicates diplotype discrepancies that are attributed to alternative haplotype phasing of variants. [X] indicates diplotype discrepancies that are due to difference in variant calling.
