# Supplementary material for: ClinPharmSeq: A targeted sequencing panel for clinical pharmacogenetics implementation
Source: PLoS One. 2022 Jul 28;17(7):e0272129. doi: 10.1371/journal.pone.0272129 (PMC9333201; doi:10.1371/journal.pone.0272129)
Supplement: S3 Table — (DOCX) [file pone.0272129.s007.docx]

| No. | Coriell ID | Gene | Previous | WGS | ClinPharmSeq | Explanation |
| --- | --- | --- | --- | --- | --- | --- |
| 1 | NA10831 | *CYP1A2* | **1A/*1F* | **1A/*1F* | **1A/*1A [X]* | The **1F* variant (15-75041917-C-A) was not detected with ClinPharmSeq. |
| 2 | NA18855 | *CYP1A2* | **1A/*1L* | **1A/*1L* | **1A/*1F [N]* | One of the **1L* variants (15-75038220-G-A) is not targeted by ClinPharmSeq. |
| 3 | NA19908 | *CYP1A2* | **1L/*1L* | **1L/*1L* | **1F/*1F [N]* | One of the **1L* variants (15-75038220-G-A) is not targeted by ClinPharmSeq. |
| 4 | NA18519 | *CYP1A2* | **1A/*1L* | **1A/*1L* | **1A/*1F [N]* | One of the **1L* variants (15-75038220-G-A) is not targeted by ClinPharmSeq. |
| 5 | NA18868 | *CYP1A2* | **1A/*1L* | **1A/*1L* | **1A/*1F [N]* | One of the **1L* variants (15-75038220-G-A) is not targeted by ClinPharmSeq. |
| 6 | NA12813 | *CYP1A2* | **1F/*1L* | **1F/*1L* | **1F/*1F [N]* | One of the **1L* variants (15-75038220-G-A) is not targeted by ClinPharmSeq. |
| 7 | HG00589 | *CYP1A2* | **1A/*1L* | **1A/*1L* | **1A/*1F [N]* | One of the **1L* variants (15-75038220-G-A) is not targeted by ClinPharmSeq. |
| 8 | NA20296 | *CYP1A2* | **1L/*1L* | **1L/*1L* | **1F/*1F [N]* | One of the **1L* variants (15-75038220-G-A) is not targeted by ClinPharmSeq. |
| 9 | NA19178 | *CYP1A2* | **1L/*1L* | **1L/*1L* | **1F/*1F [N]* | One of the **1L* variants (15-75038220-G-A) is not targeted by ClinPharmSeq. |
| 10 | NA18861 | *CYP1A2* | **1A/*1L* | **1A/*1L* | **1A/*1F [N]* | One of the **1L* variants (15-75038220-G-A) is not targeted by ClinPharmSeq. |
| 11 | NA19007 | *CYP1A2* | **1A/*1L* | **1A/*1L* | **1A/*1F [N]* | One of the **1L* variants (15-75038220-G-A) is not targeted by ClinPharmSeq. |
| 12 | NA19239 | *CYP1A2* | **1A/*1L* | **1A/*1L* | **1A/*1F [N]* | One of the **1L* variants (15-75038220-G-A) is not targeted by ClinPharmSeq. |
| 13 | NA19095 | *CYP1A2* | **1A/*1L* | **1A/*1L* | **1A/*1F [N]* | One of the **1L* variants (15-75038220-G-A) is not targeted by ClinPharmSeq. |
| 14 | NA18980 | *CYP1A2* | **1A/*1L* | **1A/*1L* | **1A/*1F [N]* | One of the **1L* variants (15-75038220-G-A) is not targeted by ClinPharmSeq. |
| 15 | NA18526 | *CYP1A2* | **1A/*1L* | **1A/*1L* | **1A/*1F [N]* | One of the **1L* variants (15-75038220-G-A) is not targeted by ClinPharmSeq. |
| 16 | NA19789 | *CYP1A2* | **1A/*1L* | **1A/*1L* | **1A/*1F [N]* | One of the **1L* variants (15-75038220-G-A) is not targeted by ClinPharmSeq. |
| 17 | NA19122 | *CYP1A2* | **1A/*1L* | **1A/*1L* | **1A/*1F [N]* | One of the **1L* variants (15-75038220-G-A) is not targeted by ClinPharmSeq. |
| 18 | NA19819 | *CYP1A2* | **1F/*1L* | **1F/*1L* | **1F/*1F [N]* | One of the **1L* variants (15-75038220-G-A) is not targeted by ClinPharmSeq. |
| 19 | NA18544 | *CYP1A2* | **1F/*1L* | **1F/*1L* | **1F/*1F [N]* | One of the **1L* variants (15-75038220-G-A) is not targeted by ClinPharmSeq. |
| 20 | NA18540 | *CYP1A2* | **1L/*1L* | **1L/*1L* | **1F/*1F [N]* | One of the **1L* variants (15-75038220-G-A) is not targeted by ClinPharmSeq. |
| 21 | HG00436 | *CYP2A6* | **4/*1+*S6* | *Indeterminate [X]* | *Indeterminate [X]* | PyPGx detected gene hybrid but did not produce the final genotype. |
| 22 | NA07055 | *CYP2A6* | **1/*22* | **1/*22* | **1/*1 [X]* | Both of the **22* variants (19-41354534-G-T, 19-41354538-G-C) were not detected with ClinPharmSeq. |
| 23 | NA12003 | *CYP2C8* | **1/*3* | **1/*3* | **1/*1 [P]* | The **3* variants (3:10-96798749-T-C, 10-96827030-C-T) were phased in trans with ClinPharmSeq. |
| 24 | NA18565 | *CYP2D6* | **10/*36x2* | **10/*36+*10 [X]* | **10/*36+*10 [X]* | PyPGx called **36+*10* instead of **36x2*. |
| 25 | NA18540 | *CYP2D6* | *(*36+)10/*41* | **36x2+*10/*41 [X]* | **36x2+*10/*41 [X]* | PyPGx called **36x2+*10* instead of **36+*10*. |
| 26 | NA19908 | *CYP2E1* | **7x2/*7x2* | **7/*7x3 [X]* | **7/*7x3 [X]* | PyPGx called **7x3* instead of **7x2*. |
| 27 | NA18509 | *CYP4F2* | **1/*3, *2* | **1/*2* | **1/*3 [X]* | The **2* variant (19-16008388-A-C) was not detected with ClinPharmSeq. |
| 28 | NA19147 | *SLC15A2* | **1/*2* | **1/*2* | **1/*1 [P]* | The **2* variants (2:3-121648168-G-A, 3-121647286-C-T, 3-121643804-C-T) were not phased in cis with ClinPharmSeq. |
| 29 | NA18992 | *SLC15A2* | **1/*2* | **1/*2* | **1/*1 [P]* | The **2* variants (2:3-121648168-G-A, 3-121647286-C-T, 3-121643804-C-T) were not phased in cis with ClinPharmSeq. |
| 30 | NA12873 | *SLC15A2* | **1/*2* | **1/*2* | **1/*1 [P]* | The **2* variants (2:3-121648168-G-A, 3-121647286-C-T, 3-121643804-C-T) were not phased in cis with ClinPharmSeq. |
| 31 | NA18519 | *SLC22A2* | **1/*3* | **1/*2* | **2/*3 [P]* | The **1* variants (6-160645832-C-T, 6-160670282-A-C) were phased in trans with ClinPharmSeq. |
| 32 | NA12145 | *SLC22A2* | **2/*3* | **2/*3* | **1/*2 [P]* | The **1* variants (6-160645832-C-T, 6-160670282-A-C) were phased in cis with ClinPharmSeq. |
| 33 | NA18980 | *SLCO2B1* | **S1/*S464F* | **S1/*S464F* | **1/*S464F [P]* | The variants of **S1* and **464F* (11-74873754-GCACAGAAAA-G, 11-74907582-C-T) were phased in cis with ClinPharmSeq. |
| 34 | NA18540 | *SLCO2B1* | **S464F/*S464F* | **S464F/*S464F* | **1/*S464F [X]* | The **S464F* variant (11-74907582-C-T) was called as heterozygous with ClinPharmSeq. |
| 35 | NA18855 | *UGT1A1* | **28, *60/*28, *60* | **80+*28/*80+*28* | **80/*80+*28 [X]* | The **28* variant (2-234668879-C-CAT) was called as heterozygous with ClinPharmSeq. |
| 36 | NA18868 | *UGT1A1* | **28, *60/*28, *60* | **80+*28/*80+*28* | **80/*80+*28 [X]* | The **28* variant (2-234668879-C-CAT) was called as heterozygous with ClinPharmSeq. |
| 37 | NA19147 | *UGT1A1* | **28, *60/*28, *60* | **80+*28/*80+*28* | **80/*80+*28 [X]* | The **28* variant (2-234668879-C-CAT) was called as heterozygous with ClinPharmSeq. |
| 38 | NA11832 | *UGT2B15* | **2/*4* | **2/*4* | **1/*5 [P]* | The **4* variants (4-69536084-A-C, 4-69512847-T-G) were phased in trans with ClinPharmSeq. |
| 39 | NA18524 | *UGT2B15* | **2/*4* | **2/*4* | **1/*5 [P]* | The **4* variants (4-69536084-A-C, 4-69512847-T-G) were phased in trans with ClinPharmSeq. |
| 40 | NA11839 | *UGT2B15* | **2/*4* | **2/*4* | **1/*5 [P]* | The **4* variants (4-69536084-A-C, 4-69512847-T-G) were phased in trans with ClinPharmSeq. |
| 41 | NA12873 | *UGT2B15* | **2/*4* | **1/*5 [P]* | **1/*5 [P]* | The **4* variants (4-69536084-A-C, 4-69512847-T-G) were phased in trans with both WGS and ClinPharmSeq. |
