# Supplementary material for: ClinPharmSeq: A targeted sequencing panel for clinical pharmacogenetics implementation
Source: PLoS One. 2022 Jul 28;17(7):e0272129. doi: 10.1371/journal.pone.0272129 (PMC9333201; doi:10.1371/journal.pone.0272129)
Supplement: S4 Table — (DOCX) [file pone.0272129.s008.docx]

| No. | Gene | Star Allele | Explanation |
| --- | --- | --- | --- |
| 1 | *CYP2A6* | **35* | PyPGx does not call this allele because its variant (22-42523514-C-T) has a high false positive rate. |
| 2 | *CYP2C8* | **15* | The allele was not tested previously. |
| 3 | *CYP2C8* | **16* | The allele was not tested previously. |
| 4 | *CYP2C8* | **17* | The allele was not tested previously. |
| 5 | *CYP2C9* | **S1* | This allele is not defined in PyPGx. |
| 6 | *CYP2C9* | **61* | The allele was not tested previously. |
| 7 | *CYP2C19* | **27* | PyPGx does not call this allele because it has been reassigned to **1* by PharmVar. |
| 8 | *CYP2C19* | **39* | The allele was not tested previously. |
| 9 | *CYP2D6* | **106* | The allele was not tested previously. |
| 10 | *DPYD* | *c.1349C>T* | The allele was not tested previously. |
| 11 | *DPYD* | *c.1682G>T* | The allele was not tested previously. |
| 12 | *DPYD* | *c.2846A>T* | The allele was not tested previously. |
| 13 | *GSTT1* | **B* | PyPGx does not call this allele because its variant (22-24379402-T-G) has been removed from dbSNP. |
| 14 | *NAT2* | **24* | The allele was not tested previously. |
| 15 | *SLCO1B1* | **20* | PyPGx called **20* instead of **35*. **20* has all the variants of **35*. |
| 16 | *SLCO1B1* | **32* | PyPGx called **32* instead of **14*. **32* has all the variants of **14*. |
| 17 | *UGT1A1* | **80+*27* | The allele was not tested previously. |
| 18 | *UGT1A1* | **80+*37* | The allele was not tested previously. |
| 19 | *UGT1A1* | **60* | PyPGx does not call this allele because it has been removed from the CPIC allele definition table. |

Abbreviations: dbSNP, Single Nucleotide Polymorphism Database; CPIC, Clinical Pharmacogenetics Implementation Consortium; PharmGKB, Pharmacogenomics Knowledge Base.
