# Supplementary material for: ClinPharmSeq: A targeted sequencing panel for clinical pharmacogenetics implementation
Source: PLoS One. 2022 Jul 28;17(7):e0272129. doi: 10.1371/journal.pone.0272129 (PMC9333201; doi:10.1371/journal.pone.0272129)
Supplement: S5 Table — (DOCX) [file pone.0272129.s009.docx]

|  | ***CYP2B6*** |  |  | ***CYP2C9*** |  |  | ***CYP2C19*** |  |  |
| --- | --- | --- | --- | --- | --- | --- | --- | --- | --- |
| **Coriell ID** | **Previous** | **WGS** | **ClinPharmSeq** | **Previous** | **WGS** | **ClinPharmSeq** | **Previous** | **WGS** | **ClinPharmSeq** |
| NA10831 | NM | NM | NM | IM | IM | IM | RM | RM | RM |
| NA18855 | PM | PM | PM | NM | NM | NM | IM | IM | IM |
| NA18617 | RM | RM | RM | NM | NM | NM | IM | IM | IM |
| NA19908 | IM | IM | IM | IM | IM | IM | RM | RM | RM |
| NA18973 | IM | IM | N/A | NM | NM | N/A | NM | NM | N/A |
| NA12003 | PM | PM | PM | IM | IM | IM | NM | NM | NM |
| NA18519 | IM | IM | IM | IM | IM | IM | RM | RM | RM |
| HG00276 | RM | RM | RM | IM | IM | IM | NM | NM | NM |
| NA11993 | NM | NM | NM | NM | NM | NM | NM | NM | NM |
| NA19917 | PM | PM | PM | NM | NM | NM | IM | IM | IM |
| NA19920 | PM | PM | PM | NM | NM | NM | NM | NM | NM |
| NA11832 | NM | NM | NM | IM | IM | IM | IM | IM | IM |
| NA07029 | IM | IM | N/A | IM | IM | N/A | IM | IM | N/A |
| NA18868 | IM | IM | IM | NM | NM | NM | IM | IM | IM |
| NA12813 | NM | NM | NM | IM | IM | IM | RM | RM | RM |
| HG00589 | NM | NM | NM | NM | NM | NM | NM | NM | NM |
| NA20296 | NM | NM | NM | NM | NM | NM | NM | NM | NM |
| NA12717 | IM | IM | IM | NM | NM | NM | PM | PM | PM |
| NA07056 | IM | IM | IM | NM | NM | NM | NM | NM | NM |
| NA18484 | IM | IM | IM | NM | NM | NM | IM | IM | IM |
| NA19178 | PM | PM | PM | IM | IM | IM | IM | IM | IM |
| NA18564 | NM | NM | N/A | NM | NM | N/A | PM | PM | N/A |
| NA12145 | NM | NM | NM | NM | NM | NM | IM | IM | IM |
| NA18861 | IM | IM | IM | NM | NM | NM | NM | NM | NM |
| HG00436 | IM | IM | IM | NM | NM | NM | NM | NM | NM |
| NA18552 | IM | IM | IM | NM | NM | NM | IM | IM | IM |
| NA07000 | NM | NM | NM | NM | NM | NM | RM | RM | RM |
| NA12006 | NM | NM | NM | NM | NM | NM | NM | NM | NM |
| NA19007 | I | I | I | NM | NM | NM | NM | NM | NM |
| NA19239 | IM | IM | IM | NM | NM | NM | RM | RM | RM |
| NA12156 | NM | NM | NM | IM | IM | IM | NM | NM | NM |
| NA19147 | IM | IM | IM | NM | NM | NM | RM | RM | RM |
| NA19095 | PM | PM | PM | NM | NM | NM | NM | NM | NM |
| NA10854 | NM | NM | NM | IM | IM | IM | NM | NM | NM |
| NA18980 | PM | PM | PM | NM | NM | NM | NM | NM | NM |
| NA19207 | IM | IM | IM | NM | NM | NM | IM | IM | IM |
| NA18526 | NM | NM | NM | NM | NM | NM | NM | NM | NM |
| NA18959 | NM | NM | N/A | IM | IM | N/A | NM | NM | N/A |
| NA06991 | IM | IM | IM | NM | NM | NM | NM | NM | NM |
| NA19109 | IM | IM | IM | NM | NM | NM | UM | UM | UM |
| NA18952 | NM | NM | N/A | NM | NM | N/A | NM | NM | N/A |
| NA19789 | NM | NM | NM | IM | IM | IM | NM | NM | NM |
| HG01190 | NM | NM | NM | IM | IM | IM | IM | IM | IM |
| NA19122 | PM | PM | PM | IM | IM | IM | PM | PM | PM |
| NA19819 | NM | NM | NM | NM | NM | NM | RM | RM | RM |
| NA19213 | PM | PM | PM | IM | IM | IM | NM | I [X] | I [X] |
| NA07055 | IM | IM | IM | NM | NM | NM | RM | RM | RM |
| NA19174 | PM | PM | N/A | NM | NM | N/A | IM | IM | N/A |
| NA20509 | IM | IM | IM | NM | NM | NM | PM | PM | PM |
| NA18992 | IM | IM | IM | NM | NM | NM | NM | NM | NM |
| NA18565 | NM | NM | NM | NM | NM | NM | NM | NM | NM |
| NA18524 | NM | NM | NM | IM | IM | IM | IM | IM | IM |
| NA18942 | NM | NM | NM | NM | NM | NM | NM | NM | NM |
| NA11839 | I | I | I | PM | PM | PM | NM | NM | NM |
| NA10851 | NM | NM | NM | NM | NM | NM | RM | RM | RM |
| NA19176 | PM | PM | PM | NM | NM | NM | IM | IM | IM |
| NA18509 | IM | IM | IM | NM | NM | NM | PM | PM | PM |
| NA19226 | PM | PM | PM | IM | IM | IM | IM | IM | IM |
| NA07357 | NM | NM | NM | NM | NM | NM | IM | IM | IM |
| NA07019 | RM | RM | RM | NM | NM | NM | RM | RM | RM |
| NA12873 | IM | IM | IM | NM | NM | NM | RM | RM | RM |
| NA19143 | PM | PM | PM | IM | IM | IM | NM | I [X] | I [X] |
| NA10847 | IM | IM | N/A | NM | NM | N/A | NM | NM | N/A |
| NA18518 | IM | IM | IM | NM | NM | NM | IM | IM | IM |
| NA21781 | RM | RM | RM | NM | NM | NM | IM | IM | IM |
| NA07348 | NM | NM | NM | NM | NM | NM | IM | IM | IM |
| NA19003 | IM | IM | IM | NM | NM | NM | IM | IM | IM |
| NA18544 | NM | NM | NM | NM | NM | NM | IM | IM | IM |
| NA18540 | IM | IM | IM | NM | NM | NM | IM | IM | IM |
| NA18966 | IM | IM | IM | NM | NM | NM | NM | NM | NM |

|  | ***CYP2D6*** |  |  | ***CYP3A5*** |  |  | ***DPYD*** |  |  |
| --- | --- | --- | --- | --- | --- | --- | --- | --- | --- |
| **Coriell ID** | **Previous** | **WGS** | **ClinPharmSeq** | **Previous** | **WGS** | **ClinPharmSeq** | **Previous** | **WGS** | **ClinPharmSeq** |
| NA10831 | PM | PM | PM | PM | PM | PM | NM | NM | NM |
| NA18855 | IM | IM | IM | PM | PM | PM | NM | NM | NM |
| NA18617 | IM | IM | IM | PM | PM | PM | NM | NM | NM |
| NA19908 | NM | NM | NM | IM | IM | IM | NM | NM | NM |
| NA18973 | IM | IM | N/A | IM | IM | N/A | NM | NM | N/A |
| NA12003 | IM | IM | IM | IM | IM | IM | NM | NM | NM |
| NA18519 | NM | I [X] | I [X] | IM | IM | IM | NM | NM | NM |
| HG00276 | PM | PM | PM | PM | PM | PM | NM | NM | NM |
| NA11993 | NM | NM | NM | PM | PM | PM | NM | NM | NM |
| NA19917 | IM | IM | IM | IM | IM | IM | NM | NM | NM |
| NA19920 | IM | IM | IM | PM | PM | PM | NM | NM | NM |
| NA11832 | IM | IM | IM | PM | PM | PM | NM | NM | NM |
| NA07029 | NM | NM | N/A | IM | IM | N/A | NM | NM | N/A |
| NA18868 | IM | IM | IM | IM | IM | IM | NM | NM | NM |
| NA12813 | IM | IM | IM | PM | PM | PM | NM | NM | NM |
| HG00589 | IM | IM | IM | PM | PM | PM | NM | NM | NM |
| NA20296 | NM | NM | NM | IM | IM | IM | NM | NM | NM |
| NA12717 | NM | NM | NM | IM | IM | IM | NM | NM | NM |
| NA07056 | IM | IM | IM | PM | PM | PM | NM | NM | NM |
| NA18484 | NM | NM | NM | IM | IM | IM | NM | NM | NM |
| NA19178 | NM | NM | NM | NM | NM | NM | NM | NM | NM |
| NA18564 | NM | NM | N/A | NM | NM | N/A | NM | NM | N/A |
| NA12145 | IM | IM | IM | PM | PM | PM | NM | NM | NM |
| NA18861 | IM | IM | IM | NM | NM | NM | NM | NM | NM |
| HG00436 | I | I | I | PM | PM | PM | NM | NM | NM |
| NA18552 | NM | NM | NM | PM | PM | PM | NM | NM | NM |
| NA07000 | NM | NM | NM | IM | IM | IM | NM | NM | NM |
| NA12006 | IM | IM | IM | PM | PM | PM | NM | NM | NM |
| NA19007 | NM | NM | NM | PM | PM | PM | NM | NM | NM |
| NA19239 | IM | IM | IM | NM | NM | NM | NM | NM | NM |
| NA12156 | IM | IM | IM | PM | PM | PM | NM | NM | NM |
| NA19147 | IM | IM | IM | IM | IM | IM | NM | NM | NM |
| NA19095 | NM | NM | NM | IM | IM | IM | NM | NM | NM |
| NA10854 | IM | IM | IM | IM | IM | IM | NM | NM | NM |
| NA18980 | NM | NM | NM | IM | IM | IM | NM | NM | NM |
| NA19207 | NM | NM | NM | PM | PM | PM | NM | IM [X] | IM [X] |
| NA18526 | NM | NM | NM | NM | NM | NM | NM | NM | NM |
| NA18959 | NM | NM | N/A | IM | IM | N/A | NM | NM | N/A |
| NA06991 | IM | IM | IM | PM | PM | PM | NM | IM [X] | IM [X] |
| NA19109 | UM | UM | UM | IM | IM | IM | NM | NM | NM |
| NA18952 | NM | NM | N/A | PM | PM | N/A | NM | NM | N/A |
| NA19789 | NM | NM | NM | PM | PM | PM | NM | NM | NM |
| HG01190 | PM | PM | PM | NM | NM | NM | NM | NM | NM |
| NA19122 | NM | NM | NM | NM | NM | NM | NM | NM | NM |
| NA19819 | IM | IM | IM | PM | PM | PM | NM | NM | NM |
| NA19213 | NM | NM | NM | IM | IM | IM | NM | NM | NM |
| NA07055 | PM | PM | PM | PM | PM | PM | NM | IM [X] | IM [X] |
| NA19174 | PM | PM | N/A | IM | IM | N/A | NM | NM | N/A |
| NA20509 | IM | IM | IM | PM | PM | PM | NM | NM | NM |
| NA18992 | IM | IM | IM | PM | PM | PM | NM | NM | NM |
| NA18565 | IM | IM | IM | IM | IM | IM | NM | NM | NM |
| NA18524 | NM | NM | NM | IM | IM | IM | NM | NM | NM |
| NA18942 | NM | NM | NM | PM | PM | PM | NM | NM | NM |
| NA11839 | NM | NM | NM | IM | IM | IM | NM | NM | NM |
| NA10851 | IM | IM | IM | PM | PM | PM | NM | NM | NM |
| NA19176 | NM | NM | NM | IM | IM | IM | NM | NM | NM |
| NA18509 | NM | NM | NM | IM | IM | IM | NM | NM | NM |
| NA19226 | UM | UM | UM | IM | IM | IM | NM | NM | NM |
| NA07357 | IM | IM | IM | PM | PM | PM | NM | NM | NM |
| NA07019 | IM | IM | IM | PM | PM | PM | NM | NM | NM |
| NA12873 | IM | IM | IM | PM | PM | PM | NM | NM | NM |
| NA19143 | NM | NM | NM | PM | PM | PM | NM | NM | NM |
| NA10847 | NM | NM | N/A | PM | PM | N/A | NM | NM | N/A |
| NA18518 | IM | IM | IM | IM | IM | IM | NM | NM | NM |
| NA21781 | NM | NM | NM | PM | PM | PM | NM | NM | NM |
| NA07348 | IM | IM | IM | PM | PM | PM | NM | NM | NM |
| NA19003 | NM | NM | NM | PM | PM | PM | NM | NM | NM |
| NA18544 | IM | IM | IM | IM | IM | IM | NM | NM | NM |
| NA18540 | IM | IM | IM | IM | IM | IM | NM | NM | NM |
| NA18966 | NM | NM | NM | IM | IM | IM | NM | NM | NM |

|  | ***SLCO1B1*** |  |  | ***TPMT*** |  |  | ***UGT1A1*** |  |  |
| --- | --- | --- | --- | --- | --- | --- | --- | --- | --- |
| **Coriell ID** | **Previous** | **WGS** | **ClinPharmSeq** | **Previous** | **WGS** | **ClinPharmSeq** | **Previous** | **WGS** | **ClinPharmSeq** |
| NA10831 | PIF | PIF | PIF | NM | NM | NM | PM | PM | PM |
| NA18855 | NF | NF | NF | IM | IM | IM | PM | PM | I [X] |
| NA18617 | NF | NF | NF | NM | NM | NM | IM | IM | IM |
| NA19908 | NF | NF | NF | NM | NM | NM | IM | IM | IM |
| NA18973 | NF | NF | N/A | NM | NM | N/A | IM | IM | N/A |
| NA12003 | DF | DF | DF | NM | NM | NM | IM | IM | IM |
| NA18519 | NF | NF | NF | NM | NM | NM | IM | IM | IM |
| HG00276 | DF | DF | DF | I | I | I | IM | IM | IM |
| NA11993 | DF | DF | DF | NM | NM | NM | IM | IM | IM |
| NA19917 | NF | NF | NF | NM | NM | NM | IM | IM | IM |
| NA19920 | NF | NF | NF | IM | IM | IM | IM | IM | IM |
| NA11832 | I | I | I | NM | NM | NM | IM | IM | IM |
| NA07029 | NF | NF | N/A | NM | NM | N/A | NM | NM | N/A |
| NA18868 | I | I | I | NM | NM | NM | PM | PM | I [X] |
| NA12813 | I | I | I | NM | NM | NM | PM | PM | PM |
| HG00589 | I | I | I | IM | IM | IM | NM | NM | NM |
| NA20296 | I | I | I | IM | IM | IM | NM | NM | NM |
| NA12717 | NF | NF | NF | NM | NM | NM | IM | IM | IM |
| NA07056 | PIF | PIF | PIF | NM | NM | NM | IM | IM | IM |
| NA18484 | I | I | I | NM | NM | NM | IM | IM | IM |
| NA19178 | NF | NF | NF | NM | NM | NM | IM | IM | IM |
| NA18564 | NF | NF | N/A | NM | NM | N/A | NM | NM | N/A |
| NA12145 | NF | NF | NF | NM | NM | NM | NM | NM | NM |
| NA18861 | PIF | I [X] | I [X] | NM | NM | NM | NM | NM | NM |
| HG00436 | NF | NF | NF | NM | NM | NM | IM | IM | IM |
| NA18552 | DF | DF | DF | NM | NM | NM | NM | NM | NM |
| NA07000 | DF | DF | DF | NM | NM | NM | NM | NM | NM |
| NA12006 | I | I | I | NM | NM | NM | IM | IM | IM |
| NA19007 | NF | NF | NF | NM | NM | NM | IM | IM | IM |
| NA19239 | NF | NF | NF | NM | NM | NM | PM | PM | PM |
| NA12156 | I | I | I | NM | NM | NM | IM | IM | IM |
| NA19147 | I | I | I | NM | NM | NM | PM | PM | I [X] |
| NA19095 | PIF | I [X] | I [X] | NM | NM | NM | IM | IM | IM |
| NA10854 | NF | NF | NF | NM | NM | NM | NM | NM | NM |
| NA18980 | NF | NF | NF | NM | NM | NM | PM | PM | PM |
| NA19207 | I | I | I | NM | NM | NM | IM | IM | IM |
| NA18526 | DF | DF | DF | NM | NM | NM | NM | NM | NM |
| NA18959 | NF | NF | N/A | NM | NM | N/A | NM | NM | N/A |
| NA06991 | PF | PF | PF | NM | NM | NM | NM | NM | NM |
| NA19109 | DF | DF | DF | NM | NM | NM | NM | NM | NM |
| NA18952 | NF | NF | N/A | NM | NM | N/A | NM | NM | N/A |
| NA19789 | NF | NF | NF | NM | NM | NM | NM | NM | NM |
| HG01190 | NF | NF | NF | NM | NM | NM | IM | IM | IM |
| NA19122 | NF | NF | NF | NM | NM | NM | IM | IM | IM |
| NA19819 | NF | NF | NF | NM | NM | NM | IM | IM | IM |
| NA19213 | PIF | PIF | PIF | NM | NM | NM | NM | NM | NM |
| NA07055 | PIF | PIF | PIF | NM | NM | NM | NM | NM | NM |
| NA19174 | I | I | N/A | NM | NM | N/A | IM | IM | N/A |
| NA20509 | DF | DF | DF | NM | NM | NM | IM | IM | IM |
| NA18992 | DF | DF | DF | NM | NM | NM | NM | NM | NM |
| NA18565 | NF | NF | NF | NM | NM | NM | NM | NM | NM |
| NA18524 | I | I | I | NM | NM | NM | NM | NM | NM |
| NA18942 | NF | NF | NF | NM | NM | NM | IM | IM | IM |
| NA11839 | NF | NF | NF | NM | NM | NM | NM | NM | NM |
| NA10851 | PIF | PIF | PIF | NM | NM | NM | NM | NM | NM |
| NA19176 | NF | NF | NF | I | I | I | IM | IM | IM |
| NA18509 | NF | NF | NF | NM | NM | NM | IM | IM | IM |
| NA19226 | PDF | PDF | PDF | NM | NM | NM | NM | NM | NM |
| NA07357 | DF | DF | DF | NM | NM | NM | IM | IM | IM |
| NA07019 | PIF | PIF | PIF | NM | NM | NM | NM | NM | NM |
| NA12873 | NF | NF | NF | NM | NM | NM | NM | NM | NM |
| NA19143 | NF | NF | NF | NM | NM | NM | IM | IM | IM |
| NA10847 | PF | PF | N/A | NM | NM | N/A | IM | IM | N/A |
| NA18518 | NF | NF | NF | NM | NM | NM | NM | NM | NM |
| NA21781 | PF | PF | PF | NM | NM | NM | IM | IM | IM |
| NA07348 | NF | NF | NF | NM | NM | NM | NM | NM | NM |
| NA19003 | NF | NF | NF | NM | NM | NM | NM | NM | NM |
| NA18544 | DF | DF | DF | NM | NM | NM | IM | IM | IM |
| NA18540 | DF | DF | DF | NM | NM | NM | IM | IM | IM |
| NA18966 | NF | NF | NF | IM | IM | IM | IM | IM | IM |

Abbreviations: PM, poor metabolizer; IM, intermediate metabolizer; NM, normal metabolizer; RM, rapid metabolizer; UM, ultrarapid metabolizer; PF, poor function; DF, decreased function; PDF, possible decreased function; NF, normal function; PIF, possible increased function; I, indeterminate.
