# Supplementary material for: ClinPharmSeq: A targeted sequencing panel for clinical pharmacogenetics implementation
Source: PLoS One. 2022 Jul 28;17(7):e0272129. doi: 10.1371/journal.pone.0272129 (PMC9333201; doi:10.1371/journal.pone.0272129)
Supplement: S6 Table — (DOCX) [file pone.0272129.s010.docx]

| No. | Coriell ID | Gene | Previous | WGS | ClinPharmSeq | Explanation |
| --- | --- | --- | --- | --- | --- | --- |
| 1 | NA19213 | *CYP2C19* | Normal Metabolizer | Indeterminate [X] | Indeterminate [X] | **39* has unknown function. |
| 2 | NA19143 | *CYP2C19* | Normal Metabolizer | Indeterminate [X] | Indeterminate [X] | **39* has unknown function. |
| 3 | NA18519 | *CYP2D6* | Normal Metabolizer | Indeterminate [X] | Indeterminate [X] | **106* has uncertain function. |
| 4 | NA19207 | *DPYD* | Normal Metabolizer | Intermediate Metabolizer [X] | Intermediate Metabolizer [X] | *c.557A>G* has decreased function. |
| 5 | NA06991 | *DPYD* | Normal Metabolizer | Intermediate Metabolizer [X] | Intermediate Metabolizer [X] | *c.2846A>T* has decreased function. |
| 6 | NA07055 | *DPYD* | Normal Metabolizer | Intermediate Metabolizer [X] | Intermediate Metabolizer [X] | *c.2846A>T* has decreased function. |
| 7 | NA18861 | *SLCO1B1* | Possible Increased Function | Indeterminate [X] | Indeterminate [X] | **32* has unknown function. |
| 8 | NA19095 | *SLCO1B1* | Possible Increased Function | Indeterminate [X] | Indeterminate [X] | **20* has unknown function. |
| 9 | NA18855 | *UGT1A1* | Poor Metabolizer | Poor Metabolizer | Indeterminate [X] | **80* (unknown function) was called instead of **80+*28* (decreased function) in ClinPharmSeq. |
| 10 | NA18868 | *UGT1A1* | Poor Metabolizer | Poor Metabolizer | Indeterminate [X] | **80* (unknown function) was called instead of **80+*28* (decreased function) in ClinPharmSeq. |
| 11 | NA19147 | *UGT1A1* | Poor Metabolizer | Poor Metabolizer | Indeterminate [X] | **80* (unknown function) was called instead of **80+*28* (decreased function) in ClinPharmSeq. |
